# *sept-1/zina-1* is an ancient toxin-antidote system in *Caenorhabditis elegans*

**DOI:** 10.1371/journal.pbio.3003563

**Published:** 2026-07-23

**Authors:** Dongyao Liu, Chaogu Zheng

**Affiliations:** School of Biological Sciences, The University of Hong Kong, Hong Kong SAR, China; University of Oxford Department of Biochemistry, UNITED KINGDOM OF GREAT BRITAIN AND NORTHERN IRELAND

## Abstract

The toxin-antidote (TA) systems, consisting of two tightly linked genes acting as a postzygotic distorter, are found in several eukaryotic organisms, including three androdioecious *Caenorhabditis* species. However, the evolutionary history of such TA systems remains poorly understood. Here, we report an ancient *C. elegans* TA system identified in the highly divergent Hawaiian strain XZ1516. The maternal toxin SEPT-1 induces a rod-like larval arrest phenotype in progeny that do not carry the antidote ZINA-1, which has 10 zinc finger motifs. Interestingly, *zina-1* is pseudogenized in most non-Hawaiian strains, while *sept-1* evolves from a toxic to a non-toxic form through sequence divergence. Phylogenetic studies found that among the three known TA systems *sept-1/zina-1* is the most prevalent one in the Hawaiian population that carries the ancestral variants, but it is no longer active in the more derived non-Hawaiian population. The loss of *sept-1/zina-1* coincides with the rise of *sup-35/pha-1* and *peel-1/zeel-1* in non-Hawaiian strains, suggesting dynamic evolution of the TA systems.

## Abstract 2

Toxin-antidote (TA) systems are found in several eukaryotic organisms, including *Caenorhabditis*nematodes. This study describes an ancient TA system in *C. elegans*; the maternal toxin SEPT-1 induces a rod-like larval arrest phenotype in progeny that do not carry the antidote ZINA-1.

## Introduction

The toxin-antidote (TA) system is a type of two-component selfish genetic element that promotes its own propagation but does not contribute to the fitness of the host [1,2]. The TA systems were first discovered in prokaryotes with the F-plasmid addiction in *E. coli* as a well-known example [3]. The plasmid carries a two-gene operon encoding a toxin CcdB that poisons the DNA gyrase and an antidote CcdA that disables the toxin; after cell division, only the daughter cells that inherit the plasmid can survive [4]. So far, hundreds of TA elements with different mechanisms of the toxin-antidote interactions have been found in prokaryotes [5]. In contrast, only a few TA elements are characterized in eukaryotes, including the spore killers in fungi, the gamete-killing meiotic drives in rice, the *Medea* factors in flour beetles, and the *peel-1/zeel-1* and *sup-35/pha-1* systems in nematodes [2].

In eukaryotes that undergo sexual reproduction, the TA system often operates by transmitting the toxin into the gametes through the cytoplasm and allowing the toxin to sabotage the development of the gamete or the fertilized egg in the absence of the genomically encoded antidote, which can block the effects of the toxin [6]. As a result, the toxin and antidote are usually coded by two closely linked genes in nearby loci, allowing them to be inherited together. In some surprising cases, they can also be coded by a single gene that exert both toxicity and resistance [6].

The nematode *Caenorhabditis elegans* has served as an important model organism for the study of the TA systems thanks to the extensive sampling efforts in the past few decades that isolated thousands of wild isolates of *C. elegans* [7–9]. By crossing the wild strains of *C. elegans* with the laboratory strain N2, previous studies discovered two pairs of TA elements that exist in N2 but not some wild strains. The *peel-1/zeel-1* system encodes a paternal toxin PEEL-1 which is deposited into the sperm and causes embryonic lethality if the offspring does not carry the antidote gene *zeel-1* [10,11]. The *sup-35/pha-1* system encodes a maternal toxin SUP-35 which is deposited into the oocyte and causes defects in organogenesis and lethality unless the offspring carries the antidote *pha-1* [12]. The toxicity and detoxification mechanisms of the two TA systems are not entirely clear, although a recent study found that PEEL-1 is likely a transmembrane cation channel that forms pores to damage the integrity of cell membrane [13]. It is also unclear whether there are more TA elements in addition to the two known ones and whether any wild strain carries TA elements that are not present in the laboratory standard N2 strain.

*C. elegans* offers tremendous opportunities to discover and understand TA elements in animals because of the availability of numerous wild strains, the ease of doing crosses and genetic manipulation, and the rich information on gene functions through decades of molecular studies. Moreover, self-fertilization of *C. elegans* to some extent might have prevented the fixation of TA elements in the species, allowing them to be identified through the hybridization of different strains. This idea is supported by recent studies that revealed the potentially widespread presence of TA elements in other self-fertilizing *Caenorhabditis* species, such as *C. tropicalis* and *C. briggsae* [14,15].

Several questions regarding the evolution of the TA systems can be addressed by identifying more TA systems. For example, where do they originate from and what are their evolutionary fate? How are the TA systems gained, maintained, and inactivated or replaced in natural populations? In particular, is the toxin lost mostly through deletion or pseudogenization or can the toxin gene evolve from a toxic to a non-toxic form? Do different TA systems show distinct patterns of ecological distribution? What is their evolutionary relationship? Can multiple TA systems co-exist in the same individuals or instead replace one another during evolution?

In this study, we address the above questions by identifying a novel TA system (named *sept-1/zina-1*) in a highly diverged *C. elegans* wild isolate XZ1516. SEPT-1 is a maternally deposited toxin that disrupts the function of the intestine, whereas ZINA-1 is the antidote that blocks the effects of SEPT-1. Interestingly, *zina-1* is pseudogenized in N2, while *sept-1* was kept and evolved into a largely non-toxic form, as *sept-1(N2)* only shares 47% identity with *sept-1(XZ1516)*. Phylogenetic analysis showed that *sept-1/zina-1* mostly exists in the Hawaiian strains that carry the most ancestral variants. This ancestral TA system disappeared entirely in the non-Hawaiian strains, and its loss coincides with the rise of another maternal TA system *sup-35/pha-1*.

## Results

### Genetic mapping of a new toxin-antidote element in a divergent Hawaiian strain

**Fig 1:**
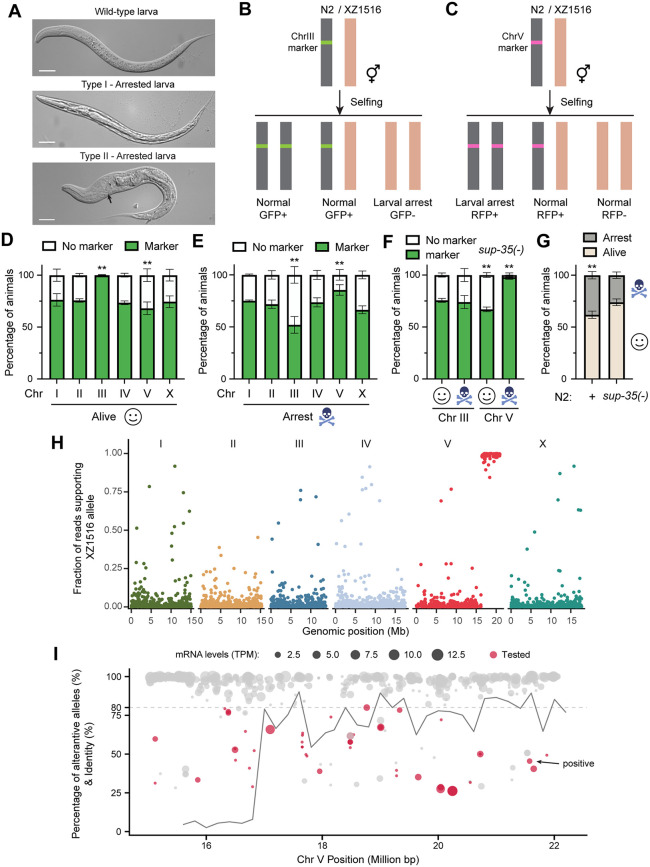
Mapping of a novel TA system in XZ1516. **(A)** Arrested larvae among the offspring of N2/XZ1516 hybrids. Type I represents the rod-like arrested larva, while type II represents the curled-up larva with deformed pharynx (arrow). Scale bars, 20 μm. **(B, C)** Schematic cartoon of the cross and phenotypes of the progeny of the N2/XZ1516 hybrids. Gray bars indicate the N2 chromosomes carrying fluorescent reporters as transgenes, and brown bars indicate the homologous XZ1516 chromosomes. **(D, E)** Percentages of live or arrested animals that carry the N2-derived fluorescent marker for each chromosome among the progeny of the N2/XZ1516 hybrids. Seventy-five percent animals were expected to carry the dominant marker if it is not linked to the TA system. **(F)** Percentages of live or arrested animals that carry the N2 chrIII or chrV marker among the progeny of N2[*sup-35(unk210)*]/XZ1516 hybrids. **(G)** Percentages of animals that are alive or arrested among the progeny of N2/XZ1516 or N2[*sup-35(unk210)*]/XZ1516 hybrids. In (D–G), the mean ± SD of 3–5 biological replicates (*N* ≥ 200 for each) were shown; double asterisks indicate statistically significant (*p* < 0.01) deviation from the expected 75% for animals carrying the marker or being alive in a Chi-squared test. **(H)** Whole-genome sequencing results of the near-isogenic lines (NILs) obtained by introgressing the XZ1516-derived TA locus into N2. Reads were mapped to a N2 genome scaffold installed with XZ1516 SNPs and small indels, and the proportion of the reads supporting XZ1516 alleles were plotted. **(I)** A zoom-in view of the right arm of chrV of a fully assembled XZ1516 genome. Genes in this region were presented as solid dots. The ones that code for proteins with > 100 amino acids, have detectable expression in the XZ1516 transcriptome, and are diverged (< 80% identity) from the N2 genes were marked in red and subjected to RNAi screen. The positive hit is indicated by the arrow. The size of the dots represents mRNA expression levels. The solid line represents the frequency of alternative alleles (N2 is the wild type) in the NILs; bin size is 200 kb. Some images were created in BioRender. Zheng, C. (2026) https://BioRender.com/5fjhayv. The data underlying this Figure can be found in S1 Data.

When crossing the laboratory standard Bristol strain N2 with a highly divergent Hawaiian strain XZ1516, we noticed that although the N2/XZ1516 hybrids (F1) developed normally, about ~40% of their progeny (F2) from self-fertilization either died at late embryonic stages or arrested at early larval stages. Two types of larval arrest phenotypes were observed: one showed a rod-like shape, while the other showed a more curled shape with a malformed pharynx (Fig 1A). We then crossed N2 that carried different chromosomal markers with XZ1516 and found that the surviving offspring of the N2/XZ1516 hybrids must carry the chromosome III (chrIII) of N2 and chrV of XZ1516 (Fig 1B–1E), suggesting the presence of two independently segregating TA systems in the hybrid. Between the two identified TA systems in *C. elegans* [10,12], *zeel-1/peel-1* system on chrI is conserved between N2 and XZ1516, but *sup-35/pha-1* locus on chrIII shows divergence (S1A and S1B Fig). In the XZ1516 genome, we found inversion and duplication events that led to sequence divergence of the toxin *sup-35* and a deletion event that likely inactivated the antidote *pha-1*. To test whether the preferred inheritance of N2 chrIII was caused by the *sup-35/pha-1* locus that was potentially nonfunctional in XZ1516, we deleted *sup-35* in N2 carrying a chrIII marker and found that the bias towards N2 chrIII disappeared among the offspring of the N2/XZ1516 heterozygotes (Figs 1F and S1C). The lethality rate dropped to ~25% (Fig 1G) and the arrested larvae all appeared to be rod-like. These results indicated that there remained one other TA element, which was present on the chrV of XZ1516 and absent in N2. This TA system is likely new, since no chrV-associated TA was identified before.

To understand the inheritance pattern of the new toxin, we crossed hybrid hermaphrodites with N2 males and found arrested larvae in the cross progeny, but the reciprocal cross using hybrid males and N2 hermaphrodites did not produce any arrested animals, suggesting that the toxin is maternally transmitted through the oocyte and its toxicity can be suppressed by a zygotic copy of XZ1516 chrV-linked antidote (S1D and S1E Fig). In the above experiments, we crossed N2 males with XZ1516 hermaphrodites to generate the N2/XZ1516 hybrids. Since previous studies in *C. tropicalis* found that cross direction can critically affect TA activities [16], we also generated the N2/XZ1516 hybrids by crossing XZ1516 males with N2 hermaphrodites. The same rod-like phenotypes were observed among the progenies of these hybrids, suggesting that this TA is active regardless of whether it is maternally or paternally inherited.

To map the newly found TA system, we introgressed the TA locus into the N2 strain and generated near-isogenic lines (NILs) that carry the XZ1516-derived TA element in the N2 genomic background. Through whole-genome sequencing and SNP mapping (see the Materials and methods for details), we mapped the TA element to a 5-Mb region on the right arm of chrV (Fig 1H). Using a chromosome-level XZ1516 genome, we predicted genes in this region and a nearby 2-Mb region (Chr V: 15–22 Mb in total) and found 458 genes that code for proteins longer than 100 amino acids and had detectable mRNA expression (FPKM > 0) in the transcriptome (Fig 1I). From these genes, we compiled a list of 46 candidate genes with low or no homology (< 80% identity) with N2 genes, assuming that the TA element is absent or highly diverged in the N2 strain (S1 Table). We then created RNAi constructs expressing dsRNA against these candidate genes and transformed them into bacteria to allow the delivery of the dsRNA into XZ1516 strain through feeding RNAi. We expected silencing of the antidote gene to cause lethality in XZ1516 due to the unsuppressed toxicity from the toxin.

**Fig 2:**
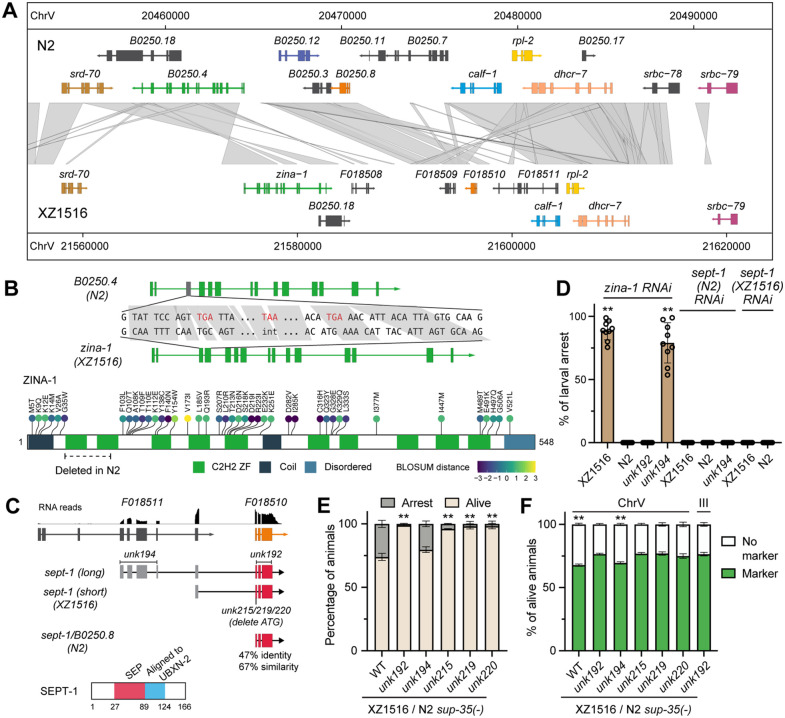
Identification of *zina-1* as the antidote gene and *sept-1* as the toxin gene. **(A)** A zoom-in view of the potential TA locus in XZ1516 and its comparison with N2 genome. *F108508*-*F108511* were de novo annotated by Funannotate. (B) *zina-1(XZ1516)* contains 15 exons and is pseudogenized in N2 due to the deletion of exon 3 and part of exon 4, which led to frameshift and premature stop codons (in red) from exon 3 onward. *zina-1* in XZ1516 codes for a 548 a.a. protein with 10 zinc finger motifs. In addition to the deletion in N2 (dashed line), the ZINA-1 coding sequence carries many missense variants (color-coded by BLOSUM distance) in N2 compared to XZ1516. **(C)** RNA reads supporting the exons of *F108510* and *F108511* were shown. *sept-1* long isoform contains a 5′UTR formed by five noncoding exons and its short isoform has only one noncoding exon. It shares 47% identity and 67% similarity with B0250.8 in N2. SEPT-1 is a 166 a.a. protein with a putative SEP domain (based on similarity with *C. elegans* UBXN-2). Molecular lesions of various *unk* alleles were shown. **(D)** Rates of lethality caused by RNAi against *zina-1*, *sept-1(XZ1516)*, and *sept-1(N2)* in various strains (*N* ≥ 100 for each data point). Double asterisks indicate statistically significant (*p* < 0.01) deviation from 0% (found in XZ1516 and N2 controls) in a Chi-squared test. **(E)** Percentages of arrested animals among the progeny of the hybrids between XZ1516 *sept-1* mutants and N2 *sup-35(-)*. **(F)** Percentages of live animals carrying chrIII or chrV markers among the progeny of the hybrids between XZ1516 *sept-1* mutants and N2 *sup-35(-)*. In (E, F), the mean ± SD of 3–4 biological replicates (N ≥ 100 for each) were shown; double asterisks indicate statistically significant (*p* < 0.01) deviation from the expected 75% for animals being alive or carrying the marker in a Chi-squared test. The data underlying this Figure can be found in S1 Data.

From this RNAi screen, we identified only one gene, whose knockdown led to the rod-like larval arrest phenotype similar to the arrested offspring of the N2/XZ1516 hybrid (S2A Fig). Synteny analysis showed that this gene was aligned to the N2 pseudogene *B0250.4* (Fig 2A). In XZ1516, it is a 15-exon gene encoding a 548-amino acid protein with 10 C2H2-type zinc finger motifs, so we named this gene *zina-1* for “*zin*c finger-containing *a*ntidote *1*”. Sequence alignment suggests that *zina-1* is pseudogenized in N2 due to the deletion of exon 3, intron 3, and part of exon 4, leading to a frameshift and premature stops (Fig 2B). The rest of the exons have very high identity (>95.6%) between *zina-1* and N2 *B0250.4*, denoted as *zina-1(N2*ψ), although there are still some missense variations (Fig 2B).

### Identifying a SEP domain-containing protein SEPT-1 as the toxin in the TA element

We next searched for the toxin gene based on the assumption that the toxin is closely linked to the antidote. Homology-based annotation identified *srd-70*, *B0250.18*, *calf-1*, *rpl-2*, *dhcr-7*, and *srbc-79* in the genomic region flanking *zina-1*, but they are all highly similar between N2 and XZ1516 and not likely to be the toxin. We then used Funannotate [17] to perform de novo gene prediction and identified four additional protein-coding genes (*F018508 to F018511*) in this region. *F018508* had no supporting RNA-seq reads, and *F018508* showed homology with N2 *rpl-2*. *F018510* showed homology with N2 *B0250.8*, while *F018511* overlapped with *calf-1*. Since there are RNA-seq reads connecting *F018510* and *F018511*, we used forward primers that bind to spliced leaders (SL1 and SL2) and reverse primers that bind to *F018510* exon 3 to obtain full-length transcripts of *F018510* and indeed found two isoforms containing exons from *F018511* (Fig 2C). Nevertheless, these exons appeared to be noncoding and likely serve as 5′UTR. Based on RNA-seq reads, the long isoform is the minor isoform (S2C Fig).

We then deleted *F018510* coding sequence in XZ1516 and conducted RNAi against the antidote *zina-1* in the knockout mutants (*unk192*) and did not observe any arrested larvae. In contrast, deleting four exons encoding the 5′UTR of the long isoform (*unk194* allele) did not affect the lethality in *zina-1* RNAi animals (Fig 2D). These results suggest that *F018510* codes for the toxin. Since both XZ1516 *F018510* and N2 *B0250.8* code for proteins with a putative SEP domain, we named the *F018510* gene *sept-1* for “*SEP* domain-containing *t*oxin *1*”. In the following text, we refer to the *B0250.8* gene in N2 as *sept-1(N2)*, which is highly diverged from *sept-1(XZ1516)* with only 47% identity. Since the antidote is pseudogenized in N2, we hypothesize that the presumptive toxin *sept-1* may no longer be toxic in N2 due to either functional divergence or loss of expression. The function of SEP domain is currently unclear, although one study suggests that it may inhibit lysosomal cysteine protease cathepsin L [18]. SEP domain is almost always followed by a UBX domain in proteins, and indeed, the sequence following the SEP domain in SEPT-1 is aligned to *C. elegans* UBXN-2 (Fig 2C).

To further confirm that *sept-1* is the toxin, we crossed XZ1516 *sept-1(unk192)* with N2 *sup-35(-)* and found that since both toxins are removed, the preference for N2 chrIII and XZ1516 chrV was eliminated among the offspring of the hybrids, and the fluorescent markers on both chromosomes were normally inherited by 75% of the progeny (Fig 2E and 2F). To further confirm that the SEPT-1 protein and not its mRNA is toxic, we created three alleles (*unk215*, *unk219*, and *unk220*) that only deleted the start codon of *sept-1* and a few flanking nucleotides (S2B Fig) and found that they indeed removed the maternal toxicity of the N2/XZ1516 hybrids (Fig 2E and 2F).

After mapping the TA system, we used genotyping as a more accurate method to confirm its inheritance pattern. First, in the XZ1516 background, deleting the TA on the chrV led to the loss of that chromosome in surviving offspring of the heterozygotes; the same is true for the N2/XZ1516 hybrid and the N2 backgrounds (S3A Fig). Thus, the TA element mandates its own propagation regardless of the genetic environment. Second, we confirmed that the toxin was maternally deposited into the oocytes and paternal inheritance did not cause any lethality (S3B Fig).

While we were conducting this work, the Kruglyak lab reported a TA system in XZ1516, which was discovered by crossing it with the QX1211 strain [19]. They named the system *mll-1/smll-1*, which is the same as the *sept-1/zina-1* locus. However, our characterization of this TA system differs significantly from theirs (see below).

### ZINA-1 activity is lost and SEPT-1 toxicity strongly attenuated in N2

**Fig 3:**
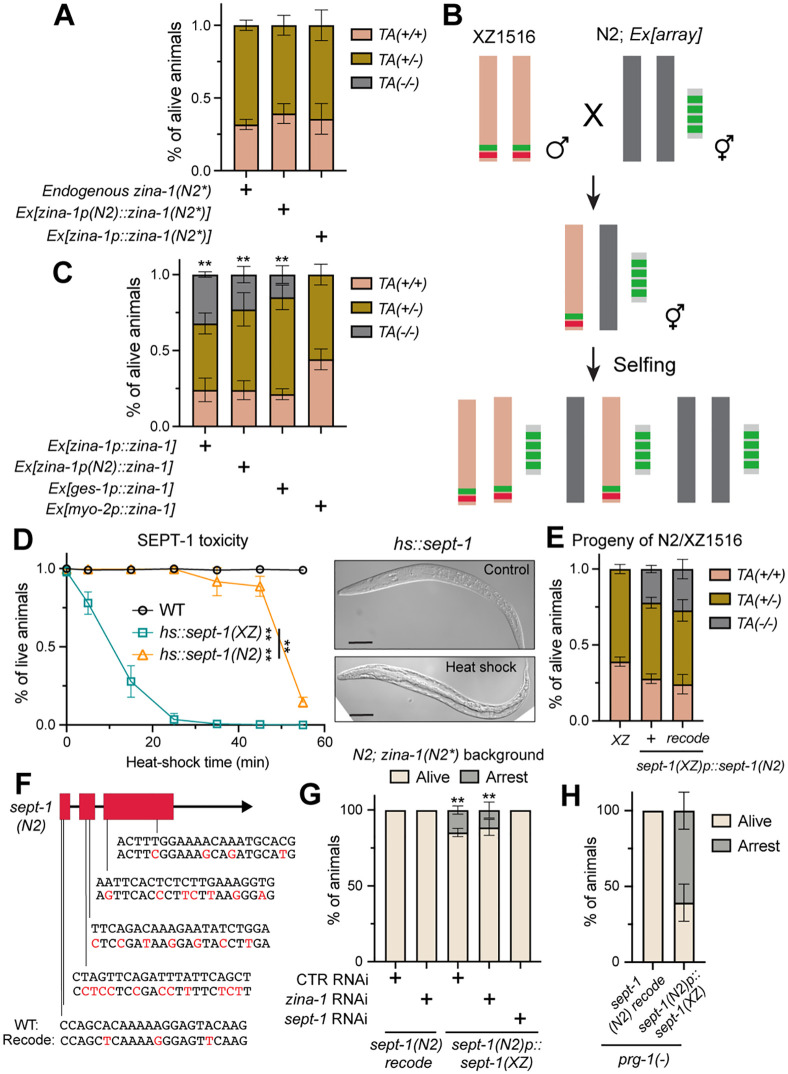
SEPT-1(XZ1516) proteins are more toxic than SEPT-1(N2). **(A)** Percentages of live animals with specific genotypes (determined by single-worm PCR) among the progeny of N2/XZ1516 hybrid that carry the repaired *zina-1(unk232)* allele from N2, denotated as zina-1(N2*), or extrachromosomal arrays expressing zina-1(N2*). It should be noted that the zina-1(N2*) expressed from *zina-1p(N2)* contained all the variants from the exon 3 and 4 of XZ1516. **(B)** Schematic cartoon of the experiments. Only live progeny that carried the extrachromosomal array were subjected to genotyping. **(C)** Percentages of live animals with specific genotypes among the progeny of N2/XZ1516 hybrid carrying the indicated extrachromosomal arrays. Mean ± SD of 3 biological replicates (*N* ≥ 50 for each) were shown (the same for E). Double asterisks indicate statistically significant deviation (*p* < 0.01) from the expected 2:1 ratio for TA(+/-):*TA(+/+)* in a Chi-square test. **(D)** Percentages of L1 animals (in N2 background) carrying the *unkSi32[hs::sept-1(XZ1516)]* and *unkSi18[hs::sept-1(N2)]* that were still alive at 24 hours after a 5, 15, 25, 35, 45, and 55-min heat-shock. Representative images of *unkSi32* animals that were arrested after a 45-min heat-shock (scale bars, 20 mm). Three to five replicates (*N* ≥ 100) were carried out; double asterisks indicate significant difference (*p* < 0.01) between two curves in a Kolmogorov–Smirnov test. Two independent lines were tested and similar results were obtained. **(E)** Percentages of live animals with the indicated genotypes among the progeny of N2/XZ1516 hybrid that carry the wild-type *sept-1(XZ1516)* or *sept-1(unk223)*, which is *sept-1(XZ1516)p::sept-1(N2)*, or *sept-1(unk243)*, which is *sept-1(XZ1516)p::sept-1(N2)_recode*, from XZ1516. **(F)** The predicted piRNA binding sites in the coding region of *sept-1(N2)*, which were mutated in the *sept-1(N2)_recode* version. Red letters indicate the synonymous mutations. **(G)** Percentages of animals that were arrested in N2 carrying the *sept-1(N2; unk277, recode)* or *sept-1(N2; unk257)* allele, which is *sept-1(N2)p::sept-1(XZ1516)*, and subjected to various RNAi treatment. All animals carry the *zina-1(unk232*, N2*) background. Double asterisks indicate statistically significant (*p* < 0.01) deviation from 0% for larval arrest in a Chi-squared test; *N* ≥ 100 for each of the five replicates. **(H)** The same as (G) except the animals also carry *prg-1(n4503)* alleles. Details of the alleles used in this Figure can be found in S4 Fig. The data underlying this Figure can be found in S1 Data.

Since *zina-1(N2*ψ) appeared to be pseudogenized by a deletion of exon 3 and 4, we repaired the locus by inserting a 190-bp fragment of *zina-1* coding sequence to restore the open reading frame (S4A Fig). Surprisingly, this repaired allele, denoted as zina-1(N2*), did not provide any protection against *sept-1* toxicity, as none of the surviving offspring of N2/XZ1516 hybrid carried the allele (Fig 3A). We also used extrachromosomal array to overexpress zina-1(N2*) from either the *zina-1(N2*ψ) promoter or the XZ1516 *zina-1* promoter; neither array was able to inhibit the maternal SEPT-1 (Fig 3A and 3B). As controls, we expressed *zina-1* from the two promoters and both were able to protect the animals that do not carry the antidote gene in the genome (Fig 3C). This result suggests that the missense mutations in zina-1(N2*) may have rendered the protein inactive even if the deletion did not occur. In fact, many mutations affected residues in the zinc finger domains, potentially disrupting functions (Fig 2B).

A puzzling issue of the *sept-1/zina-1* system is that the toxin is kept in N2 where the antidote is lost. To assess and compare the toxicity of SEPT-1(XZ1516) and SEPT-1(N2) proteins, we used the MosTI approach [20] to construct animals carrying a single-copy transgene that expressed *sept-1(XZ1516)* or *sept-1(N2)* from a heat-shock promoter in N2 at exactly the same genomic locus (S4B Fig). Heat induction (at 33 °C) of 30 min produced little lethality with SEPT-1(N2), whereas the same condition caused almost 100% lethality by SEPT-1(XZ1516) (Fig 3D). Although prolonged induction of SEPT-1(N2) expression could still cause larval arrest, its toxicity was much weaker than SEPT-1(XZ1516) in the N2 background. Using RT-qPCR, we found that the heat-shock promoter induced similar mRNA expression of *sept-1(N2)* and *sept-1(XZ1516)* (S5A Fig), but western blot results indicated that the same heat-shock condition resulted in higher expression of SEPT-1(XZ1516) proteins than SEPT-1(N2) (both are fused with HA-tags; S5B Fig). We reason that the SEPT-1(N2) proteins may be less stable than SEPT-1(XZ1516), which could contribute to the lower toxicity. Forced expression of *sept-1(XZ1516)* resulted in less lethality in L4 animals than in L1 animals, suggesting that older animals are more resistant to *sept-1* toxicity (S5C Fig). Nevertheless, we still observed significantly weaker toxicity from *sept-1(N2)* compared to *sept-1(XZ1516)*. When examining the arrested animals after the heat-shock, we noticed that they had thinner intestine than wild-type animals, and the phenotype was more pronounced in animals carrying the *hs::sept-1(XZ1516)* transgene than the ones carrying *hs::sept-1(N2)* (S5D Fig). This result suggested that somatic expression of the toxin may disrupt the function of the intestine.

To test the potential toxicity of SEPT-1(N2) in the XZ1516 background, we swapped out *sept-1(XZ1516)* coding region with *sept-1(N2)* sequences in the XZ1516 genome and crossed the edited XZ1516 with N2 (S4C Fig). We did not observe any lethality among the offspring of the hybrid and the edited TA elements followed the Mendelian segregation pattern (Fig 3E), suggesting that *sept-1(N2)* has no toxicity if expressed from the endogenous *sept-1(XZ1516)* promoter. We reason that SEPT-1(N2) has lost most of its toxicity due to protein sequence divergence, which allows *zina-1* to be pseudogenized by genetic drift.

The report by Zdraljevic and colleagues from the Kruglyak lab suggests that *sept-1(N2)* (referred to as *B0250.8* in their paper) is not toxic because its expression in N2 is suppressed by small RNAs [19]. Specifically, they propose that piRNA binding to the *sept-1(N2)* transcripts would lead to perpetual silencing by the 22G endo-siRNA pathway [19]. To formally test this idea, we replaced the *sept-1(N2)* coding region with recoded sequences that altered most codons through synonymous mutations. This recoding likely removed the potential piRNA binding sites in the coding sequence and would, in theory, allow *sept-1(N2)* to be expressed and its possible toxicity to be tested (Fig 3F). In fact, using RT-PCR we were able to detect the expression of the recoded *sept-1(N2)*, while wild-type *sept-1(N2)* was not detectable. However, we did not observe any larval arrest phenotype in the recoded animals (Fig 3G). Since we did the gene editing in the zina-1(N2*) background, we also knocked down *zina-1* and did not observe any lethality either. To further rule out the effects of piRNAs, we crossed the above animals with *prg-1(-)* mutants (*prg-1* codes for the only Piwi-family Argonaute [21] in *C. elegans*) but still did not find any lethality caused by *sept-1(N2)* (Fig 3H). Moreover, we created animals that expressed the recoded *sept-1(N2)* from the endogenous *sept-1(XZ1516)* promoter in XZ1516 and crossed the edited XZ1516 with N2 and did not find arrested larvae among the progeny of the N2/XZ1516 hybrids (Figs 3E and S4C). Thus, we concluded that the lack of toxicity from the *sept-1(N2)* locus is not likely due to small RNA-mediated mechanisms but rather the non-toxic nature of the SEPT-1(N2) protein.

As a control, we replaced the *sept-1(N2)* coding region with *sept-1(XZ1516)* sequences and indeed observed rod-like arrest phenotype in ~15% of the animals, supporting the idea that *sept-1(XZ1516)* is more toxic than *sept-1(N2)* (Fig 3G). Interestingly, the lethality caused by *sept-1(XZ1516)* expressed from the *sept-1(N2)* locus was enhanced in the *prg-1(-)* background (Fig 3H). We reason that there may be some small RNAs targeting the *sept-1(XZ1516)* coding region or the unaltered *sept-1* UTR region in N2.

### 3′UTR of the *sept-1* mRNA has a stem-loop structure and is A-to-I edited

**Fig 4:**
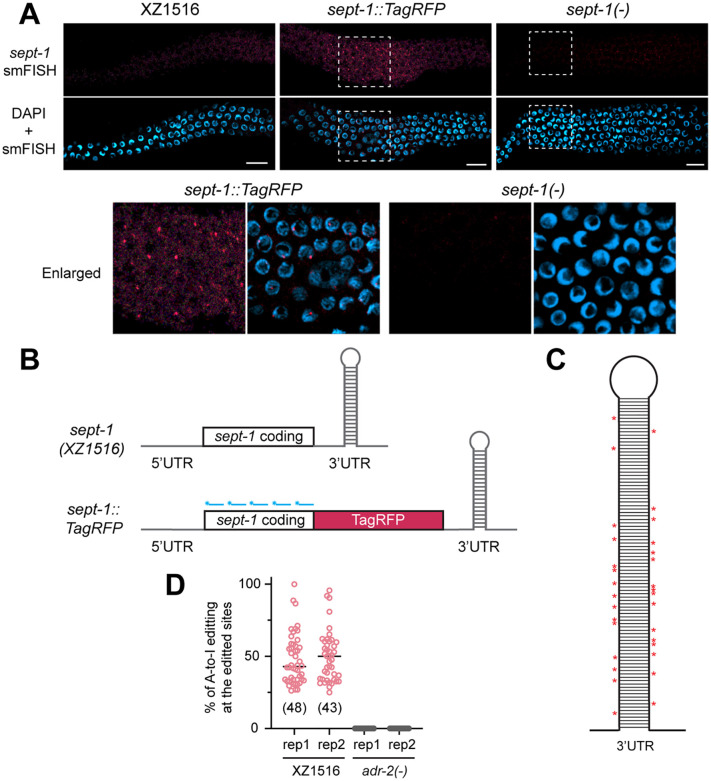
*sept-1(XZ1516)* 3′UTR forms a hairpin structure and undergoes A-to-I editing. **(A)** smFISH signal against the *sept-1(XZ1516)* coding region in dissected gonads of the wild-type, *sept-1(unk247; sept-1::TagRFP)*, and *sept-1(unk192)* XZ1516 adult animals. Scale bars, 20 μm. *sept-1(unk192)* is the *sept-1(-)* allele and serves as a negative control. The dashed boxes are enlarged in the bottom images. **(B)** A schematic cartoon showing the difference between the mRNAs produced by wild-type *sept-1(XZ1516)* and *sept-1(unk247; sept-1::TagRFP)* loci. The short blue lines indicate the smFISH probes. **(C)** A schematic presentation of the positions of A-to-I editing in the stem structure of the *sept-1* 3′UTR based on published RNA-seq results (NCBI SRA: SRR12868314). Each asterisk indicates one edited nucleotide. The number of steps on the ladders does not accurately reflect the number of base pairings in the hairpin; the scheme is intended to show the rough positions of the edits. **(D)** Percentages of RNA reads that show the A-to-I editing (read as a G) for each edited site for XZ1516 wild-type and *adr-2(unk280)* animals based on the RNA-seq data generated in this study. Two biological replicates identified 48 and 43 edited sites, respectively, in XZ1516 wild-type animals; no editing was found in *adr-2* mutants. The data underlying this Figure can be found in S1 Data.

We next studied the expression pattern of the toxin and antidote. First, we designed single-molecule fluorescent in situ hybridization (smFISH) probes against *sept-1* mRNA, but could not detect any signal in the gonad or in early embryos of XZ1516 animals. We then created an endogenous TagRFP knock-in at the *sept-1* locus in XZ1516 but failed to observe any TagRFP signals in early embryos. Antibodies staining against the HA tag fused to the C-terminus of the SEPT-1::TagRFP did not generate any signal either. Surprisingly, the smFISH against *sept-1(XZ1516)* coding sequence gave some signals in the gonad of *sept-1(XZ1516)::TagRFP* animals (Fig 4A). These results might suggest that the *sept-1* mRNA but not proteins are maternally deposited into the oocyte. Our finding is generally consistent with the report by Zdraljevic and colleagues [19], although they were able to directly detect the presence of *sept-1* transcripts with smFISH in the early embryos of XZ1516.

We do not know why our *sept-1(XZ1516)* probes did not produce any signal in wild-type XZ1516 animals, but upon close inspection of the *sept-1(XZ1516)* mRNA sequence, we found a very long stem-loop structure in the 795-nt 3′UTR region (Fig 4B). The entire 3′UTR of *sept-1(XZ1516)* is mostly a palindromic sequence that folds into a hairpin structure with a 315-nt-long stem and a 7-nt unpaired loop according to a structure predicted by “RNA fold” (http://rna.tbi.univie.ac.at/cgi-bin/RNAWebSuite/RNAfold.cgi). In comparison, the coding region of *sept-1* is merely 501-nt-long. Thus, we speculate that the proximity to the hairpin-structured 3′UTR may somehow render the *sept-1(XZ1516)* coding sequence inaccessible by the smFISH probes but inserting a 711-nt-long TagRFP sequence between the *sept-1* coding and 3′UTR regions might free up the coding region for the binding by the smFISH probes (Fig 4B).

Interestingly, when examining the published RNA-seq data (NCBI SRA: SRR12868314) of XZ1516, we found 31 “G to A” mismatches between the mRNA sequence and the genomic sequences in the *sept-1(XZ1516)* 3′UTR (Fig 4C). Even more mismatched sites were identified from the RNA-seq data generated in this study (Fig 4D). Since inosine (I) is typically read as a guanine (G) in DNA sequencing, we concluded that the *sept-1(XZ1516)* 3′UTR underwent extensive A-to-I editing, and this editing appeared to occur as clusters in the double-stranded region of the 3′UTR on both the ascending and descending arms of the stem (Fig 4C). Previous studies found that A-to-I editing by adenosine deaminases (ADARs) enhanced the mRNA levels of germline and neuronal genes [22,23]. We found that the *sept-1(XZ1516)* mRNA levels only decreased by 5% (not significant) in *adr-2(-)* mutants, which deleted the only active adenosine deaminase in *C. elegans* [23] and removed all mismatches of RNA to DNA sequence in *sept-1* 3′UTR (Fig 4D). Moreover, the loss of A-to-I editing did not affect *sept-1* toxicity, since *zina-1* RNAi in the *adr-2(-)* mutants still resulted in rod-like larval arrest. Thus, the editing of *sept-1(XZ1516)* RNA is not essential for its expression and toxicity.

Single-copy *sept-1(XZ1516)p::mScarlet3* and *sept-1(N2)p::mScarlet3* transcriptional reporters did not show any fluorescent signals, so we generated multicopy extrachromosomal arrays for these reporters instead. Since the arrays were known to be often silenced in the germline [24], we only detected somatic expression of *sept-1(XZ1516)* and *sept-1(N2)* in the pharynx and intestine (S6 Fig). The expression level of *sept-1(XZ1516)* appeared to be much higher than *sept-1(N2)* in both XZ1516 and N2 backgrounds, suggesting that *sept-1(XZ1516)* promoter has stronger activity compared to *sept-1(N2)* promoter likely due to considerable sequence divergence (49.3% identity for the 2-kb promoter region). Although somatic expression of the toxin is not necessary for the propagation of a classical TA, it remains unclear whether the expression of *sept-1* in the digestive tract enhances its toxicity or has other functions.

### ZINA-1 protects the development of the intestine by suppressing the SEPT-1 toxicity

**Fig 5:**
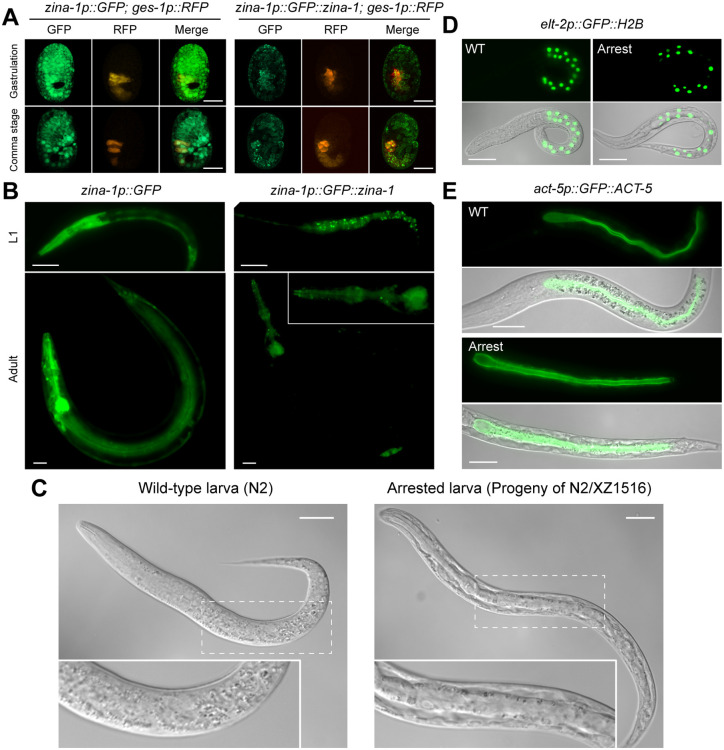
*zina-1* suppresses *sept-1* toxicity in the intestine. **(A)** The expression of *zina-1* transcriptional and translational reporters in the embryos at gastrulation and comma stages. The E lineage that forms the intestine is labeled by TagRFP expressed from *ges-1* promoter. **(B)** The expression of *zina-1* reporters at L1 and adult stages. The pharynx part of the adult image of *zina-1p::GFP::zina-1* is enlarged in the box. **(C)** Representative DIC images of a wild-type N2 larva at L1 stage and an arrested larva among the progeny of the N2/XZ1516 hybrid. Dashed boxes are enlarged in the inserts showing the shrinkage of the intestine in the arrested larva. **(D, E)** Wild-type N2 larva and arrested larva among the F2 progeny of the N2/XZ1516 hybrid carrying the *caIs71[elt-2p::GFP::HIS-2B; rol-6(D)]* and the *jyIs13 [act-5p::GFP::act-5]* transgenes, respectively. Scale bars represent 20 μm in all panels.

To examine *zina-1* expression, we constructed both transcriptional and translational GFP reporters of *zina-1* in XZ1516. Widespread expression was detected in the embryos as early as gastrulation and proceeded through comma stages. The GFP::ZINA-1 translational fusion showed enrichment in the developing E lineage which was marked by *ges-1p::TagRFP* and formed the intestine (Fig 5A). In L1 and adult animals, we observed *zina-1p::GFP* and *zina-1p::GFP::zina-1* expression mostly in the pharynx and intestine (Fig 5B). The difference in the GFP signals between the transcriptional and translational reporters may reflect the instability of the antidote protein, which was found in other TA systems as well [25].

The enrichment of ZINA-1 in the digestive system in XZ1516 led us to hypothesize that ZINA-1 may mostly function in the intestine to detoxify SEPT-1. In fact, when we examined the arrested larva caused by the SEPT-1 toxicity, we observed abnormal intestinal morphology under the differential interference contrast microscopy, as the intestinal cells became thinner and separated from the body wall muscles (Fig 5C). The arrested larva among the progeny of N2/XZ1516 hybrids also appeared to have bloated intestine, indicating digestion failure. Although the number of intestinal cells (labeled by *elt-2p::GFP::H2B*) were largely normal in the arrested animals, we confirmed the appearance of bloated intestinal lumen using GFP::ACT-5 that labels the apical domain of the intestinal cells (Fig 5D and 5E). These results indicated that intestinal development and/or function is disrupted by SEPT-1 in the absence of ZINA-1.

To confirm ZINA-1 functions in the intestine, we constructed extrachromosomal array that expressed *zina-1* from the intestine-specific *ges-1* promoter in N2 and crossed the animals with XZ1516. We found that if the offspring of the N2/XZ1516 hybrids carried the extrachromosomal array, they can survive even if they did not have XZ1516 chrV (Fig 3C). In contrast, pharyngeal expression of *zina-1* from the *myo-2* promoter in the extrachromosomal array could not protect the animals that did not carry the genomic *zina-1* (Fig 3C), suggesting that ZINA-1 must act in the intestine to detoxify SEPT-1.

### Distribution of the three TA systems among the wild populations of *C. elegans*

**Fig 6:**
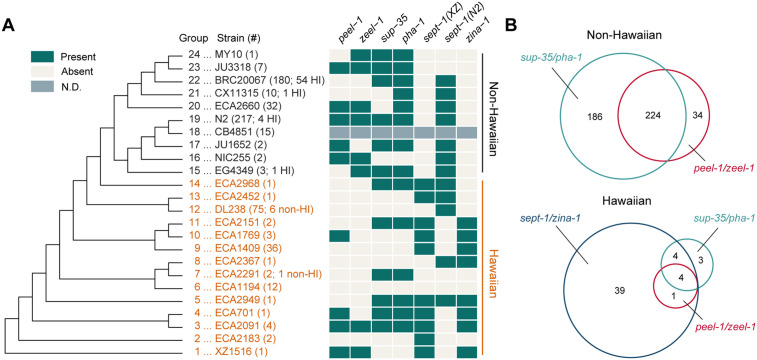
Evolutionary history of the three TA systems in *C. elegans.* **(A)** A phylogenetic tree of representative wild isotypes from 24 groups with unique genotypes of the three TA systems. This tree is extracted from a bigger phylogenetic tree of 611 *C. elegans* wild isotypes (S7 Fig) constructed using optimal comminated bayes models selected by ModelFinder [39]. Strains that were physically isolated from Hawaiian Islands are labeled in orange. The number of strains from each group is labeled in paratheses. Some groups contain both Hawaiian (HI) and non-Hawaiian strains (non-HI); the identity of the group is determined by the majority of the strains, and the number of minority strains were also indicated in the paratheses. Strain information for each group is shown in S2 Table. The genotypes for both the toxin and antidote in the three TA systems were annotated (see Materials and methods for the details of genotype calling). “N.D.” means “not determined” since the genotype is missing. **(B)** The overlapping among the three functional TA systems in the Hawaiian and non-Hawaiian populations were shown in the Venn diagrams. The number of strains that carry the corresponding TA system is shown.

The fact that N2 and XZ1516 can poison each other using two different maternal TA systems prompted us to examine the distribution of the three known TA systems in the global population of *C. elegans*. We created a computational pipeline to map the presence and absence (or divergence) of the *peel-1/zeel-1*, *sup-35/pha-1*, and *sept-1/zina-1* systems among 611 wild isotypes, which were divided into 24 groups based on unique genotypes for toxins and antidotes and positions on the phylogenetic tree (Figs 6A and S7; S2 Table; see Materials and methods for details). The tree was built using 4,736 single-copy genes defined by previous studies [26] and showed the clustering of Hawaiian strains, which were known to carry ancestral variants [8,27], and the non-Hawaiian strains, which carry more derived alleles. Among the Hawaiian strains (groups 1–14), the *sept-1/zina-1* system is the most prevalent (Fig 6B), suggesting that *sept-1/zina-1* may be the most ancestral TA system among the three. Moreover, we also observed the potential transition from *sept-1(XZ1516)* to *sept-1(N2)* in the Hawaiian strains, as three strains (ECA2949 from group 5, ECA2452 from group 13, and ECA2968 from group 14) carry both *sept-1(XZ1516)* and *sept-1(N2)* alleles. In those strains, *sept-1(N2)* remains linked to *zina-1*, while *sept-1(XZ1516)* is on a small contig that does not contain any other gene. Thus, we suspect that a duplication event created two copies of *sept-1(XZ1516)*; one copy accumulated mutations and became the non-toxic *sept-1(N2)*, while the other moved out the locus and was subsequently lost. Many Hawaiian strains carried only *sept-1(N2)* (e.g., group 12) or no elements from any of the three TA elements (e.g., group 6). The above results led to a model that *sept-1/zina-1* is an ancestral TA system that was gradually lost during the evolution of *C. elegans*. In fact, in the non-Hawaiian population (groups 15–24), not a single strain carries *sept-1/zina-1*, while many carry either *sup-35/pha-1* or *peel-1/zeel-1* or both (Fig 6B).

While inspecting the genomes of the wild isolates, we noticed a few unexpected cases where the toxin is preserved but the antidote is lost. For example, in six strains (groups 4, 10, and 17) *peel-1* can be found but *zeel-1* is pseudogenized or lost. Among the six strains, the four Hawaiian ones (ECA701 from group 4; ECA1769, ECA2091, and ECA2191 from group 10) all carry a F97Y variant in *peel-1*. So, one hypothesis is that this substitution might render PEEL-1 non-toxic. Interestingly, XZ1516 and other Hawaiian strains also carry the F97Y variant, questioning whether *peel-1* exerts toxicity in Hawaiian strains. For the non-Hawaiian JU1652 and NIC166, we found no nonsynonymous mutations in *peel-1* despite the loss of *zeel-1*. Either the antidote annotation is incorrect due to low genome quality, or an alternative detoxification mechanism exists. The same applies for the four strains (ECA2183 and ECA2960 from group 2, ECA2452 from group 13, and ECA2968 from group 14) that carry *sept-1(XZ1516)* and pseudogenized *zina-1*.

For *sup-35/pha-1*, previous work suggested that *pha-1* was deleted and *sup-35* was pseudogenized in the DL238 strain leading to the maternal-effect incompatibility with the N2 strain. Upon close inspection of the assembled genome of DL238, we found an open reading frame for *sup-35*, and the predicted SUP-35(DL238) proteins showed significant sequence divergence from the SUP-35(N2) protein (42.9% identity and 57% similarity; S8A and S8B Fig). Phylogenetic reconstruction indicated that *sup-35* is likely derived from a tandem duplication of the nearby *Y48A6C.4*, which is a paralog of the *rmd* genes (especially *rmd-1* and *rmd-2*) (S8C Fig). In strains like N2, this duplication led to the formation of the toxin SUP-35, whereas in strains like DL238, the duplicate gene had accumulated variants that render it non-toxic. In XZ1516, complex duplication and rearrangement led to three copies of *Y48A6C.4* and three copies of *sup-35*, which are all diverged from *sup-35(N2)* (S1B and S8C Figs). The above results mirror the effect of sequence divergence between SEPT-1(XZ1516) and SEPT-1(N2) and led us to hypothesize that accumulation of missense mutations in the toxin gene may be a common way to disable its toxicity while potentially maintaining its other functions.

### *sept-1/zina-1* system is a *C. elegans*-specific innovation

**Fig 7:**
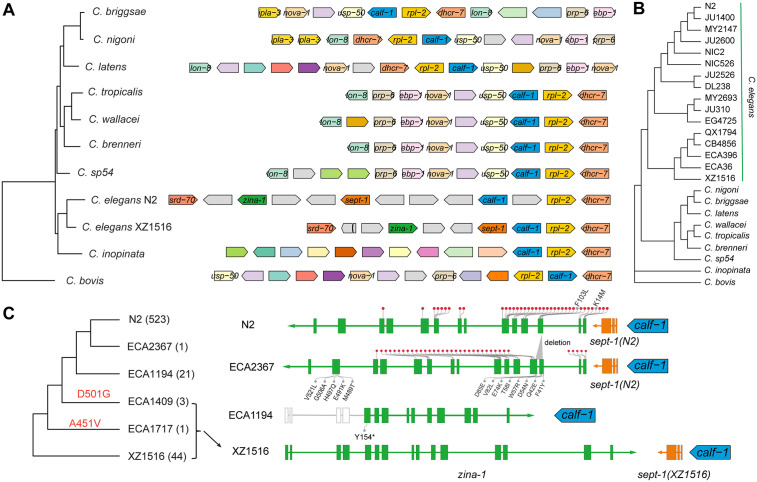
*sept-1/zina-1* is an ancestral TA system in *C. elegans.* **(A)** The genomic region homologous to the *C. elegans* region that contains *sept-1/zina-1* in selected *Caenorhabditis* species were shown. Conserved genes with public names were labeled. Despite the divergence in *C. elegans* and *C. inopinata*, all genomes contain *calf-1*, *rpl-2*, and *dhcr-7* in this region. **(B)** A phylogenetic tree built by IQ-TREE with maximum likelihood method using the concatenated *calf-1*, *rpl-2*, and *dhcr-7* genomic sequences from the selected *Caenorhabditis* species and *C. elegans* wild isolates. The genomes of the wild isolates were assembled using the long-read sequencing results. **(C)** A phylogenetic tree based on *zina-1* coding sequences. Four haplotypes were illustrated on the right; the number of strains represented by the haplotype is indicated in the paratheses. XZ1516 carries the haplotype with *sept-1(XZ1516)* and reference *zina-1*. ECA1409 and ECA1717 carry one missense mutation in *zina-1*. ECA1194 represents a haplotype in which *sept-1* is deleted. In most strains of this group, *zina-1* were pseudogenized by a premature stop (Y154*). In ECA1194, the first four exons of *zina-1* were not covered by the short reads and were thus depicted in gray. The ECA2367 and N2 haplotypes carry many missense variants in *zina-1*. The ones shared by both were shown as red dots, and the one specific to each were labeled by gray dots and spelled out. Fig 2B also showed the missense variants in *zina-1(N2*ψ).

To investigate the evolutionary origin of the *sept-1/zina-1* TA system, we searched for homologous sequences across the nematode genomes related to *C. elegans* using BLASTN and BLASTP but did not find any significant homolog for either *sept-1* or *zina-1*. We then created multispecies alignment to examine the syntenic evolution of the locus harboring *sept-1/zina-1* and found that this genomic region is conserved cross most species but shows divergence in *C. elegans* and *C. inopinata* (Fig 7A). *sept-1/zina-1* appears to be a *C. elegans*-specific genomic innovation. Despite the broken synteny, three genes (*calf-1*, *rpl-2*, and *dhcr-7*) in this region are conserved throughout all species. Using the genomic sequences of these three anchor genes, we constructed a phylogenetic tree to show the evolutionary history of this TA-bearing region. The topology of the tree places the XZ1516 haplotype next to other sister species and the N2 haplotype within the *C. elegans* lineages, suggesting that the XZ1516 haplotype represents a more ancestral state than the N2 haplotype (Fig 7B). This finding supports that *sept-1/zina-1* is an ancestral TA system that is subsequently inactivated in more derived lineages.

In addition to the N2 haplotype in which *sept-1* is diverged and *zina-1* is pseudogenized by an internal deletion, we identified two other haplotypes for the *sept-1/zina-1* locus (Fig 7C). One carries the *sept-1(N2)* allele and a functional open reading frame for *zina-1* without the deletion of exon 3 and 4. However, this *zina-1(ECA2367)* allele carries most of the missense variants found in *zina-1(N2*ψ), indicating that the accumulation of missense mutations in *zina-1* occurred before the frameshift-inducing deletion. Given that adding the deleted sequences back into *zina-1(N2*ψ) did not restore its detoxification ability (Fig 3A), we suspect that *zina-1* has already lost its function before the internal deletion that pseudogenized the gene. This is expected, since the toxin *sept-1* has already evolved to the non-toxic *sept-1(N2)* allele in this haplotype. Another haplotype (e.g., in ECA1194) showed the deletion of *sept-1*, suggesting an alternative way of inactivating the *sept-1/zina-1* TA system.

## Discussion

In this study, by crossing the laboratory N2 strain with the highly diverged Hawaiian XZ1516 strain, we identified a new TA system in XZ1516 and named it *sept-1/zina-1*. This TA system is the same as the *mll-1/smll-1* system identified by Zdraljevic and colleagues, in a recent study that crossed XZ1516 with QX1211 strain [19]. We and their studies independently mapped the TA system to the same region on the right arm of the XZ1516 genome and showed that the toxin is maternally transmitted into the oocyte likely through the deposit of toxin mRNA. We and their studies both found that the toxin has a homolog *B0250.4* or *sept-1(N2)* in the N2 strain, while the antidote is pseudogenized. However, our explanation of this unusual evolutionary pattern differs.

The study by Zdraljevic and colleagues suggests that *sept-1(N2)* is not toxic because its expression is suppressed by secondary 22G siRNAs generated by the primary piRNA targeting the *sept-1(N2)* transcripts [19]. In contrast, our work indicate that the toxicity of the SEPT-1(N2) is significantly attenuated by protein sequence divergence to the point that the presence of the antidote is no longer required for the survival of the animals in the N2 strain. Even when the small RNA-mediated suppression of the *sept-1(N2)* is removed through recoding the sequence or mutating *prg-1*, we still did not observe any rod-like arrested larva. Moreover, when expressed from the *sept-1* locus in XZ1516 background, *sept-1(N2)* did not cause any lethality either in its original or recoded sequences, supporting that the SEPT-1(N2) protein is non-toxic. Our findings suggest that even if *sept-1(N2)* is targeted by the endogenous small RNAs, the suppression of its mRNAs is not likely the major reason for the lack of toxicity in the absence of the antidote. Instead, the divergence of the actual protein sequences (only 47% identity) between SEPT-1(XZ1516) and SEPT-1(N2) counts for most of the differences in toxicity. Interestingly, the coding sequence of SUP-35 proteins in DL238, XZ1516, and other strains also differs from that of the toxic N2 form, which may explain the lack of toxicity in those strains. Nevertheless, it remains unclear whether *sup-35* evolved from a nontoxic form to a toxic form or the other way around.

In addition, the promoter of *sept-1(XZ1516)* appears to be stronger than the *sept-1(N2)* promoter, and the stem-loop structure in *sept-1(XZ1516)* 3′UTR might help promote the stability and facilitate the translation of the *sept-1(XZ1516)* mRNA, as the 3′-terminal stem-loop is known to do so [28]. On the contrary, *sept-1(N2)* does not have a similar 3′UTR. Moreover, SEPT-1(N2) proteins may be less stable than SEPT-1(XZ1516). Thus, we concluded that multiple mechanisms enabled the evolution of the *sept-1* locus from a toxic allele in XZ1516 into a non-toxic allele in N2.

The larval arrest phenotype caused by SEPT-1 toxicity is morphologically similar to a previously reported rod-like larval lethal phenotype found in mutants of Ras pathway genes [29]. However, the rod-like lethality in Ras mutants were caused by defects in the development or function of the excretory system (leading to the buildup of fluids in the body), whereas SEPT-1-induced lethality appears to be caused by defects in the intestine. Thus, different mechanisms may have led to the same rod-like phenotype.

Although the function of SEPT-1 and how it poisons the intestine are unknown, the antidote ZINA-1 has 10 clearly defined zinc finger motifs. Since ZINA-1 is not localized in the nucleus, we suspect that it may not be a DNA-binding protein and is instead an RNA-binding protein. One appealing hypothesis is that ZINA-1 may block the effects of SEPT-1 by binding to the *sept-1* mRNA. The long hairpin structure of the *sept-1* 3′UTR may be a potential binding site for ZINA-1. Nevertheless, we cannot rule out the possibility that ZINA-1 may function in an RNA-independent manner, as many zinc finger proteins do [30].

Our phylogenetic analysis uncovered an evolutionary history of the three known TA systems in *C. elegans*. We found that the *sept-1/zina-1* is the most prevalent TA in the Hawaiian strains carrying the most ancestral variants of the species. The deletion of *sept-1* or the transition from the toxic form to a non-toxic allele through accumulation of missense mutations, accompanied by *zina-1* pseudogenization, were observed in the more derived Hawaiian strains. This result suggests that *sept-1/zina-1* was already inactivated in the Hawaiian population. Coinciding with the loss of *sept-1/zina-1*, another maternal TA *sup-35/pha-1* and the paternal TA *peel-1/zeel-1* started to propagate. In fact, majority of the strains in the non-Hawaiian population carry either *sup-35/pha-1* or *peel-1/zeel-1* or both, and none of them carries active *sept-1/zina-1*. Interestingly, four Hawaiian strains (group 3 in Fig 6A) contain all three known TAs, which echoes previous findings that some *C. tropicalis* isolates could carry multiple maternal-effect TA systems [14,31]. On the other hand, many strains (both Hawaiian and non-Hawaiian) have none of the three TAs, suggesting dynamic intraspecific evolution of the TA systems.

## Materials and methods

### *C. elegans* strains

*C. elegans* strains were maintained at 20 °C on nematode growth medium (NGM) plates seeded with OP50 bacteria according to previous methods [32]. The XZ1516 strain was requested from the CaeNDR database [9]. Transgenesis was performed by injecting the recombinant DNA into the gonad of young adults, and transgenic animals were maintained by picking the transformants with fluorescent co-injection markers. For CRISPR/Cas9-mediated gene editing in *C. elegans*, the recombinant Cas9, sgRNA, and the repair template were injected together into the worm gonads, and the progeny were screened by PCR-based genotyping. Some strains were provided by the *Caenorhabditis* Genetics Center (CGC). All strains used in this study are listed in the S3 Table.

### Alignment, variant calling, genome assembly, gene annotation, and visualization

In general, the alignment of the short-read sequencing results was done using STAR (v2.7.11b) and bwa mem (v0.7.19-r1273) with default parameter for splicing and non-splicing alignment, respectively. Deepvariant (v1.5.0) were used for variant calling. Lastz (v1.04.15) and mummer (v3.0.0) were used for large scaffolds pairwise alignment. Bedtools (v2.31.1), Conda (v24.11.3), Singularity (v3.8.0), and snakemake (v7.25.4) were used to coordinate computational tasks on high-performance cluster and to ensure reproducibility. The scaffold-level genomes of 14 *C. elegans* wild isolates were downloaded from NCBI [PRJNA692613], and ragtag (v2.1.0) was used to assemble the wild isolates genomes into chromosome level with the guide of N2 reference genome (WS285). Funannotate (v1.83) was used to de novo-annotate the chromosome-level genomes. The assembled genomes were uploaded to NCBI [PRJNA1477378]. CB4856 genome was downloaded from NCBI [GCA_963921145.1 from PRJEB26645]. Blastp (v2.16.0+) was used to compare the homology of proteome between wild isolates and N2. Exonerate (v2.80.0) was used to re-annotate the conserved or pseudogenized genes in wild isolates. Visualization of the genomic data is performed in R using ggplot2 (v3.5.2), ggtree (v3.12.0), and a self-built R package ggexon (https://github.com/DongyaoLiu/ggexon). Software used in this study are listed in S4 Table. All original code has been deposited at the github repository (https://github.com/DongyaoLiu/ToxinAntidote).

### Chromosomal mapping of the TA in XZ1516

For chromosomal mapping, we used the transgenes *zdIs5[mec-4p::GFP] I*, *muIs32[mec-7p::GFP] II*, *uIs31[mec-17p::GFP] III*, *uIs115[mec-17p::TagRFP] IV*, *uIs134[mec-17p::TagRFP] V*, and *uIs130[lad-2p::GFP] X* as the chromosomal markers for N2 and counted the percentages of live and arrested animals carrying the fluorescent marker among the offspring of the N2/XZ1516 hybrid. At least three biological replicates were carried out, and more than 500 F2 progeny were examined for each marker.

### Introgression and the mapping of the TA elements

To introgress the XZ1516-derived TA system into the N2 strain, we crossed N2 males with XZ1516 hermaphrodites and picked N2/XZ1516 hermaphrodites to cross them with N2 males again. We then repeated the cross for seven rounds and obtained the near isogenic line named CGZ1589. To make sure CGZ1589 carries the TA system, we crossed it with N2 and found rod-like arrested animals among the progeny of N2/CZ1589 hybrids. We then extracted genomic DNA from CGZ1589 using the Puregene tissue core Kit (Qiagen, cat# 1126829). Ten micrograms of the DNA was used for library construction and sequencing by BGI (Beijing Genome Institute, China) on the DNBSEQ PE150 platform. The sequencing depth is ~100×. Raw sequencing file of the introgressed strain is uploaded to NCBI Sequence Read Archive (SRA) under the accession number PRJNA1359096.

To generate high-quality genomic variants between XZ1516 and N2. We use ART (v2016-06-05) to simulate 200 bp pair-end reads from N2 and XZ1516 (with the 50× depth) and map the simulated reads to each other’s genome (we used the reference genome WS285 for N2 and assembled the XZ1516 genome from long-read sequencing results). We used deepvariant (v1.5.0) to identify N2 variants based on the XZ1516 genome and XZ1516 variants based on the N2 genome and then intersected the two lists of variants to identify the final high-quality variants. We then mapped the sequencing reads of the introgressed strain CGZ1589 to the N2 reference genome and counted the reads that support either the N2 variants or XZ1516 variants (at the high-quality variant sites with alternative alleles) across the genome. The read depth of different alleles in CGZ1589 were quantified using bam-readcount (v1.0.1). The region that contains most reads supporting the XZ1516 variants was identified as the TA-containing region (17–22 Mb on the right arm of chrV).

We then filtered the genes annotated in this region of the XZ1516 genome for gene size (genes coding proteins longer than 100 amino acids), expression levels (with supporting RNA-seq reads), and the level of homology with N2 genes (lower than 80% identity) and obtained a list of genes for RNAi screen. To include the possibility that the TA system may be located on the scaffolds that were not assembled into the chromosome-level XZ1516 genome, we also used Augustus (v3.5.0) to predict the genes located on any scaffolds aligned to N2 chrV and identified genes with the support of RNA-seq reads (PRJNA669810).

### RNAi screen for the antidote gene

Feeding RNAi was carried out using a previously described protocol [33]. The cDNAs of the 46 candidates for the antidote gene were cloned from the cDNA library of XZ1516 and inserted between the two T7 promoters in the L4440 vector using Gibson Assembly (primers used for the cloning were listed in S1 Table). The resulted DNA constructs were transformed into *E. coli* HT115 strain. The bacteria containing the RNAi constructs were seeded onto NGM plates with 5 mM IPTG to induce the expression of dsRNAs. The next day, 30 bleach-synchronized embryos were placed onto the RNAi plates. The next generation were examined for the presence of rod-like arrested larvae.

### Genotyping for the inheritance pattern of the *sept-1/zina-1* locus

Live F2 progeny generated by various F1 hybrids were subjected to single-worm PCR. For the experiments that involved extrachromosomal arrays expressing the antidote, we only genotyped F2 animals that were alive and carried the array. At least three replicates were carried out, and more than 96 animals were genotyped for each cross. The genotyping primers were listed in S5 Table.

### DNA constructs and expression reporters

We performed Gibson Assembly for cloning in this study using ClonExpress II One Step Cloning Kit (cat# C112-01) from Vazyme (Nanjing, China). To generate the transcriptional reporters, a 2-kb *sept-1(XZ1516)* promoter and a 2-kb *sept-1(N2)* promoter were cloned from the genomic DNA of XZ1516 and N2 strains, respectively, and then ligated upstream of TagRFP and a *tbb-2* 3′UTR in the pUC57 backbone. For the *zina-1* rescue experiment, *zina-1* fragment containing a 2-kb *zina-1* promoter, the entire coding region, and a 1-kb *zina-1* 3′UTR was cloned from XZ1516 genomic DNA. We then swapped out the *zina-1* promoter with a 2-kb promoter upstream of the *zina-1(N2*ψ), a 2-kb *ges-1* promoter, or a 2-kb *myo-2* promoter from the N2 genomic DNA. To test the detoxification ability of zina-1(N2*), which is the repaired *zina-1* allele in N2, we cloned the *zina-1* coding sequence from the cDNA library of animals carrying the *zina-1(unk232)* allele or substituted the exon 3 of the *zina-1(N2*ψ) cloned from N2 cDNA library with the exon3 and exon4 of *zina-1* from XZ1516 cDNA library. This zina-1(N2*) fragment was then placed downstream of either *zina-1* promoter or the *zina-1(N2*ψ) promoter. To make *zina-1p::GFP* transcriptional reporters, a 2-kb *zina-1* promoter and a 1-kb *zina-1* 3′UTR were cloned from the XZ1516 genomic DNA and were ligated to the upstream and downstream of GFP coding sequences, respectively. To make *zina-1* translational reporter, GFP coding sequence is inserted upstream of the *zina-1* coding sequence in the *zina-1* fragment containing the promoter, the coding region, and the 3′UTR cloned from the XZ1516 genomic DNA. All DNA constructs generated and used in this study are listed in S6 Table.

### MosTI single-copy insertion

We used the previously described modular safe-harbor transgene insertion (MosTI) method for the insertion of single-copy transgenes [20]. Briefly, we cloned the genomic DNA of *sept-1(XZ1516)* and *sept-1(N2)* and ligated it to the downstream of the *hsp-16.41* promoter and the upstream of the *tbb-2* 3′UTR on the pSEM246 backbone through Gibson Assembly. The resulted constructs were injected into the CFJ42 strain, which carries a landing site (*kstSi42*) on chromosome II and the *unc-119(ed3)* mutation. Successful integrants were selected based on normal locomotion and were confirmed through genotyping and sequencing. We obtained and tested multiple lines from each transgenesis. The results of the following lines were shown: *unkSi32* and *unkSi18* expressed *sept-1(XZ1516)* and *sept-1(N2)*, respectively, from the heat-shock promoter, while *unkSi42* and *unkSi46* expressed 2xHA-tagged SEPT-1(XZ1516) and SEPT-1(N2), respectively, from the heat-shock promoter.

### CRISPR/Cas9-mediated gene editing

Gene editing was conducted using our previously published protocol [34]. Cas9 cut sites were predicted using CHOPCHOP (https://chopchop.cbu.uib.no). Single-guide RNAs were prepared by in vitro transcription using the NEB sgRNA synthesis kit (E3322S) and were incubated with recombinant SpCas9 protein (NEB cat# M0646T) at 37 °C for 15 min. The RNP (2 μM Cas9 and 100 ng/μL of guide RNA in final concentration) was then mixed with a linearized muscle-expressing co-injection marker construct (CGZ#400, *ttr-16p::GFP* at 10 ng/μL), repair template (at 100 ng/μL if needed), and filler DNA (1 kb plus DNA ladder, Invitrogen cat#10787018). For insertion or substitution longer than 150 bp, we used a previously described PCR method to generate repair template with overhangs [35]. For edits shorter than 150 bp, single-stranded donor oligos were used as repair template. After the injection, transformants with GFP expression in the body wall muscle were picked to individual plates and genotyped by PCR and Sanger sequencing to find the correctly edited allele. In some cases, we also used a co-CRISPR protocol [36] to screen for the successfully edited animals with *dpy-10(cn64)* as the co-conversion marker. The sequences of the CRISPR/Cas9 target and repair templates used to generate the edited alleles were listed in S7 Table.

### smFISH, antibody staining, and microscopy imaging

smFISH probes against the *sept-1(XZ1516)* coding region were designed using the Stellaris RNA FISH Probe Designer tool from Biosearch Technologies (Petaluma, CA) and synthesized with Quasar 670 dye. The sequence of the probes can be found in S8 Table. Dissected adult gonads and early embryos from various strains were fixed, stained with the FISH probe sets (125 nM as the final concentration) in the hybridization buffer (purchased from Biosearch Technologies), and then washed using the wash buffer based on a previous protocol [37]. For antibody staining, we used the Ruvkun protocol [38]. Early embryos were fixed in the fixation buffer and underwent three rounds of freeze-thaw cycles. Anti-HA mouse monoclonal antibody (TransGene, HT301-02) and anti-mouse secondary antibodies (Thermo-Fisher, cat# 31430) were used at 1:1,000 dilution; animals were incubated with the antibodies in the antibody buffer A (1× PBS, 1% BSA, 0.5% Triton X-100, 1 mM EDTA). Leica fluorescent microscopy (DMi8 with K5 monochrome camera) was used to image the larvae and adults. Embryos and dissected gonads were imaged on Confocal Laser Scanning Microscope (Carl Zeiss LSM 980).

### RNA sequencing and mapping of A-to-I editing

Mixed-stage worms were collected from three 100 mm NGM plates. Two biological replicates were prepared for each strain. Total RNAs were extracted using TRIzol reagent (Thermo Fisher). One milligram of total RNA were used for beads-based mRNA capturing and library construction and were sequenced on DNBSEQ-T7 by BGI. 6G adaptor-trimmed reads were used for downstream analysis. All raw RNA-seq data were uploaded to NCBI SRA under the accession number PRJNA1359096. Mapping followed the protocol mentioned above. The A-to-I mutation were identified by bcftools (v1.21).

### Phylogenetic tree and mapping TA systems in wild isolates

The phylogenetic tree for the wild isolates was calculated by iqtree(v1.5.5) using concatenated CDS of 4,736 single-copy gene defined by previous studies [26]. The CDS variations were extracted from vcf file and install to the reference transcripts using a modified script (https://github.com/DongyaoLiu/vcf2fasta). Genomic data of *C. elegans* wild isotypes and the corresponding hard-filtered vcf file were downloaded from CaeNDR (v20231213). Fastq files were extracted from the bam alignment file. We used two independent approaches to map *peel-1*/*zeel-1*, *sup-35*/*pha-1*, and *sept-1/zina-1* in *C. elegans* wild isolates.

First, we extracted all reads mapped to each corresponding locus in the N2 reference genome (WS285) and unmapped reads using samtools (v1.16.1) from the bam files and performed local de novo assembly using SPAdes (v3.15.4). The resulting scaffolds were used to annotate each TA system using Exonerate (v2.2.0). If the re-annotation produced a hit with protein identity to the query ≥80% and annotated protein length ≥80% of the reference protein length, we considered the gene “present”. If the de novo assembly existed but no hit met the above threshold, we considered the gene “absent”. Thus, for *sup-35*, genotypes matching *sup-35(DL238)* or *sup-35(XZ1516)* were significantly diverged from *sup-35(N2)* and were considered *sup-35(-)*. If nonsense or frameshift mutation were detected, we also considered the gene “absent”. If no assembly was available, we considered the case to be “N.D.” for “not determined”.

In parallel, we conducted conventional short-read mapping against the reference genome followed by variant calling using ANNOVAR. The gene was considered “present” if all CDS intervals for the gene had coverage_fraction_d10 = 1.0 (100% of CDS bases covered at ≥10× read depth) using samtools depth. For *peel-1*, *zeel-1*, *sup-35*, *pha-1*, and *sept-1(N2)*, we used the N2 (WS285) CDS coordinates. For *sept-1(XZ1516)* and *zina-1*, we used XZ1516 genomic CDS coordinates. The gene was considered “absent” if any CDS interval failed the above criteria. If nonsense or frameshift mutation were detected after the BAM file passed the depth check, we considered the gene “absent”. We considered the case to be “N.D.” if no BAM file exists for the strain.

The results of the above two approaches were then combined. A gene was called “present” if either approach supports its presence and no nonsense or frameshift mutation were found. A gene was called “absent” if neither approach supports its presence. A gene was called “N.D.” if both approaches resulted in “N.D.” In addition, we visually examined the read depth coverage of the antidote genes when the toxin was present but the antidote was missing to make sure the results of the genotype calling is correct.

To visualize the genomic region harboring the *sept-1/zina-1* system in other *Caenorhabditis* species, we selected nine *Caenorhabditis* species relative to *C. elegans* and analyzed their genomes, including *C. briggsae* (GCA_000004555.3), *C. tropicalis* (GCA_016735795.1), *C. nigoni* (GCA_002742825.1), *C. latens* (GCA_002742825.1), *C. wallacei* (GCA_963932035.1), *C. brenneri* (GCA_964036135.1), *C. sp54* (GCA_965234135.1), *C. innopinata* (GCA_003052745.1), and *C. bovis* (GCA_902829315.1). The genomic sequences of *rpl-2*, *calf-1*, and *dhcr-7* from these genomes and the genomes of the wild isolates were used to construct a phylogenetic tree.

### Statistical analysis

All quantitative data were presented as mean ± SD. At least three biological replicates were performed. For categorical data (percentage of animals carrying the marker or being alive), we used Chi-squared test to detect the statistically significant deviation from the expected frequencies. The *p* values were corrected using Bonferroni correction for multiple comparisons in one experiment. To compare two curves, we used the non-parametric Kolmogorov–Smirnov (KS) test. Detailed statistical information can be found in the figure legends.

## Supporting information

S1 Fig *sup-35/pha-1* is inactivated in XZ1516. **(A)** A comparison of the *zeel-1/peel-1* locus between XZ1516 and N2 shows good conservation of synteny (gray lines). **(B)** A comparison of the *sup-35/pha-1* locus between XZ1516 and N2 shows several genomic rearrangement events. *pha-1* is split into two short ORFs (*pha-1*^*#1*^ and *pha-1*^*#2*^) due to a deletion event in XZ1516. This split likely inactivated the *pha-1* gene function. *sup-35* is duplicated into *sup-35*^*#1*^, *sup-35*^*#2*^, and *sup-35*^*#3*^ due to duplication and inversion events but all three copies have shown significant sequence divergence from *sup-35(N2)*, which likely leads to the loss of toxicity (see S8 Fig for details). *sup-35* also shows similarity to *Y48A6C.4*, which is duplicated and split into three copies in XZ1516. **(C)** The *sup-35(unk210)* allele in N2 strain created in this study through CRISPR/Cas9-mediated gene editing. Premature stops were inserted into the first exon to inactivate the gene. **(D)** Cross schemes used to test the inheritance pattern of the chrV-linked novel TA in XZ1516. The black bars indicate the N2 chrV carrying a TagRFP-expressing fluorescent reporter (pink). The brown bars indicate the homologous XZ1516 chrV carrying a novel TA system. The results of the crosses indicate the presence of maternal deposit of the XZ1516-derived toxin. Among the alive progeny, we only scored the males, which are for sure the cross-progeny. **(E)** The result of the cross indicates the lack of paternal deposit of the XZ1516-derived toxin.
(TIF)

S2 Fig Identification of the *sept-1* and *zina-1* as the toxin and antidote genes. **(A)** XZ1516 animals treated with bacteria expressing dsRNA against *zina-1* or carrying the empty RNAi vector L4440. Scale bars, 20 μm. **(B)** Gene structures of *sept-1(XZ1516)* long and short isoforms and the molecular lesions of the various alleles. **(C)** The count of reads that align to the region that is specific for the long isoform and the region shared by long and short isoforms. **(D)** The molecular lesion of the *zina-1(unk221)* null allele. Premature stops (red) were inserted into exon 3 using CRISPR/Cas9-mediated gene editing. The data underlying this Figure can be found in S1 Data.
(TIF)

S3 Fig *sept-1/zina-1* is a maternally inherited TA system. **(A)** Cross schemes and the results of genotyping the F2 progeny. XZ1516 *sept-1(unk192)* mutants were used for “*T-*” and *sept-1(unk192) zina-1(unk221)* mutants were used as “*TA-*”. The introgressed strain CGZ1589 was used as N2 (*TA*). The toxin *sept-1* was genotyped, and its presence in the genome of the F2 progeny was considered *T+*. **(B)** Two crosses that confirmed the maternal deposition of the SEPT-1(XZ1516) toxin by genotyping the offspring. In the left cross, 100% of the progeny were alive and ~50% carry the *T+* genotype. In the right cross, only ~50% of the progeny were alive and they all carry the *T+* genotype. Some images were created using Biorender. The data underlying this Figure can be found in S1 Data. Part of the Figure was created in BioRender. Zheng, C. (2026) https://BioRender.com/5fjhayv.
(TIF)

S4 Fig Genomic editing of the *sept-1/zina-1* locus. **(A)** The *zina-1(unk232)* allele denotated as zina-1(N2*) was constructed by inserting 190-bp sequence into the exon 3 of *zina-1(N2ψ)*. These sequences were taken from the exon 3 and exon 4 of *zina-1(XZ1516)*. **(B)** The schematic presentation of the *unkSi32[hsp16.41::sept-1(XZ1516)::tbb-2 3′UTR]*, *unkSi18[hsp16.41::sept-1(N2)::tbb-2 3′UTR]*, *unkSi42[hsp-16.41::sept-1(XZ1516)::2xHA::tbb-2_3UTR]*, and *unkSi46[hsp-16.41::sept-1(N2)::2xHA::tbb-2_3UTR]*, which are single-copy insertions into the chrII: *kstSi42* locus of the CFJ42 strain (N2 background) using the MosTI method. **(C)** Editing of the endogenous *sept-1* locus in XZ1516 and N2. The coding sequence was replaced by the desired sequence using CRISPR/Cas9 gene editing. **(D)** The expression of *sept-1* mRNA detected by RT-PCR in various animals. *ama-1* served as an internal control.
(TIF)

S5 Fig Induced expression of *sept-1(XZ1516)* generates stronger toxicity than *sept-1(N2).* **(A)** RT-qPCR results showed the fold change of the induced expression of *sept-1(XZ1516)* and *sept-1(N2)* from the single-copy transgenes *unkSi32 and unkSi18*, respectively, at 2 hours after the 33 °C heat-shock of various duration. Fold change was calculated in comparison with the animals that did not undergo heat-shock. **(B)** Western blot results using anti-HA antibodies showed the expression of SEPT-1(XZ1516)::HA and SEPT-1(N2)::HA proteins from the *unkSi42* and *unkSi46* transgene, respectively, at 2 or 4 hours after a 30-min or 60-min heat-shock. Tubulin is used as a loading control. **(C)** Percentage of animals that survived 24 hours after the heat-shock at L1 or L4 stage. Three to five replicates were made for each condition with ~100 animals analyzed. Double asterisks indicate *p* < 0.01 in a Tukey’s test comparing the transgenic animals with the wild type or between the two transgenes (*unkSi32 and unkSi18* were used). **(D)** L4-stage animals were heat-shocked at 33 °C for 60 min and then imaged at 24 hours after the heat-shock. Scale bars, 10 μm. The dorsoventral width (indicated by the lines) of the intestine was measured and normalized to the width of the worm body. Around 40 animals were analyzed. The data underlying this Figure can be found in S1 Data.
(TIF)

S6 Fig Somatic expression of the *sept-1(XZ1516)* and *sept-1(N2)* genes. Expression patterns of *sept-1(XZ1516)p::TagRFP* and *sept-1(N2)p::TagRFP* in the XZ1516 **(A)** and N2 backgrounds **(B)**. The DNA constructs were injected into the two strains at the same concentrations, and the stable lines were imaged at different stages. Quantifications of the fluorescent intensity are shown in the bar graphs on the right. Expression of *sept-1(XZ1516)p::TagRFP* was stronger than *sept-1(N2)p::TagRFP* in both backgrounds. Scale bars, 20 μm. The data underlying this Figure can be found in S1 Data.
(TIF)

S7 Fig Distribution of the three TA systems in *C. elegans* wild isolates. A phylogenetic tree of 611 *C. elegans* wild isotypes constructed using optimal Bayesian models selected by ModelFinder based on the CDS of 4,736 single-copy genes. The presence and absence of the toxin and antidote genes were called using the criteria listed in the Materials and methods. Strains that were isolated from Hawaiian Islands are labeled in orange. The group each strain is assigned to is also shown. The tree file underlying this Figure can be found in S1 Data.
(TIF)

S8 Fig Divergence of *sup-35* among the *C. elegans* wild isolates. **(A)** A comparison of the *sup-35/pha-1* locus between DL238 and N2 shows the deletion of the *pha-1* gene in DL238. An inversion event caused divergence of *sup-35*. However, unlike previously reported, *sup-35* is not pseudogenized in DL238. An ORF can be constructed from the *sup-35* gene in DL238. **(B)** Pairwise alignment of SUP-35(N2) and SUP-35(DL238). **(C)** A phylogenetic tree of *rmd-1/2*, *sup-35*, and *Y48A6C.4* homologs from various *C. elegans* wild isolates and other nematodes. The location of *sup-35*^*#1~3*^ and *Y48A6C.4*^*#1~3*^ in XZ1516 genome can be found in S1 Fig.
(TIF)

S1 Table Candidate genes for the antidote that were tested by RNAi. Column A to E show the names and coordinates of the genes in XZ1516 genome. Column F shows the transcript name of the N2 gene aligned to the XZ1516 gene. Columns G to O show the alignment details with the XZ1516 gene as the query (e.g., Qstart and Qend) and the N2 gene as the target sequence (e.g., Sstart and Send). The percentage of identity for the alignment is shown in column O. Column P shows the RNA expression level of the candidate gene in XZ1516. Column S shows whether the RNAi of the gene induced the rod-like phenotype. Columns T and U list the primers used to amplify the target XZ1516 gene, and the amplified DNA fragments were inserted into L4440 for feeding RNAi.
(XLSX)

S2 Table Distribution of the three TA systems in *C. elegans* wild isolates. Detailed strain information for Figs 6A and S7. The number of strains and the number of Hawaiian strains in the group were shown. Groups 1–14 are Hawaiian groups because ≥ 50% of the strains are Hawaiian strains. Groups 15–24 are assigned as non-Hawaiian groups. The number of minority strains (i.e., non-Hawaiian strains in a Hawaiian group or Hawaiian strains in a non-Hawaiian group) is shown in the “Minority” column. The calling of the genotypes is described in Materials and methods. “N.D.” means the genotypes of the TA systems are unknown.
(XLSX)

S3 Table A list of strains used in this study. (XLSX)

S4 Table A list of software used in this study. (XLSX)

S5 Table A list of primers used in this study. (XLSX)

S6 Table A list of DNA constructs used in this study. (XLSX)

S7 Table Details of the CRISPR alleles generated in this study. (XLSX)

S8 Table The sequence of the smFISH probes against *sept-1.* (XLSX)

S1 Data Numeric data underlying all main and supporting figures. (XLSX)

S1 Raw Images Original gel and blot images for S4D and S5B Figs. (PDF)

## Abbreviations

NILs: near-isogenic lines
smFISH: single-molecule fluorescent in situ hybridization
SRA: Sequence Read Archive
TA: toxin-antidote
