# Supplementary figures and images for: sept-1/zina-1 is an ancient toxin-antidote system in Caenorhabditis elegans
Source: PLoS Biol. 2026 Jul 23;24(7):e3003563. doi: 10.1371/journal.pbio.3003563 (PMC13411925; doi:10.1371/journal.pbio.3003563)

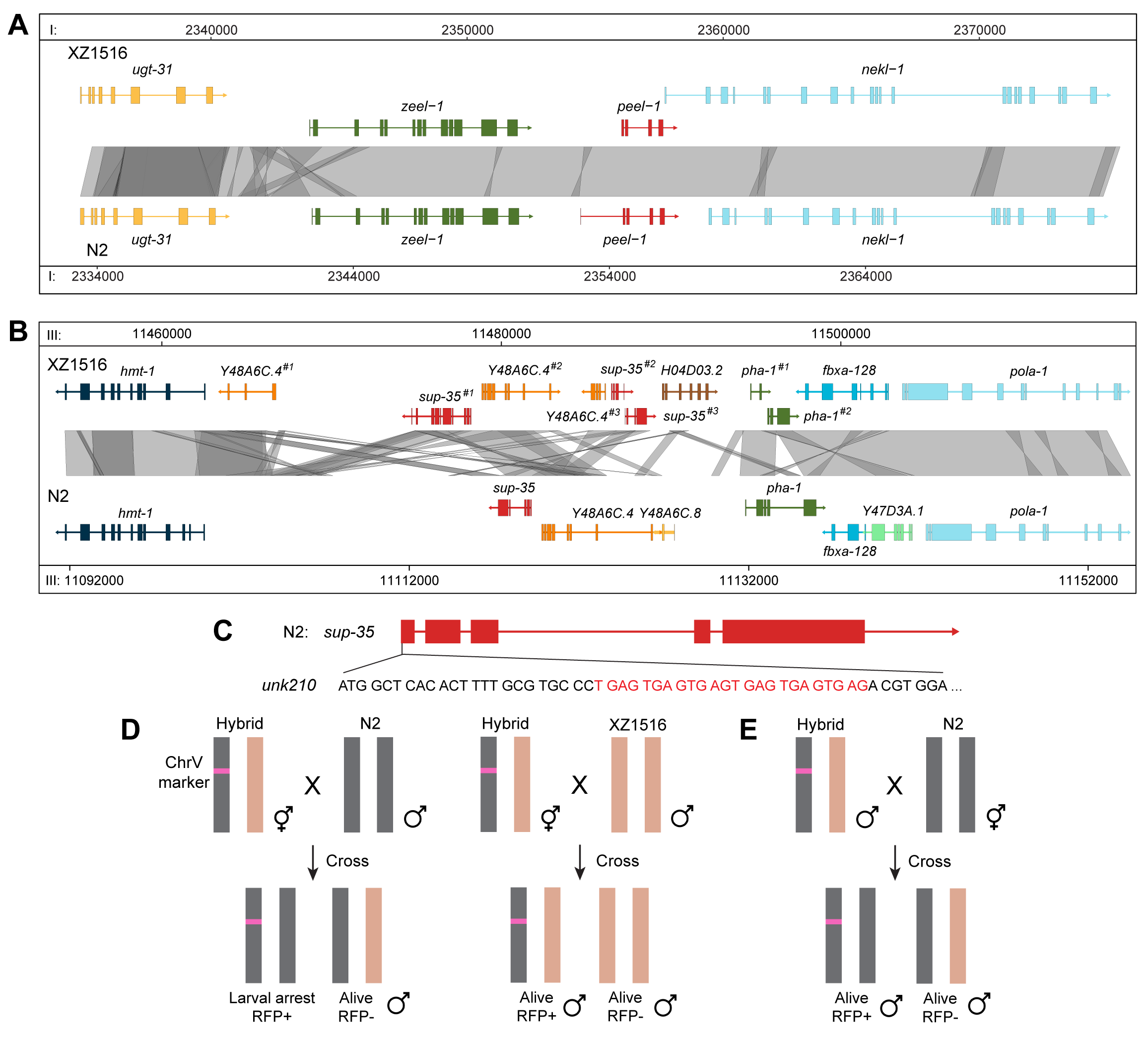

Supplement: S1 Fig — (A) A comparison of the zeel-1/peel-1 locus between XZ1516 and N2 shows good conservation of synteny (gray lines). (B) A comparison of the sup-35/pha-1 locus between XZ1516 and N2 shows several genomic rearrangement events. pha-1 is split into two short ORFs (pha-1#1 and pha-1#2) due to a deletion event in XZ1516. This split likely inactivated the pha-1 gene function. sup-35 is duplicated into sup-35#1, sup-35#2, and sup-35#3 due to duplication and inversion events but all three copies have shown significant sequence divergence from sup-35(N2), which likely leads to the loss of toxicity (see S8 Fig for details). sup-35 also shows similarity to Y48A6C.4, which is duplicated and split into three copies in XZ1516. (C) The sup-35(unk210) allele in N2 strain created in this study through CRISPR/Cas9-mediated gene editing. Premature stops were inserted into the first exon to inactivate the gene. (D) Cross schemes used to test the inheritance pattern of the chrV-linked novel TA in XZ1516. The black bars indicate the N2 chrV carrying a TagRFP-expressing fluorescent reporter (pink). The brown bars indicate the homologous XZ1516 chrV carrying a novel TA system. The results of the crosses indicate the presence of maternal deposit of the XZ1516-derived toxin. Among the alive progeny, we only scored the males, which are for sure the cross-progeny. (E) The result of the cross indicates the lack of paternal deposit of the XZ1516-derived toxin. (TIF) [file pbio.3003563.s001.tif]

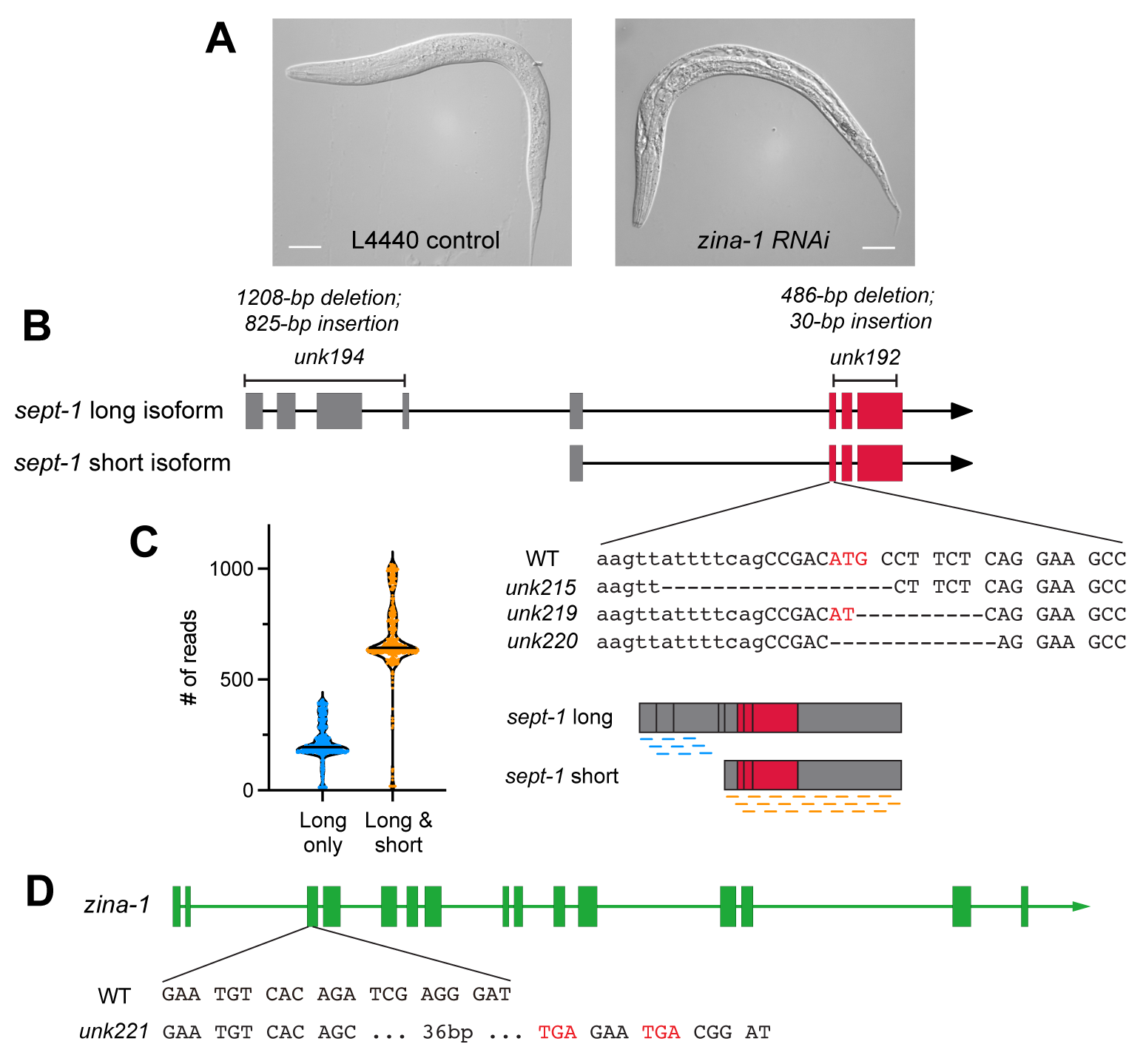

Supplement: S2 Fig — (A) XZ1516 animals treated with bacteria expressing dsRNA against zina-1 or carrying the empty RNAi vector L4440. Scale bars, 20 μm. (B) Gene structures of sept-1(XZ1516) long and short isoforms and the molecular lesions of the various alleles. (C) The count of reads that align to the region that is specific for the long isoform and the region shared by long and short isoforms. (D) The molecular lesion of the zina-1(unk221) null allele. Premature stops (red) were inserted into exon 3 using CRISPR/Cas9-mediated gene editing. The data underlying this Figure can be found in S1 Data. (TIF) [file pbio.3003563.s002.tif]

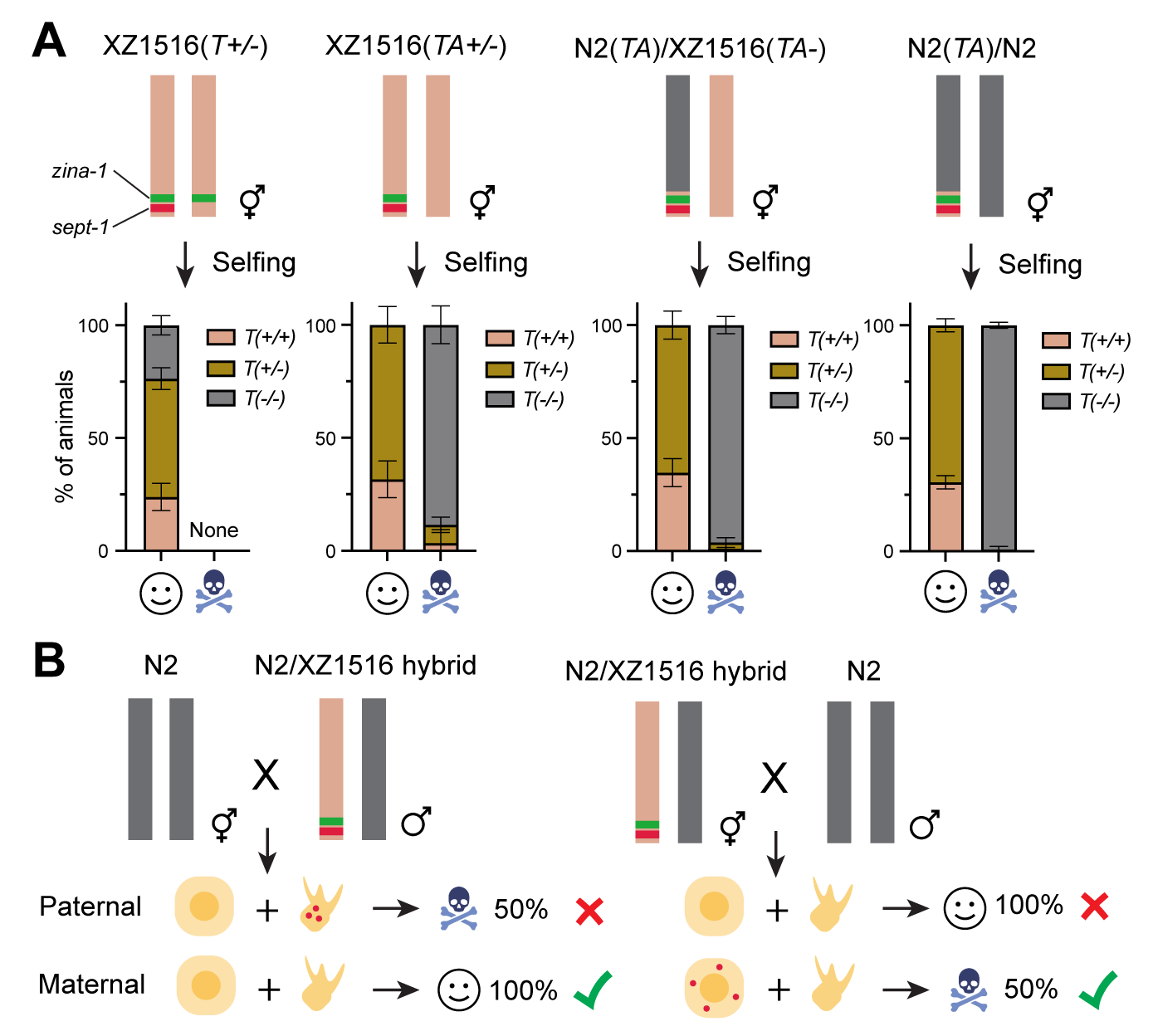

Supplement: S3 Fig — (A) Cross schemes and the results of genotyping the F2 progeny. XZ1516 sept-1(unk192) mutants were used for “T-” and sept-1(unk192) zina-1(unk221) mutants were used as “TA-”. The introgressed strain CGZ1589 was used as N2 (TA). The toxin sept-1 was genotyped, and its presence in the genome of the F2 progeny was considered T+. (B) Two crosses that confirmed the maternal deposition of the SEPT-1(XZ1516) toxin by genotyping the offspring. In the left cross, 100% of the progeny were alive and ~50% carry the T+ genotype. In the right cross, only ~50% of the progeny were alive and they all carry the T+ genotype. Some images were created using Biorender. The data underlying this Figure can be found in S1 Data. Part of the Figure was created in BioRender. Zheng, C. (2026) https://BioRender.com/5fjhayv. (TIF) [file pbio.3003563.s003.tif]

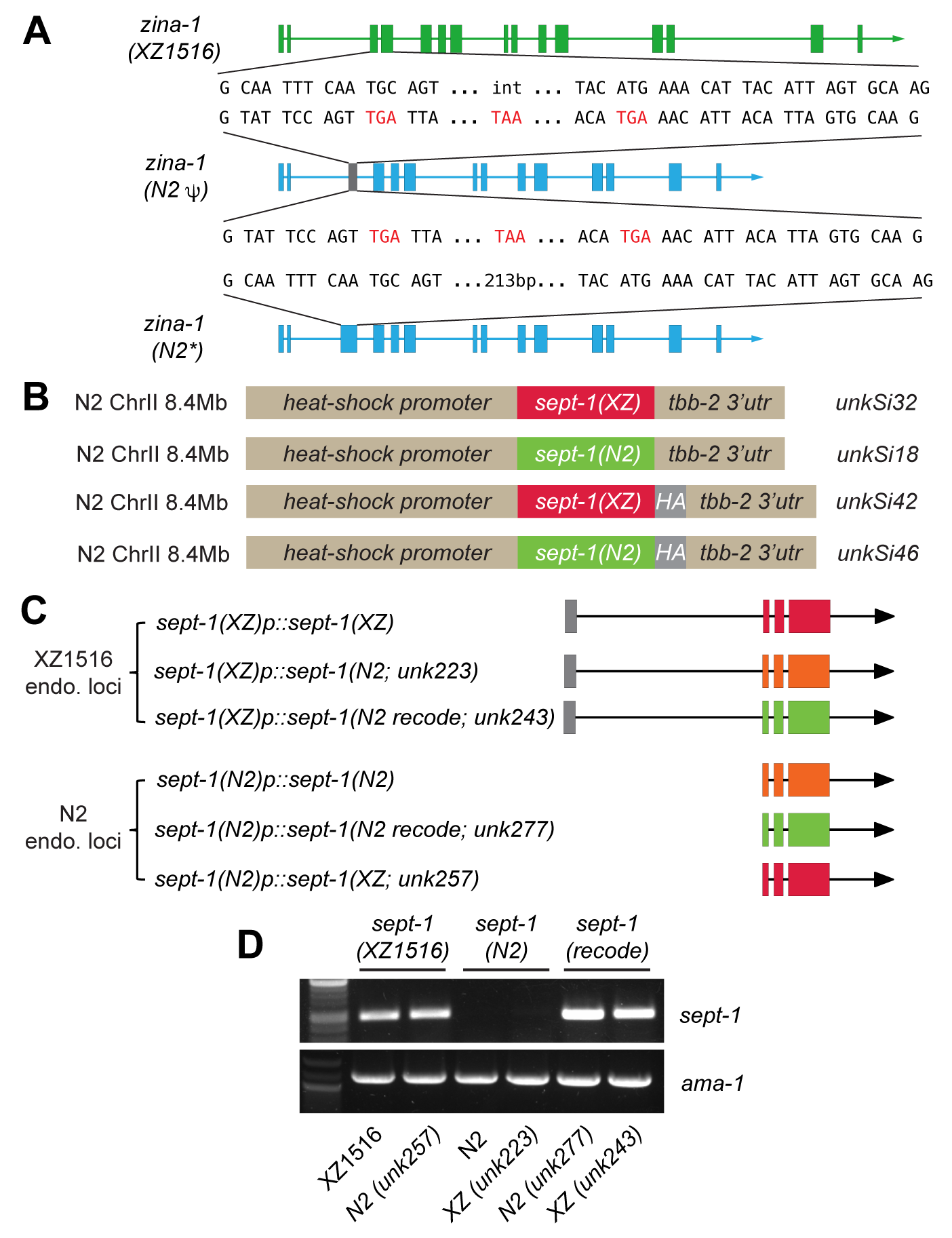

Supplement: S4 Fig — (A) The zina-1(unk232) allele denotated as zina-1(N2*) was constructed by inserting 190-bp sequence into the exon 3 of zina-1(N2ψ). These sequences were taken from the exon 3 and exon 4 of zina-1(XZ1516). (B) The schematic presentation of the unkSi32[hsp16.41::sept-1(XZ1516)::tbb-2 3′UTR], unkSi18[hsp16.41::sept-1(N2)::tbb-2 3′UTR], unkSi42[hsp-16.41::sept-1(XZ1516)::2xHA::tbb-2_3UTR], and unkSi46[hsp-16.41::sept-1(N2)::2xHA::tbb-2_3UTR], which are single-copy insertions into the chrII: kstSi42 locus of the CFJ42 strain (N2 background) using the MosTI method. (C) Editing of the endogenous sept-1 locus in XZ1516 and N2. The coding sequence was replaced by the desired sequence using CRISPR/Cas9 gene editing. (D) The expression of sept-1 mRNA detected by RT-PCR in various animals. ama-1 served as an internal control. (TIF) [file pbio.3003563.s004.tif]

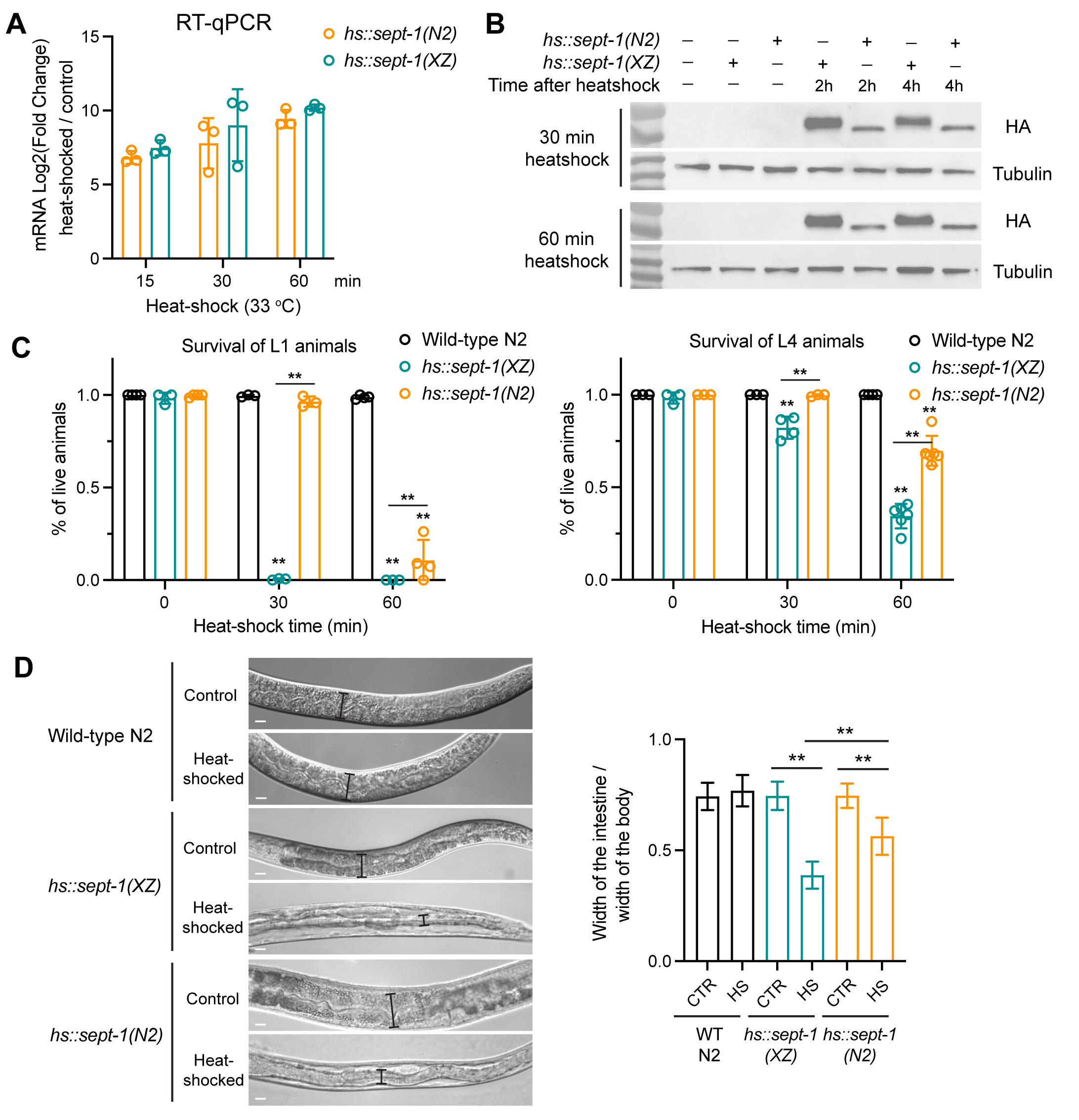

Supplement: S5 Fig — (A) RT-qPCR results showed the fold change of the induced expression of sept-1(XZ1516) and sept-1(N2) from the single-copy transgenes unkSi32 and unkSi18, respectively, at 2 hours after the 33 °C heat-shock of various duration. Fold change was calculated in comparison with the animals that did not undergo heat-shock. (B) Western blot results using anti-HA antibodies showed the expression of SEPT-1(XZ1516)::HA and SEPT-1(N2)::HA proteins from the unkSi42 and unkSi46 transgene, respectively, at 2 or 4 hours after a 30-min or 60-min heat-shock. Tubulin is used as a loading control. (C) Percentage of animals that survived 24 hours after the heat-shock at L1 or L4 stage. Three to five replicates were made for each condition with ~100 animals analyzed. Double asterisks indicate p < 0.01 in a Tukey’s test comparing the transgenic animals with the wild type or between the two transgenes (unkSi32 and unkSi18 were used). (D) L4-stage animals were heat-shocked at 33 °C for 60 min and then imaged at 24 hours after the heat-shock. Scale bars, 10 μm. The dorsoventral width (indicated by the lines) of the intestine was measured and normalized to the width of the worm body. Around 40 animals were analyzed. The data underlying this Figure can be found in S1 Data. (TIF) [file pbio.3003563.s005.tif]

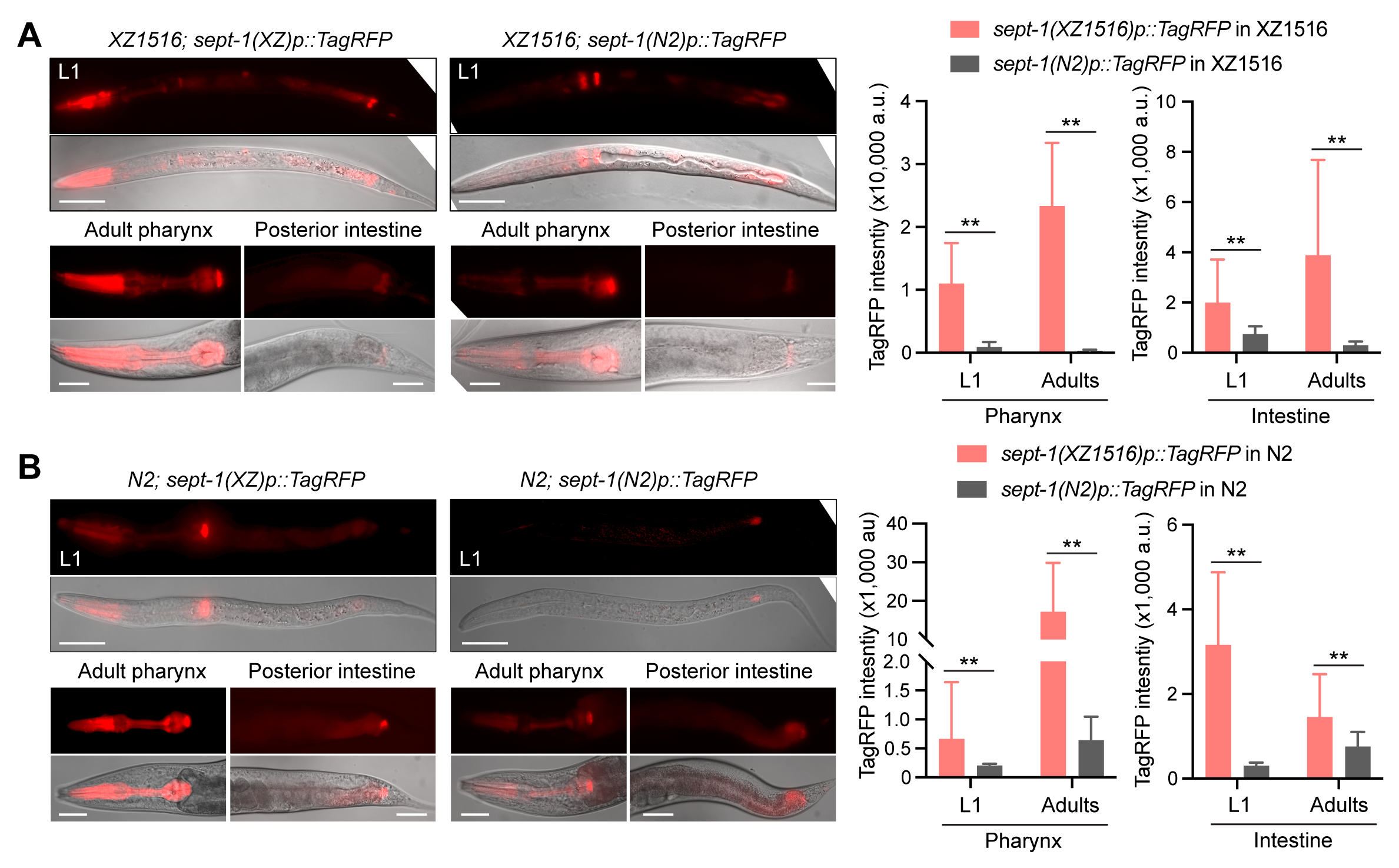

Supplement: S6 Fig — Expression patterns of sept-1(XZ1516)p::TagRFP and sept-1(N2)p::TagRFP in the XZ1516 (A) and N2 backgrounds (B). The DNA constructs were injected into the two strains at the same concentrations, and the stable lines were imaged at different stages. Quantifications of the fluorescent intensity are shown in the bar graphs on the right. Expression of sept-1(XZ1516)p::TagRFP was stronger than sept-1(N2)p::TagRFP in both backgrounds. Scale bars, 20 μm. The data underlying this Figure can be found in S1 Data. (TIF) [file pbio.3003563.s006.tif]

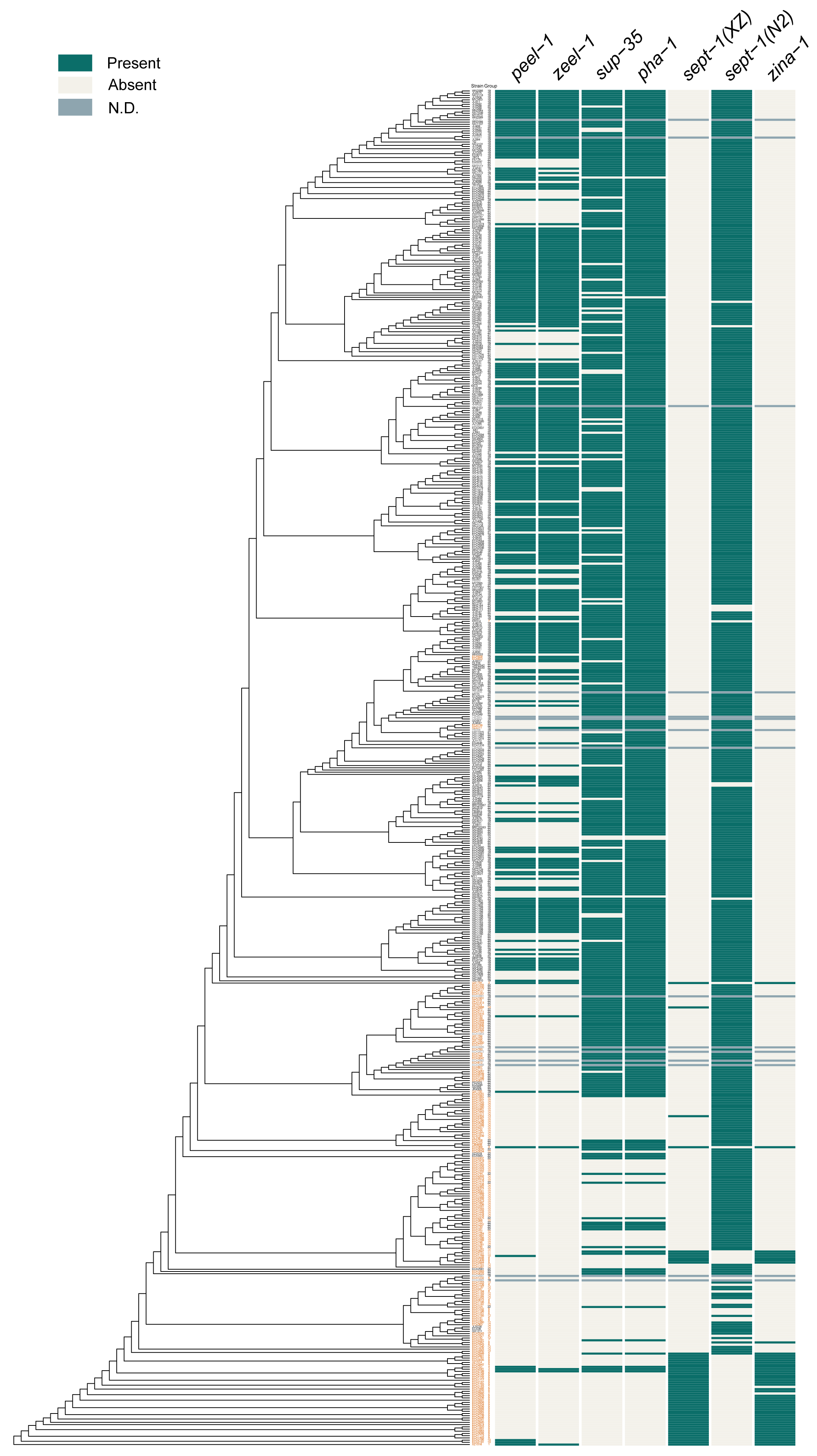

Supplement: S7 Fig — A phylogenetic tree of 611 C. elegans wild isotypes constructed using optimal Bayesian models selected by ModelFinder based on the CDS of 4,736 single-copy genes. The presence and absence of the toxin and antidote genes were called using the criteria listed in the Materials and methods. Strains that were isolated from Hawaiian Islands are labeled in orange. The group each strain is assigned to is also shown. The tree file underlying this Figure can be found in S1 Data. (TIF) [file pbio.3003563.s007.tif]

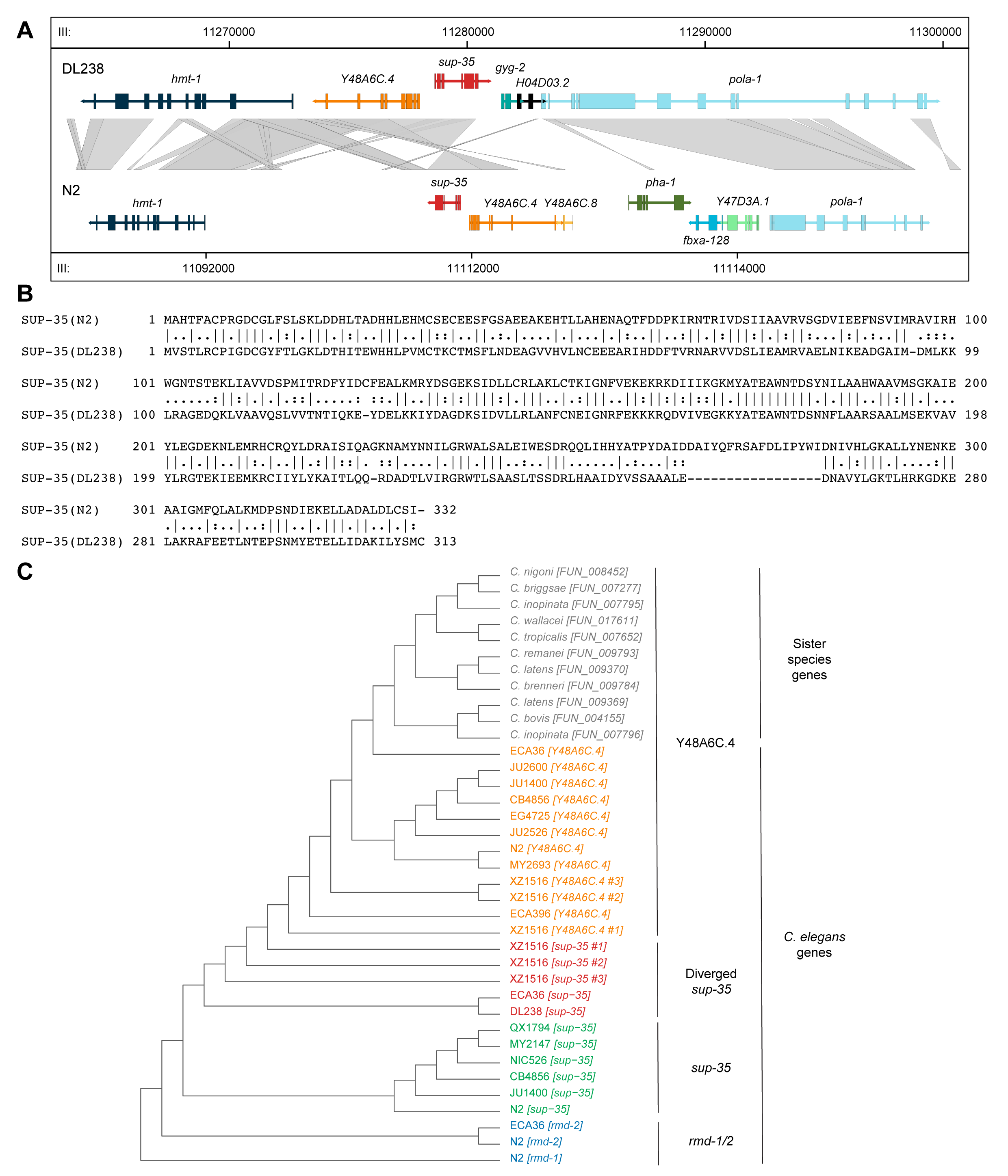

Supplement: S8 Fig — (A) A comparison of the sup-35/pha-1 locus between DL238 and N2 shows the deletion of the pha-1 gene in DL238. An inversion event caused divergence of sup-35. However, unlike previously reported, sup-35 is not pseudogenized in DL238. An ORF can be constructed from the sup-35 gene in DL238. (B) Pairwise alignment of SUP-35(N2) and SUP-35(DL238). (C) A phylogenetic tree of rmd-1/2, sup-35, and Y48A6C.4 homologs from various C. elegans wild isolates and other nematodes. The location of sup-35#1~3 and Y48A6C.4#1~3 in XZ1516 genome can be found in S1 Fig. (TIF) [file pbio.3003563.s008.tif]
