# Supplementary material for: sept-1/zina-1 is an ancient toxin-antidote system in Caenorhabditis elegans
Source: PLoS Biol. 2026 Jul 23;24(7):e3003563. doi: 10.1371/journal.pbio.3003563 (PMC13411925; doi:10.1371/journal.pbio.3003563)

Original gel image for Figure S4D

The *sept-1* blot

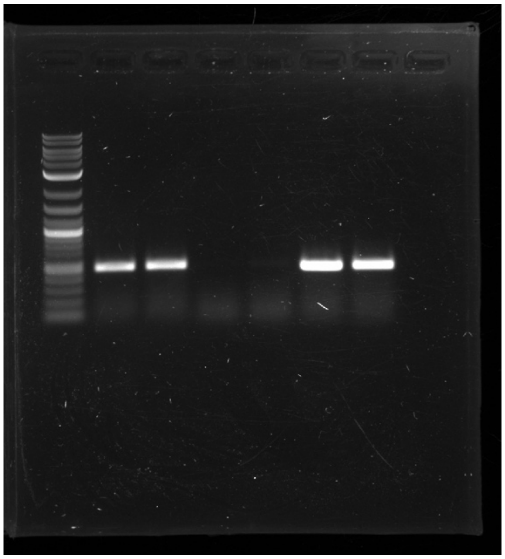

The *ama-1* blot.

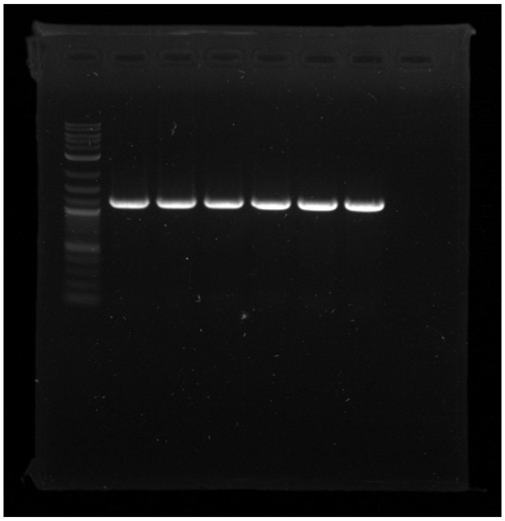

Original Western Blot image for Figure S5B

The rightmost two lanes are for 8 hours after heatshock, because the tubulin level is not consistent, we did not include the 8-hour data in the final results.

30 min heatshock; HA blot

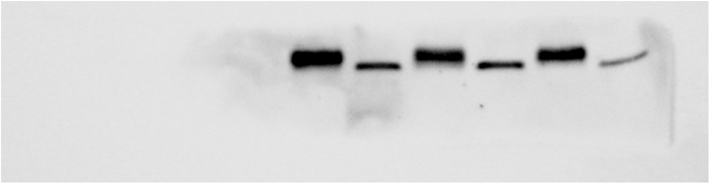

30 min heatshock; Tubulin blot

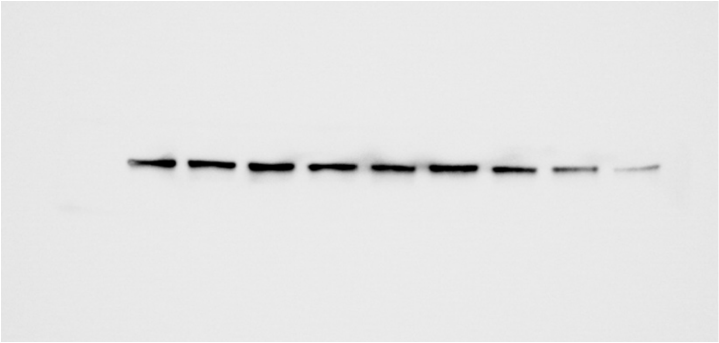

60 min heatshock; HA blot

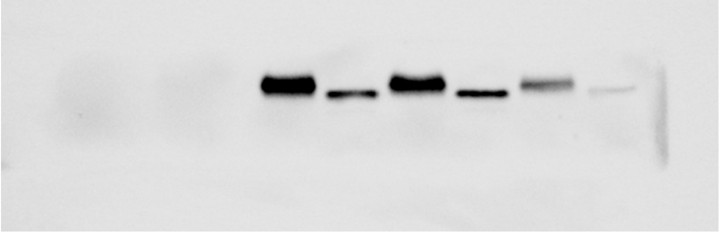

60 min heatshock; Tubulin blot

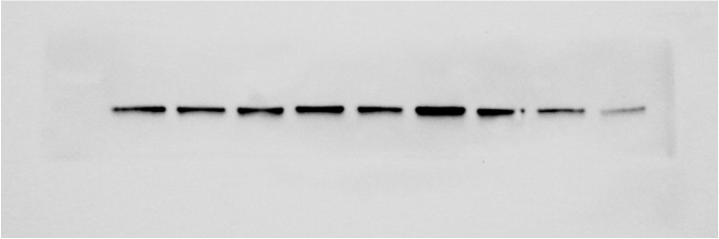

Supplement: S1 Raw Images — (PDF) [file pbio.3003563.s018.pdf]
