# Supplementary material for: sept-1/zina-1 is an ancient toxin-antidote system in Caenorhabditis elegans
Source: PLoS Biol. 2026 Jul 23;24(7):e3003563. doi: 10.1371/journal.pbio.3003563 (PMC13411925; doi:10.1371/journal.pbio.3003563)
Supplement: Attachment — Submitted filename: Point to point response.docx [file pbio.3003563.s021.docx]

Response to Reviewer Feedback for PBIOLOGY-D-25-03771R2

We thank all three reviewers for their constructive comments. A point-by-point response is provided below, with reviewers’ critiques in blue text and authors’ response in black text. We are also providing a “track-changes” style version of the manuscript that shows changes and edits.

Reviewers’ comments:

Reviewer #1:

In the manuscript by Liu and Zheng, the authors identified and studied a toxin-antidote (TA) system in C. elegans, which they called sept-1/zina-1. A recent paper (Zdraljevic et al., elife, 2025 preprint v1) has reported the same TA system with a different name mll-1/smll-1 in the wild C. elegans strain XZ1516 and explained the suppression of a divergent version of mll-1 in the reference C. elegans strain N2 by small RNA silencing. Although Liu and Zheng also identified the TA system in the strain XZ1516, they used different methods and concluded that the divergent version of sept-1 in the strain N2, which they named sept-2, is derived and no longer toxic. Overall, the authors provide solid evidence for most conclusions in the manuscript. The authors should however address the criticisms below.

Major criticisms:
1.	Naming of the genes:
a.	Because Zdraljevic et al. have identified the same system and reported them first, it would be less confusing for readers and the community if the same names were used in this work. In any case, C. elegans gene names should be registered with [genenames@wormbase.org](mailto:genenames@wormbase.org)

Response: We prefer to keeping the *sept-1* and *zina-1* names in the manuscript, as we identified them independently. We have registered *sept-1* and *zina-1* with [genenames@wormbase.org](mailto:genenames@wormbase.org), and Tim Schedl, who oversees the assigning of gene names, has listed *sept-1* as another name for *mll-1 (B0250.8)* and *zina-1* as another name for *smll-1(B0250.4)*. So, we think that it will be quite clear for the readers and community.

b.	The N2 version of sept-1 (if this gene were called 'sept-1') should be referred to in the format as sept-1(N2) or sept-1 in N2. It is not appropriate to call it sept-2, because sept-1(XZ1516) and sept-1(N2) are divergent alleles of the same gene with no ambiguity relative to their relationship as they are syntenic and single-copy.

Response: We thank the reviewer for the suggestion and have removed all the reference to “*sept-2*” and instead used “*sept-1(N2)*” to refer to the diverged toxin gene in N2.

2.	About the conclusion that SEPT-1(N2) has lost most of its toxicity:
a.	The direct evidence is Figure 3D, based on the comparison of a single-copy heat-shock construct. The reviewer could not find the number of assayed animals and whether the data derive from one or several lines. Please also verify that no artificial mutations were introduced into the transgene that may interfere.

Response: We added the experimental details to the Figure legends. We have sequenced and verified that there is no mutation in the transgene. We tested two independent lines and got similar results. For the data in Figure 3D, the data is from one line with 3-5 biological replicates (N ≥ 100 for each data point).

b.	The reviewer cannot see a single-copy experiment that compares the two alleles under their own or the same sept-1(XZ1516) cis-regulatory region, for example one such as in panel E compared in parallel to XZ1516, with clear indication of number of animals. (Recoding to remove potential piRNA sites in panel G may also affect the level of expression so the authors cannot use this experiment to conclude that SEPT-1(N2) has lost toxicity.)

Response: In Figure 3E, we have shown the genotyping data for the progeny of N2/XZ1516 in which the coding region of *sept-1(XZ1516)* is replaced by *sept-1(N2)*. In the revised manuscript, we added the control, which are the progeny of N2/wild-type XZ1516. Since XZ1516 carries the *sept-1(XZ1516)* driven by *sept-1(XZ1516)* promoter, we can compare the toxicity of *sept-1(N2)* and *sept-1(XZ1516)* both under the control of the endogenous *sept-1(XZ1516)* promoter. The results showed clear difference. *sept-1(XZ1516)* is so toxic that all progenies must carry at least one copy of the antidote, whereas *sept-1(N2)* is non-toxic and the progenies showed the Mendelian segregation (1:2:1) for the TA elements.

c.	Interpretation: To the reviewer, this study does not necessarily "contradict" (line 216) that of Zdraljevic et al but instead they may reflect quantitative, context-dependent effects. In Zdraljevic et al., the experimental approach appears to drive substantially higher expression levels than the method used here, which could account for the stronger or qualitatively different phenotypes observed. In the Figure 3D in this study, long exposure to heat-shock did cause larval arrest.

Response: We have removed the phrase “Our findings contradict the report by Zdraljevic *et al.*” in the revised manuscript.

d.	Wording about the "small RNA-mediated suppression" is unclear. Maybe adding "mechanism" at the end would help. For the reader to understand the context, it would be good that you expand a little more about what is known (presence of piRNAs, small RNA sequence data, etc.). Other small RNA pathways than those started by primary piRNAs may be active here. Are the small RNAs actually removed when the gene is recoded?

Response: In the revised manuscript, we have changed “small RNA-mediated suppression of expression” to “small RNA-mediated mechanisms”. We also added a sentence to further elaborate on the hypothesis by Zdraljevic et al., “Specifically, they propose that piRNA binding to the *sept-1(N2)* transcripts would lead to perpetual silencing by the 22G endo-siRNA pathway.” Since the recoding introduced 3~11 mismatches to the potential piRNA binding sites, we reason that they should block piRNA binding and thus prevent the generation of secondary siRNA. Following the same logic, this recoding of *sept-1(N2)* sequence likely blocks the binding of other small RNAs as well.

e.	Is sept-1(N2) expressed at a detectable level (using available expression data from the Andersen laboratory, in comparison with XZ1516)? Is its expression altered by recoding? line 221 refers to Fig. 3F but this is just the design of the recoding not the proof that sept-2 is expressed as suggested by the sentence.

Response: Using RT-PCR, we were not able to detect the expression of *sept-1(N2)*, but the recoding made it easily detectable (new Figure S4D). Thus, the recoding indeed enhanced the expression.

3. About sept-1 expression pattern: Transgenesis methods for the expression reporters are not described. Are they multicopy reporter or single-copy insertions like the rescue constructs? From the strain list, they seem to be multicopy. The expression site in somatic cells differs from that reported in Zdraljevic et al. (germline) using smFISH. Maybe this is due to the technique involved as transgenes are more likely silenced in the germline, especially if multicopy. This does not seem to be a good choice, and the caveat should at least be stated clearly. It is likely that the expression of single-copy transgenes is too weak to see it and that smFISH is indeed more appropriate.

Response: We indeed generated single-copy transcriptional reporters for *sept-1(XZ1516)* and *sept-1(N2)* but could not observe any fluorescent signals. So, we opted to use the multicopy arrays. In the revised manuscript, we mentioned this point and stated that the multicopy array is likely silenced in the germline.

4. The distribution of TAs can be summarized using a tree representation as in Figure 6. However, C. elegans populations do recombine so the tree representation may not reflect the history of the genomic region involved. The grouping in Hawaiian 1 and 2 seems artificial. It is thus not appropriate to draw evolutionary conclusions about which allele is ancestral, just based on this. In addition:
a.	Strain names cannot be seen in Figure 6. Please provide a supplemental file including the detailed information about the three TA existence status for the 611 C. elegans strains. Please also include notes about whether the version likely lost function, as in sept-1(N2) and sup-35(XZ1516).
b.	In Figure 6, how similar or divergent the sequence needs to be to the reference to be identified as "present" or "absent"? Maybe using gradient color? Please also include this information in the above supplemental file.

Response: In the revised manuscript, we modified Figure 6 to show groups of strains with unique genotypes for the three TA systems. The text in the Figure can be clearly seen now. A large phylogenetic tree containing all 611 strains and their TA information is shown in Figure S7. We also provided a supplemental Table S2 to include a detailed list of the strain information for the presence and absence of the toxin and antidote genes.

c.	Revise accordingly the two last paragraphs of the Discussion. Regarding the last paragraph, please note that some co-occurring TAs have been found in C. tropicalis (Noble et al. 2021, Ben-David et al. 2021).

Response: We rewrote the last two paragraphs of the Discussion and added citation to the two papers that also found co-occurrence of multiple TA systems.

5. The reviewer strongly suggests completely removing the last section of the Results and Figure 7. They cannot see evidence for a selective sweep marked by a lower pi diversity of the sept-1 allele. Fig. 7 rather show an increase in pi in this region and in any case the region is too large to conclude.

Response: Considering the reviewer’s comments, we have now removed Figure 7 and the last section of the Results. In the new Figure 7, we added more analysis on the evolution of the *sept-1/zina-1* locus among the wild isolates of *C. elegans*. Importantly, we found that in XZ1516, conserved genes adjacent to the *sept-1/zina-1* locus carry ancestral polymorphisms, supporting the idea that *sept-1/zina-1* is an ancient TA system in *C. elegans*.

6. The reviewer does not understand the Bayes Empirical Bayes analysis concluding to positive selection on the antidote gene in Fig. 2: in the context of these C. elegans polymorphisms, can you explain and provide methods? None is referenced.

Response: We have removed the results about positive selection in the revised manuscript.

Minor criticisms: 
1.	Provide the sequences of the constructs and CRISPR alleles.

Response: The sequences of DNA constructs are provided in Table S6. CRISPR allele sequences can found in Table S7.

2.	Table S4 "Cloning primers" is empty.

Response: We apologize for omitting the information of the cloning primers. The sequences of the primers used for cloning have been added to Table S5 now.

3.	In all figures or legends, please indicate the number of animals examined.

Response: We have now added the sample size information in the legends.

4.	Figure 1H, y axis label should read "Percentage of animals" (idem in other figures)

Response: Corrected.

5.	Figure 1I, the y-axis is confusing (no legend explaining the solid line versus the dots).

Response: The dots indicate XZ1516 genes in this region. The solid line represents the frequency of alternative alleles (N2 is the wild type) in the introgressed line (bin size is 200 kb). This information is now included in the Figure legend.

6.	Figure 2C and line 165: explain what you mean by identity (protein sequence?) and similarity (DNA level?). Typo on "similairy" on figure.

Response: Both identity and similarity are derived from protein sequence alignment. We corrected the typo on the Figure.

7.	Figure 4, the stem-loop part is perhaps interesting but a little distracting from the main topic of the paper.

Response: We agreed. But since it is part of our characterization of the TA system, we like to keep it in the paper. We shortened this part of the text in the revised manuscript.

8.	Line 90: remove the last sentence of the Introduction that is highly speculative.

Response: We have removed the sentence.

9.	line 221: rephrase. This is the rationale but you do not show that the expression is increased. (as mentioned above)

Response: We rephrased the sentence to be that “This recoding … would in theory allow *sept-1(N2)* to be expressed …”

10.	Line 314: remove "ecological".

Response: Agreed and removed.

11.	Line 383: add the allele/strain names for SEPT-1

Response: Corrected to be “… between SEPT-1(XZ1516) and SEPT-1(N2) …”

12.	Line 388: what is the evidence for stabilization in the germline?

Response: We have revised that sentence. Stem-loop structures in 3’UTR are known to promote mRNA stability and facilitate translation, but the A-to-I editing did not seem to play a major role.

13.	Line 496: provide the source of the genomes. Idem for RNAseq data when relevant.

Response: We added the genome versions.

14.	Typos/grammar in page 14, lines 461 (past tense), 464 (remove "do"); 
p. 15 lines 484 (to cross them), 485 (isogenic), 486 (crossed), 496 (each other's genome)
p. 16 lines 526 (involved), 538 typo
p. 18 lines 573 "and the"? "plates", 575 (Leica), 580 (Mixed), 581 (strain), 593 (extracted).
p.19 line 608 (resolutions).
Response: We sincerely thank the reviewer for pointing out these typos. All are corrected.

Reviewer #2: 

In this manuscript, Liu et al. present the genetic mapping and characterization of a novel toxin-antidote (TA) element in C. elegans. This new maternal-effect TA, sept-1/zina-1, was identified in the highly divergent isolate XZ1516 and causes larval lethality in F2 non-carrier individuals. The authors did an excellent job mapping both the toxin- and antidote-encoding genes, and the results are very clean. Although a related element was previously described by the Kruglyak lab (Zdraljevic et al., 2025), the strain collection and overall mapping strategy were different, so the two studies are complementary and together strengthen the conclusions.

Liu et al. also make several additional observations. In particular, they show that expression of the antidote in the embryonic gut is sufficient for rescue, suggesting that the toxin acts in this tissue. They report interesting A-to-I editing of the toxin mRNA, although this does not appear to affect toxic activity. Finally, and most importantly, the authors disagree with Zdraljevic et al. regarding the toxicity and regulation of the highly divergent toxin (sept-2) present in the N2 reference strain.

Overall, the manuscript is clearly written and well organized, but I have several technical comments and some concerns regarding data presentation.
Specific points

1. Toxicity of the N2 toxin (sept-2 / B0250.8 / mll-1)

The main disagreement between this study and Zdraljevic et al. (2025) concerns the intrinsic toxicity of the N2 toxin, B0250.8 (called sept-2 here and mll-1 in Zdraljevic et al.).

Zdraljevic et al. concluded that N2 mll-1 is toxic because overexpression using a tetracycline-inducible system caused abnormal phenotypes in N2, DL238, and XZ1516 backgrounds, similar to those observed upon overexpression of the XZ1516 toxin (sept-1). To explain why toxicity is not normally manifested in the N2 background (despite the absence of a functional antidote), they proposed that N2 mll-1 is repressed by a small-RNA pathway involving secondary 22G-RNAs. Although the evidence for this model is not particularly strong, it is plausible, and it is consistent with previous reports showing that B0250.8 is among the most strongly up-regulated genes in mut-16 mutants.

In contrast, Liu et al. conclude that sept-2 is not capable of poisoning N2. They generated single-copy transgenic lines expressing either sept-1 or sept-2 from a heat-shock promoter and found that a 20-30 min heat shock killed most larvae carrying the sept-1 transgene, whereas sept-2 transgenic larvae were largely unaffected. Interestingly, prolonged heat shock (50 min) of sept-2 transgenics did cause lethality, which the authors interpret as evidence for "weaker toxicity".
These experiments, however, lack a critical control: direct measurement of SEPT-1 and SEPT-2 expression levels. Although MosTI-based integration is reliable for transgene insertion, it does not guarantee expression. The piRNA (21U-RNA) pathway can recognize transgenes and induce somatic and/or germline silencing. Moreover, given prior evidence that sept-2 is regulated by small RNAs, the endogenous pre-existing 22G-RNAs may also target the transgene, resulting in reduced expression. The apparent "weaker toxicity" could therefore simply reflect lower protein levels, and dosage effects are well known in TA systems.

If strong conclusions are to be drawn about the relative toxicity of SEPT-1 and SEPT-2, transgene expression must be directly assessed, for example by western blotting or immunofluorescence. A straightforward approach would be to introduce an N- or C-terminal FLAG (or similar) tag. Importantly, the activity of the tagged protein should first be validated by tagging SEPT-1 at its endogenous locus and confirming that toxicity is retained.

Response: We thank the reviewer for the suggestion. As suggested by other reviewers, we have renamed *sept-1* as *sept-1(XZ1516)* and *sept-2* as *sept-1(N2)* and will refer to the two versions of *sept-1* accordingly in the below response. During the revision, we measured *sept-1(XZ1516)* and *sept-1(N2)* expression using both RT-qPCR and western blot (a HA-tag is fused to the C-terminus of the proteins). We found that the same heat-shock condition would induce similar mRNA expression, but the protein expression of SEPT-1(XZ1516) is stronger than that of SEPT-1(N2). The results are now added to Figure S5A-B. We reason that the SEPT-1(N2) proteins may have lower stability compared to SEPT-1(XZ1516) due to sequence divergence, which contributes to weaker toxicity.

The authors also attempted to test sept-2 toxicity by replacing the sept-1 coding sequence at the endogenous XZ1516 locus with sept-2, using a recoded version to remove putative piRNA target sites. They state that "this recoding likely removed the potential piRNA binding sites in the coding sequence and allowed sept-2 to be expressed, and its toxicity be revealed." However, the manuscript does not clearly describe how this recoding was performed. Which tool was used? Were known piRNA sequences explicitly avoided, or were synonymous mutations introduced more randomly under the assumption that this would disrupt piRNA targeting? This is not clear from the Methods or figure legends.

Response: We used this online tool to recode the *sept-1(N2)* sequence <https://worm.mpi-cbg.de/codons/cgi-bin/optimize.py> The “Resistance to RNAi of the original gene” option introduces synonymous mutations to almost every codon. We inspected the predicted piRNA binding sites in *sept-1(N2)* coding sequence and found that the recoding introduced 3~11 mutations to the potential piRNA target sites (Figure 3F), which would presumably block the binding of the piRNAs.

Furthermore, even if recoding successfully avoided N2 piRNAs (no evidence is provided), piRNA repertoires differ between strains. Because piRNAs are genomically encoded and polymorphic, one cannot assume that a sept-2 sequence recoded based on N2 information will escape silencing in XZ1516. Since the authors conclude that sept-2 is not toxic, it is essential to demonstrate that the recoded gene is in fact expressed in the relevant tissues and stages.

Response: We thank the reviewer for the comment. We have conducted RT-PCR experiment to detect the expression of *sept-1(N2)* and *sept-1(N2; recode)*. We could not detect the mRNA expression of *sept-1(N2)* but were able to detect *sept-1(N2; recode)*, suggesting that the recoding indeed enhanced the expression of *sept-1(N2)*. smFISH against *sept-1* does not work in our hands, so we could not detect where the recoded *sept-1(N2)* is expressed. We reason that the recoding should not affect somatic expression of *sept-1(N2)*, since piRNAs do not work in somatic tissues. Thus, the enhanced expression likely results from germline expression of the recoded *sept-1(N2)*.

2. Reporting of sample sizes and raw counts

Throughout the manuscript, I could not find clear information on N (number of independent experiments) and n (sample size per experiment) for most crosses and assays. Only percentages and p-values are typically reported. For reproducibility and transparency, I strongly encourage the authors to provide raw counts and full experimental details in the supplementary material and to reference sample sizes consistently in the main text and figure legends.

Response: We have added information about the sample size and the number of replicates in Figure legends.

3. Direction of genetic crosses

Related to the previous point, the direction of many crosses is not specified. For example, in the initial result showing that ~40% of progeny from N2 × XZ1516 crosses display developmental defects, it is unclear whether XZ1516 hermaphrodites were crossed to N2 males or vice versa. Previous work (Pliota et al., 2024) has demonstrated that cross direction can critically affect TA activity in C. tropicalis; for instance, the maternal-effect slow-1/grow-1 system is only active when paternally inherited. For completeness and clarity, the direction of all crosses should be explicitly stated.

Response: In all the results shown in the paper, we used N2 males to cross with XZ1516 hermaphrodites to obtain the N2/XZ1516 hybrids, of which the progenies showed biased segregation of the *sept-1/zina-1* TA system. We have also tested crossing in the reverse direction (using XZ1516 males and N2 hermaphrodites to generate N2/XZ1516 hybrids) and obtained similar results, suggesting a lack of parent-of-origin effects in this case. We added the information in the revised manuscript.

4. Identity and nomenclature of sept-1/zina-1 and mll-1/smll-1

At line 183, the authors state that the TA system described by Zdraljevic et al. is "likely the same as" sept-1/zina-1. There is, in fact, no ambiguity here: sept-1/zina-1 and mll-1/smll-1 correspond to the same locus and should be described as such.
In addition, I am not sure whether it is advisable to introduce new gene names (sept-1 and zina-1) in this context. In general, redundancy in gene nomenclature should be avoided whenever possible. Here, it is clear that the genes in question are identical to those previously described, and there seems to be no strong justification for maintaining two parallel naming schemes for the same TA system.

Response: We have removed the word “likely” and asserted that *sept-1/zina-1* is the same as *mll-1/smll-1* in the revised manuscript. Since we identified this TA system independently, we like to keep our naming system. We have contacted [genenames@wormbase.org](mailto:genenames@wormbase.org) and registered *sept-1* and *zina-1* already. *sept-1* is currently listed as another name for *mll-1(B0250.8)* and *zina-1* as another name for *smll-1(B0250.4)*. So, we think that it will be quite clear to the readers and community.

5. Mutual exclusivity of maternal TA systems

At line 417, the authors note that sept-1/zina-1 and sup-35/pha-1 are almost mutually exclusive, and suggest that this may reflect an energetic cost of maintaining two maternal TA systems. While the observation is interesting, previous work (Ben-David et al., Current Biology, 2021) showed that some C. tropicalis isolates (e.g., NIC203 and EG6180) carry two or even three active maternal-effect TAs, arguing against a strong general fitness cost of harboring multiple such systems. The apparent anti-correlation between sup-35/pha-1 and sept-1/zina-1 could therefore be coincidental. I would recommend either toning down this interpretation or, if the authors wish to emphasize it, directly testing the hypothesis by comparing the fitness of NILs carrying both TA systems with appropriate single-TA and double-knockout controls. Overall, claims about the relative age of the TAs should be toned down. The evidence is rather indirect.

Response: We have removed the above point in the revised manuscript. Moreover, we cited the previous papers (Noble et al. 2021, Ben-David et al. 2021) that show *C. tropicalis* isolates can carry multiple maternal-effect TAs, which supports the findings that some *C. elegans* strains carry more than one TAs.

6. SEP-domain

The authors named the toxin after its SEP-domain. I may have missed this, but there was no reference or explanation to what is known about this protein domain.

Response: SEP (after shp1, eyc and p47) domain is a eukaryotic domain that is frequently followed by a UBX domain. Indeed, the sequence following the SEP domain in SEPT-1 is aligned to *C. elegans* UBXN-2. The function of SEP domain is unclear. One study suggests that it may inhibit lysosomal cysteine protease cathepsin L (PMID: 15498563). We have added some more description in the revised manuscript.

Reviewer #3:
[identify themselves as Daniel Park and Michael Ailion]

Toxin-antidotes (TAs) promote their own inheritance with little or no benefit to their host. TAs are widespread in prokaryotes, and they are now being found and characterized in eukaryotes. In this manuscript, the authors identify and characterize a new TA in a divergent Hawaiian strain of C. elegans, the third one found in this species. Strong genetic evidence is provided that the sept-1 toxin is maternally transmitted and causes larval arrest in the absence of its zygotically expressed antidote zina-1. Additional convincing experiments show that in the standard lab strain N2, the toxin gene has been retained but has lost toxicity due to sequence divergence, while the antidote has become pseudogenized. The manuscript concludes with an interesting phylogenetic analysis of the three known TAs in C. elegans, suggesting that the two known maternal effect TAs do not coexist in the same strain. Overall, the work is performed well and most of the conclusions are supported by the data. This is an interesting and solid new addition to the growing literature in the field of eukaryotic TAs.

Considerations

1. The larval arrest phenotype is described as "rod-like." This designation has previously been used to describe the larval arrest phenotype of mutants in the Ras pathway, due to malfunction of the excretory duct system and defects in osmoregulation. But it is not clear whether the larval arrest phenotype due to sept-1 toxicity is this same rod-like phenotype. First, the arrested larvae shown in this manuscript are not straight rods, but have a curved posture (e.g. Fig 5C&D and Fig S2A). Second, given that the toxins causes defects in the morphology of the intestine and the zina-1 antidote acts in the intestine, it is unclear whether this is equivalent to the previously described rod-like arrest of mutants that affect the excretory system. This issue warrants further discussion, and without further evidence, it would be prudent to use a term other than "rod-like" for the arrest phenotype.

Response: We sincerely thank the reviewer for pointing out this issue. First, the rod-like phenotype is quite clear on NGM plates. However, when we mount the animals onto the agarose pad, the arrested larvae sometimes look less straight, which is a technical issue. Animals in Figure 5D look particularly curved, because the *elt-2p::GFP::H2B* transgene that labels the intestine used *rol-6(D)* as the co-injection marker (we added a note to the Figure legend). Second, Zdraljevic *et al.* also referred to the larval arrest phenotype as the “rod” phenotype and mentioned that it is reminiscent of the rod-like lethal phenotype observed in the Ras pathway mutants (Rocheleau et al., 2002). To keep it consistent, we prefer to also call the arrest phenotype “rod-like” larval arrest. Third, in the Discussion of the revised manuscript, we also mentioned the phenotype previously observed in Ras mutants and suggested that the same rod-like phenotypes may have different underlying reasons.

2. It is unclear if sept-1 is only capable of toxicity at the L1 larval stage. What happens if animals carrying hs::sept-1 are heat-shocked as older animals? Is there any evidence for toxicity in tissues other than the intestine?

Response: In the revised manuscript, we conducted additional experiments to heat-shock the animals at the L4 stage and found that induced expression of SEPT-1(XZ1516) could also generate lethality in L4 animals, although the lethality is lower compared to heat-shock at L1 stage. Again, the toxicity of SEPT-1(N2) is weaker than SEPT-1(XZ1516). In addition, we observed the shrinkage of the intestine, as the dorsoventral width of the intestine became smaller than the wild-type, and gaps emerged between the intestine and the body wall muscles. We quantified this morphological change and found that the phenotype is more pronounced with the expression of SEPT-1(XZ1516) compared to the expression of SEPT-1(N2). The new data are now shown in Figure S5C-D.

3. Lines 279-284 and Figure S5. Based on images of sept-1p and sept-2p transcriptional reporters, it is concluded that sept-1 is expressed at a higher level. This is not convincing and the conclusion should be toned down. First, there are only single images with no quantification. Second, these strains carry extrachromosomal arrays, which are overexpressed and may be at different copy levels. There is often a lot of variability between independent arrays of the same construct, so it is not meaningful to quantitatively compare individual arrays of two different constructs. It is unclear how many arrays of each construct were generated, but quantitative analysis of a large number of arrays of each construct would be necessary to even begin to be meaningful. Single-copy insertions at the same chromosomal locus would provide stronger evidence.

Response: We have indeed constructed single-copy *sept-1(XZ1516)p::mScarlet3* and *sept-1(N2)p::mScarlet3* transcriptional reporters, but neither of them showed any fluorescent signal. We reason that the expression level of the toxin is generally low and may be below the detection limit of single-copy methods. So, we created multicopy arrays to assess their promoter activities. In the revised manuscript, we added quantification of the fluorescent signals generated by the arrays to Figure S6. We indeed created multiple lines for each reporter, and the expression pattern and intensity are similar across the lines.

4. The conclusions in the final section of the manuscript about how TA systems influence genetic diversity are not convincing. In Fig 7, sept-1(-) has a slightly higher rate of polymorphisms at the sept-1/zina-1 locus compared to sept-1(+), yet there is an even larger increase in polymorphisms further to the right, away from the locus at Mb 20.59-20.62. But at Mb 20.50-20.53 (between sept-1/zina-1 and the peak at Mb 20.60), sept-1(+) has higher polymorphisms than sept-1(-). It is unclear whether any of these effects are statistically significant. In fact, the absence of sup-35 appears to have a greater effect on polymorphisms at the sept-1/zina-1 locus than the absence of sept-1. The analysis of Tajima's D statistic is also unconvincing. As with the analysis of polymorphism rate, there is no statistical analysis to determine whether the small effects seen are statistically significant. In fact, if anything, sup-35(-) appears to have a more negative Tajima's D than sup-35(+) near the sup-35/pha-1 locus which is the opposite of what is stated in lines 357-359, "Supporting this idea, we found more negative Tajima's D in the region linked to sept-1(+) or sup-35(+) compared to the same region in strains without the TA system." Overall, the data presented here do not support the conclusion that the presence of a TA leads to reduced diversity.

Response: We have removed the original Figure 7. In the new Figure 7, we showed additional evidence to support that *sept-1/zina-1* is the ancestral TA system in *C. elegans*.

5. In general, the Methods sections are brief and not very specific. The following Methods sections should be improved:

a. Concentrations of DNAs injected to make transgenic lines should be provided. This information could be added to Table S2. Co-injection markers should be specified.

Response: We have added the information about the concentration of the DNA constructs injected to make the transgenic lines to Table S3 (previous Table S2). The information of co-injection markers was included in the genotype of the strains in the same Table.

b. For CRISPR, more details should be provided, including concentrations of reagents and what co-CRISPR marker was used. Primers used to generate repair templates do not seem to be provided in Table S4. Also, it is stated that PCR genotyping was used to screen for CRISPR edits, but it is unclear what primers were used. These primers do not seem to be provided in Table S4. There are some genotyping primers shown in Table S4, but these are presumed to be just the primers used to genotype the sept-1, sept-2, and zina-1 alleles, not for screening CRISPR edits. What these are should be clarified.

Response: For CRISPR, we injected 2 μM recombinant SpCas9 protein with *in vitro* transcribed single-guide RNA (at 100 ng/μl). For the co-CRISPR, we used *dpy-10(cn64)* as the co-conversion marker. The information is now provided in the Materials and Methods. We also added more information about the genotyping primers.

c. Heat-shock temperature is not provided.

Response: We performed heat induction at 33 degrees Celsius. This information is now added.

d. Methods of cloning and generating DNA constructs are not described fully. What cloning methods were used? Also, cloning primers should be listed in Table S4.

Response: We performed Gibson assembly for all cloning in this study using the reagent from Vazyme (Nanjing, China). We sincerely apologize for omitting the information of the cloning primers, which are now added in Table S5.

e. Antibody staining: concentrations of antibodies should be provided as well as the buffer they are diluted into.

Response: The antibodies were diluted at 1:1000 fold. This information is added.

f. smFISH: concentrations and sequences of probes should be provided.

Response: The final concentration of the smFISH probes is 125 nM in the experiment. The probe sequences are now provided in Table S8.

g. RNAseq: description of how worms were grown and RNA isolated should be provided.
Response: We collected mixed-stage worms from three 100mm NGM plates and then extracted total RNAs using Trizol. This information is now provided in the Materials and Methods.

Minor points

1. The sept-1/zina-1 TA has also been reported by another lab (Zdraljevic et al., PMID: 39605437) who called it mll-1/smll-1. At two points, (lines 185 & 365), sept-1/zina-1 is described as "likely the same" as mll-1/smll-1. Use of the term "likely" is confusing, as the two papers identify the same genes.

Response: We have now removed the term “likely” at the two places.

2. A rather lengthy section of the results is devoted to RNA editing in the 3'UTR of the sept-1 mRNA. This section is tangential to the main storyline and ultimately concludes with a negative result: RNA editing has little effect on the sept-1 mRNA stability and is not required for toxicity. We feel that this section could be dramatically shortened, with the data in a supplementary figure where they won't distract from the main narrative.

Response: We have shortened the paragraph but decided to keep it in the main Figures.

3. Discussion, Lines 387-391. This paragraph makes unsupported claims about the promoter of sept-1 being stronger than sept-2 (see point #3 above), and the importance of the sept-1 3'UTR stem-loop and RNA-editing to stabilizing sept-1 mRNA. The data however show that RNA editing does not significantly stabilize the sept-1 mRNA and whether the stem-loop is stabilizing is not addressed. We would suggest cutting this paragraph.

Response: We added quantifications to support that *sept-1(XZ1516)* promoter is stronger than *sept-1(N2)* promoter to Figure S6 (see response to above point #3). For the other points, we have modified the statements to avoid making unsupported claims. In the revised manuscript, we simply mentioned the possibility that the stem-loop structure in *sept-1(XZ1516)* 3’UTR might help promote mRNA stability and translation efficiency as 3-terminal stem-loop is known to do so (PMID: 8657580).

4. Discussion, Lines 392-401. This is a highly speculative paragraph that makes assertions based on data not described in the Results sections. Line 393: it is asserted that ZINA-1 is not localized in the nucleus, but this has not been convincingly demonstrated. The images in Fig 5A&B are not shown at the resolution needed to make such a claim. Line 397: it is asserted that GFP::ZINA-1 forms aggregates. It is unclear what is considered an aggregate in Fig 5A&B - arrows in these figures would help. There are perhaps some punctate, but proteins can appear punctate for reasons other than aggregation (e.g. membrane association). Additionally, zinc-finger proteins are not exclusively DNA or RNA-binding proteins as implied here. They can also bind other proteins and function in a wide range of processes (see PMID 29152378 for example). Thus, ZINA-1 could have a number of potential antidote functions outside the nucleus that don't involve RNAs. Based on the data, hypothesizing that ZINA-1 is involved in liquid-liquid phase separation is highly speculative.

Response: We have now deleted any mentioning about ZINA-1 aggregates and liquid-liquid phase separation. We have also mentioned the possibility that ZINA-1 may function in an RNA-independent manner. Nevertheless, we think our imaging data indicate that ZINA-1 is not nuclear localized. If one compares Figure 5D WT (GFP::H2B in the intestinal nuclei) with Figure 5B L1, one can clearly see that ZINA-1::GFP is not enriched in the nucleus as a nuclear protein does.

5. sept-1p shows somatic expression in the intestine and pharynx in both L1 larvae and adults (Fig S5). This is not discussed. Is it possible that, in addition to maternally-delivered sept-1, somatic expression also contributes to toxicity? Also, is the endogenously-tagged sept-1::tagRFP expressed in larvae and adults? If so, where does the protein localize?

Response: We could not detect any signal for endogenously tagged SEPT-1::TagRFP likely due to its low expression level. Single-copy *sept-1p::mScarlet3* did not generate any fluorescent signal either. We were only able to detect expression of the transcriptional reporters using multicopy arrays. In general, we do not think that the somatic expression of *sept-1* is essential for toxicity, since the animals that do not carry a zygotic copy of *sept-1* would be arrested at early larval stages as long as they inherited maternal *sept-1*. Nevertheless, it is unclear whether the somatic expression of the *sept-1* enhances the toxicity or has other functions. We added some more discussion to this point.

6. For the crosses in Fig 1, it would be useful to show the parental generation cross used to generate the N2/XZ1516 hybrid -- i.e. which parent was male and which was hermaphrodite? Does the direction of the parental cross matter? Given that some maternally inherited incompatibility genes show a maternal cytoplasmic pattern of inheritance, that possibility should be accounted for here.

Response: This is a good point and is also mentioned by reviewer 2. In general, we created the N2/XZ1516 hybrids by crossing N2 males with XZ1516 hermaphrodites and observed the arrested larval among the progeny of the hybrids. In light of the suggestion by both reviewers, we also generated the N2/XZ1516 hybrids by crossing XZ1516 males with N2 hermaphrodites and observed the same rod-like phenotype in the progeny of those hybrids, suggesting a lack of parent-of-origin effects in this case.

7. Line 163. What is a SEP domain and generally what do they do? Providing a domain map of SEPT-1 would be useful.

Response: SEP (after shp1, eyc and p47) domain is a eukaryotic domain that is frequently followed by a UBX domain. The function of SEP domain is unclear. One study suggests that it may inhibit lysosomal cysteine protease cathepsin L (PMID: 15498563). There is already a domain map of SEPT-1 in Figure 2C.

8. Outside of C. elegans, are there any homologs of sept-1 and zina-1?

Response: Outside of C. elegans, we did not find homologs of *sept-1* and *zina-1*. They appear to be *C. elegans*-specific genes. We added this information in the Results.

9. Lines 404-406: "Hawaiian_2 strains represent a transition period when the sept-1/zina-1 was gradually lost through the accumulation of missense mutations in the sept-1 coding sequences." This implies that these strains have an intermediate number of missense variants. Is this true?

Response: We have deleted that sentence and removed the grouping of Hawaiian_1, _2, and _3. In the revised Figure 6, we simply grouped the strains into Hawaiian and non-Hawaiian strains. We examined the coding sequence of *sept-1* among the strains and did not find a clear intermediate state. There are simply two haplotypes; one looks like *sept-1(XZ1516)* and the other like *sept-1(N2)*. Nevertheless, we found a few strains where *sept-1* is deleted. This information is now added to Figure 7.

10. Lines 414-416: as an alternative to the idea that peel-1/zeel-1 emerged early in the Hawaiian lineage but didn't propagate, is it possible that peel-1/zeel-1 did not exist in the Hawaiian ancestor, but instead was acquired later in certain Hawaiian strains by mating and recombination with peel-1/zeel-1 strains (i.e. this chromosomal region was transferred while the rest of the genome remained Hawaiian?). It seems that it is tricky to make strong conclusions about the age of a chromosomal segment from the overall phylogenetic tree, because the phylogeny of specific chromosomes or genes may not match the overall tree.

Response: We have removed the statement in the revised manuscript. We think both scenarios are possible, but we currently do not have sufficient evidence to support either case. The gene next to *peel-1* is *nekl-1* (Figure S1). We analyzed the SNPs in *nekl-1* and found that XZ1516 carries ancestral alleles of *nekl-1*, while N2 carries a more derived allele, which appear to argue against the idea that this *peel-1/zeel-1* locus in XZ1516 was obtained by crossing with non-Hawaiian strains. Moreover, we found that *peel-1* in XZ1516 carries the variant F97Y, which was also found in four strains that have pseudogenized *zeel-1*. So, one possibility is that *peel-1* in XZ1516 may not be toxic and it evolved into the *peel-1(N2)* version that is toxic. Nevertheless, we do not want to overinterpret these observations.

11. Is sept-2 the proper nomenclature for the sept-1 ortholog in N2? They are both the same gene in the same species, only with some sequence divergence. It seems like calling this gene sept-1(N2) might be more appropriate.

Response: We have changed the *sept-2* to be *sept-1(N2)*. The original *sept-1* is referred to as *sept-1(XZ1516)*.

12. Line 290 and Figure 5A: Though GFP::ZINA-1 co-localizes with ges-1p::RFP, it does not show "clear enrichment." This should be toned down.

Response: We have toned down the statement.

13. Line 300 & Fig 5C legend: why are these worms described as "dried up"? In the classic rod-like larval arrest, worms are typically described as "fluid-filled."

Response: We realize it may be an incorrect description of the phenotype and have removed the phrase. What we really observed is the shrinkage of the intestine. Both in the arrested larva produced by the N2/XZ1516 hybrids and generated in the heat-shock experiment, we saw the dorsoventral width of the intestine became smaller than the wild-type, and the intestine appeared to be separated from the body wall muscle.

14. Figures or legends should include N of worms scored.

Response: This information is added to the Figure legends.

15. All micrograph scale bars should be defined in the figure legends. For example: Fig 3D, Fig 4A, Fig 5B-E.

Response: Scale bar information is now added.

16. In figure panels with multiple Chi-square tests, there should be statistical correction for multiple comparisons.

Response: We did Bonferroni correction to correct the *p* value for multiple Chi-square test. This information is now added to the Materials and Methods.

17. Figure 6: there appear to be a couple instances where peel-1 is present while zeel-1 is absent. Could the authors comment on this, since peel-1(+)/zeel-1(-) animals should not be viable.

Response: Upon close inspection, we indeed found six strains in which *peel-1* is present and *zeel-1* is absent. Among the six strains, the four Hawaiian ones (ECA701, ECA1769, ECA2091, and ECA2191) all carry a F97Y variant in *peel-1*. So, one hypothesis is that this substitution might render PEEL-1 non-toxic. For the non-Hawaiian JU1652 and NIC166, we found no nonsynonymous mutations in *peel-1* despite the loss of *zeel-1*. Either the *zeel-1* annotation is incorrect due to low genome quality, or an alternative detoxification mechanism exists. We have added the above details in the revised manuscript.

18. Figure 6: it would be useful to provide strain names in a supplementary Table for the 611 worm strains and their TA genotypes.

Response: We have created a new Table S2 to provide this information.

19. There should be a key for Table S1, explaining what each of the column headings means. Many of these are nonintuitive.

Response: We have now added supplemental Table legends, which included detailed information for the meaning of column headings for Table S1.

20. In Table S4, CRISPR repair templates should be briefly described, not just "recombinant DNA."

Response: This information has been added to the supplemental Table S7 in the revised manuscript.

21. Table S5 should list the vector backbones of the plasmid constructs.

Response: The full sequence of the plasmid constructs can be found in the supplemental Table S6 now.

22. In many of the micrographs, it would help non-worm readers if the images included arrows pointing to specific features of interest. For example, pointing out the deformed pharynx in Fig 1A.

Response: We thank the reviewer for the suggestion and have added some arrows.

23. Figure 1A: for clarity, the figure should say "Type I" or "Type II," not "Arrested larva-I" or "Arrested larva-II"

Response: Corrected in the Figure.

24. Figure 1B-F: a Punnett square showing the result of the cross and how/why each case of deviance from 75% marker is informative would be useful, especially for readers from non-genetics backgrounds.

Response: A typical segregation pattern is shown in Figure 1B-C. So, we think a Punnett square may not be necessary. In the Figure legends, we added that “75% animals were expected to carry the dominant marker if it is not linked to the TA system.”

25. In Fig S6, shading of identical and similar residues would help to visualize the alignment.

26. The use of a white font on a gray background in Fig 7A is hard to read.
Response: The original Figure 7 has now been removed.

27. In Fig 7B (left panel), it would help to use colors that are more different than blue and green. 
Response: The original Figure 7 has now been removed.

28. In Fig S1D & E: why are there male symbols in the final generation when worms were scored? Were only male worms scored?

Response: Yes. Among the live progeny, we only scored the males, which are for sure the cross progeny. We added this note to the Figure legend.

29. Line 125: make it clear that 15-22 Mb refers to chromosomal position, not the size of the mapped region because this was confusing when first read.

Response: We changed it to “Chr V: 15-22 Mb” to avoid the confusion.

30. Line 141: change "The rest exons…" to "The rest of the exons…"
Response: Corrected.

31. Line 221: "allowed sept-2 to be expressed and its toxicity be revealed." The writing here is confusing and makes it sound like sept-2 is toxic, when in fact, the opposite is concluded. Better would be "allowed sept-2 to be expressed and its possible toxicity to be tested."
Response: We thank the reviewer for the suggestion, and the phrase is corrected.

32. Line 247-248: sept-1 mRNA being deposited into oocyte is also reported by Zdraljevic et al., and this should be noted as a similarity between the studies.

Response: We have added a sentence to describe the similarity.

33. Line 299: DIC should be spelled out.

Response: We spelled it out as “differential interference contrast microscopy”.

34. Lines 354 and 359: include which panel of Figure 7.

Response: Figure 7 is now removed.

35. Line 381: is it correct to call this the "endogenous RNAi pathway"?
Response: We have changed it to “endogenous small RNAs”.

36. Line 782: include reference for Bayes Empirical Bayes analysis in the figure text. This method should also be described in the Methods section.
Response: We have removed the analysis on the positively selected sites and hence did not need to mention Bayes Empirical Bayes analysis in the revised manuscript.

37. There are several incorrect figure call-outs:
line 179: not Fig S2C
line 303: should include Fig 5E
lines 321 & 330: there are no Fig 6A & B, just Fig 6.
Response: Corrected.

38. There are a number of typos: 
line 38: should be "addiction"
line 383: should be "between SEPT-1 and SEPT-2"
line 451: should be "Center"
line 520: should be "IPTG"
line 538: should be "substitution"
line 575: should be "Leica"
line 581: should be "strain"
line 594: the period should be changed to a comma: "…wild isolates, we combined…".

Response: We thank the reviewer for pointing out the typos. They are all corrected in the revised manuscript.
