# Supplementary material for: sept-1/zina-1 is an ancient toxin-antidote system in Caenorhabditis elegans
Source: PLoS Biol. 2026 Jul 23;24(7):e3003563. doi: 10.1371/journal.pbio.3003563 (PMC13411925; doi:10.1371/journal.pbio.3003563)
Supplement: Peer review history

# Editor report: Decision Letter 0

**DOI:** 10.1371/journal.pbio.3003563.r001

**Authors:** Roland G Roberts

26 Nov 2025

Dear Dr Zheng,

Thank you for submitting your manuscript entitled "sept-1/zina-1 is an Ancient Toxin-Antidote System in Caenorhabditis elegans" for consideration as a Research Article by PLOS Biology.

Your manuscript has now been evaluated by the PLOS Biology editorial staff, as well as by an academic editor with relevant expertise, and I'm writing to let you know that we would like to send your submission out for external peer review.

IMPORTANT: According to our scooping policy, we will explicitly ask the reviewers to ignore the recent related eLife publication from the Kruglyak lab when assessing novelty.

However, before we can send your manuscript to reviewers, we need you to complete your submission by providing the metadata that is required for full assessment. To this end, please login to Editorial Manager where you will find the paper in the 'Submissions Needing Revisions' folder on your homepage. Please click 'Revise Submission' from the Action Links and complete all additional questions in the submission questionnaire.

Once your full submission is complete, your paper will undergo a series of checks in preparation for peer review. After your manuscript has passed the checks it will be sent out for review. To provide the metadata for your submission, please Login to Editorial Manager (https://www.editorialmanager.com/pbiology) within two working days, i.e. by Nov 28 2025 11:59PM.

If your manuscript has been previously peer-reviewed at another journal, PLOS Biology is willing to work with those reviews in order to avoid re-starting the process. Submission of the previous reviews is entirely optional and our ability to use them effectively will depend on the willingness of the previous journal to confirm the content of the reports and share the reviewer identities. Please note that we reserve the right to invite additional reviewers if we consider that additional/independent reviewers are needed, although we aim to avoid this as far as possible. In our experience, working with previous reviews does save time.

If you would like us to consider previous reviewer reports, please edit your cover letter to let us know and include the name of the journal where the work was previously considered and the manuscript ID it was given. In addition, please upload a response to the reviews as a 'Prior Peer Review' file type, which should include the reports in full and a point-by-point reply detailing how you have or plan to address the reviewers' concerns.

During the process of completing your manuscript submission, you will be invited to opt-in to posting your pre-review manuscript as a bioRxiv preprint. Visit http://journals.plos.org/plosbiology/s/preprints for full details. If you consent to posting your current manuscript as a preprint, please upload a single Preprint PDF.

Feel free to email us at plosbiology@plos.org if you have any queries relating to your submission.

Kind regards,

Roli Roberts

Roland Roberts, PhD

Senior Editor

PLOS Biology

rroberts@plos.org

# Peer review: Decision Letter 1

**DOI:** 10.1371/journal.pbio.3003563.r002

**Authors:** Roland G Roberts

4 Feb 2026

Dear Dr Zheng,

Thank you for your patience while your manuscript "sept-1/zina-1 is an Ancient Toxin-Antidote System in Caenorhabditis elegans" was peer-reviewed at PLOS Biology. It has now been evaluated by the PLOS Biology editors, an Academic Editor with relevant expertise, and by three independent reviewers. Please accept my apologies for the delay incurred over the holiday period.

You'll see that reviewer #1 is positive but makes some suggestions about gene nomenclature, wants some clarification around the claim that SEPT-1(N2) has lost toxicity, more detail around the transgenesis methods, questions some aspects of Fig 6, and suggests removal of Fig 7. Reviewer #2 is also positive, and sees this as complementary to the Zdraljevic et al study; however, s/he says that you need to measure expression levels of the SEPT-1 and SEPT-2 transgenes in order to support their stronger claims, complains about poor reporting of sample sizes and genetic cross directions, and raises a similar point to rev #1 about gene nomenclature. Reviewer #3 is again positive, but raises related issues about transgene expression, finds Fig 7 unconvincing (like rev #1), wants significantly more detail in the Methods, and has a list of presentational and textual requests.

IMPORTANT: I discussed these comments with the Academic Editor, who said, "I think that the methodological and reporting points made are quite significant and its important that the authors recognise the importance of addressing these thoroughly. Additionally, the 'selective sweep' section of the paper is very weak and should be removed as suggested by the reviewers."

In light of the reviews, which you will find at the end of this email, we would like to invite you to revise the work to thoroughly address the reviewers' reports.

Given the extent of revision needed, we cannot make a decision about publication until we have seen the revised manuscript and your response to the reviewers' comments. Your revised manuscript is likely to be sent for further evaluation by all or a subset of the reviewers.

In addition to these revisions, you will need to complete some formatting changes, which you will receive in a follow up email. A member of our team will be in touch with a set of requests shortly.

We expect to receive your revised manuscript within 3 months. Please email us (plosbiology@plos.org) if you have any questions or concerns, or would like to request an extension.

At this stage, your manuscript remains formally under active consideration at our journal; please notify us by email if you do not intend to submit a revision so that we may withdraw it.

**IMPORTANT - SUBMITTING YOUR REVISION**

Your revisions should address the specific points made by each reviewer. Please submit the following files along with your revised manuscript:

1. A 'Response to Reviewers' file - this should detail your responses to the editorial requests, present a point-by-point response to all of the reviewers' comments, and indicate the changes made to the manuscript.

*NOTE: In your point-by-point response to the reviewers, please provide the full context of each review. Do not selectively quote paragraphs or sentences to reply to. The entire set of reviewer comments should be present in full and each specific point should be responded to individually, point by point.

You should also cite any additional relevant literature that has been published since the original submission and mention any additional citations in your response.

2. In addition to a clean copy of the manuscript, please also upload a 'track-changes' version of your manuscript that specifies the edits made. This should be uploaded as a "Revised Article with Changes Highlighted" file type.

*Re-submission Checklist*

When you are ready to resubmit your revised manuscript, please refer to this re-submission checklist: https://plos.io/Biology_Checklist

To submit a revised version of your manuscript, please go to https://www.editorialmanager.com/pbiology/ and log in as an Author. Click the link labelled 'Submissions Needing Revision' where you will find your submission record.

Please make sure to read the following important policies and guidelines while preparing your revision:

*Published Peer Review*

Please note while forming your response, if your article is accepted, you may have the opportunity to make the peer review history publicly available. The record will include editor decision letters (with reviews) and your responses to reviewer comments. If eligible, we will contact you to opt in or out. Please see here for more details:

https://blogs.plos.org/plos/2019/05/plos-journals-now-open-for-published-peer-review/

*PLOS Data Policy*

Please note that as a condition of publication PLOS' data policy (http://journals.plos.org/plosbiology/s/data-availability) requires that you make available all data used to draw the conclusions arrived at in your manuscript. If you have not already done so, you must include any data used in your manuscript either in appropriate repositories, within the body of the manuscript, or as supporting information (N.B. this includes any numerical values that were used to generate graphs, histograms etc.). For an example see here: http://www.plosbiology.org/article/info%3Adoi%2F10.1371%2Fjournal.pbio.1001908#s5

*Blot and Gel Data Policy*

We require the original, uncropped and minimally adjusted images supporting all blot and gel results reported in an article's figures or Supporting Information files. We will require these files before a manuscript can be accepted so please prepare them now, if you have not already uploaded them. Please carefully read our guidelines for how to prepare and upload this data: https://journals.plos.org/plosbiology/s/figures#loc-blot-and-gel-reporting-requirements

*Protocols deposition*

To enhance the reproducibility of your results, we recommend that if applicable you deposit your laboratory protocols in protocols.io, where a protocol can be assigned its own identifier (DOI) such that it can be cited independently in the future. Additionally, PLOS ONE offers an option for publishing peer-reviewed Lab Protocol articles, which describe protocols hosted on protocols.io. Read more information on sharing protocols at https://plos.org/protocols?utm_medium=editorial-email&utm_source=authorletters&utm_campaign=protocols

Thank you again for your submission to our journal. We hope that our editorial process has been constructive thus far, and we welcome your feedback at any time. Please don't hesitate to contact us if you have any questions or comments.

Sincerely,

Roli Roberts

Roland Roberts, PhD

Senior Editor

PLOS Biology

rroberts@plos.org

------------------------------------

REVIEWERS' COMMENTS:

Reviewer #1:

In the manuscript by Liu and Zheng, the authors identified and studied a toxin-antidote (TA) system in C. elegans, which they called sept-1/zina-1. A recent paper (Zdraljevic et al., elife, 2025 preprint v1) has reported the same TA system with a different name mll-1/smll-1 in the wild C. elegans strain XZ1516 and explained the suppression of a divergent version of mll-1 in the reference C. elegans strain N2 by small RNA silencing. Although Liu and Zheng also identified the TA system in the strain XZ1516, they used different methods and concluded that the divergent version of sept-1 in the strain N2, which they named sept-2, is derived and no longer toxic. Overall, the authors provide solid evidence for most conclusions in the manuscript. The authors should however address the criticisms below.

Major criticisms:

1.Naming of the genes:

a.Because Zdraljevic et al. have identified the same system and reported them first, it would be less confusing for readers and the community if the same names were used in this work. In any case, C. elegans gene names should be registered with genenames@wormbase.org

b.The N2 version of sept-1 (if this gene were called 'sept-1') should be referred to in the format as sept-1(N2) or sept-1 in N2. It is not appropriate to call it sept-2, because sept-1(XZ1516) and sept-1(N2) are divergent alleles of the same gene with no ambiguity relative to their relationship as they are syntenic and single-copy.

2.About the conclusion that SEPT-1(N2) has lost most of its toxicity:

a.The direct evidence is Figure 3D, based on the comparison of a single-copy heat-shock construct. The reviewer could not find the number of assayed animals and whether the data derive from one or several lines. Please also verify that no artificial mutations were introduced into the transgene that may interfere.

b.The reviewer cannot see a single-copy experiment that compares the two alleles under their own or the same sept-1(XZ1516) cis-regulatory region, for example one such as in panel E compared in parallel to XZ1516, with clear indication of number of animals. (Recoding to remove potential piRNA sites in panel G may also affect the level of expression so the authors cannot use this experiment to conclude that SEPT-1(N2) has lost toxicity.)

c.Interpretation: To the reviewer, this study does not necessarily "contradict" (line 216) that of Zdraljevic et al but instead they may reflect quantitative, context-dependent effects. In Zdraljevic et al., the experimental approach appears to drive substantially higher expression levels than the method used here, which could account for the stronger or qualitatively different phenotypes observed. In the Figure 3D in this study, long exposure to heat-shock did cause larval arrest.

d.Wording about the "small RNA-mediated suppression" is unclear. Maybe adding "mechanism" at the end would help. For the reader to understand the context, it would be good that you expand a little more about what is known (presence of piRNAs, small RNA sequence data, etc.). Other small RNA pathways than those started by primary piRNAs may be active here. Are the small RNAs actually removed when the gene is recoded?

e.Is sept-1(N2) expressed at a detectable level (using available expression data from the Andersen laboratory, in comparison with XZ1516)? Is its expression altered by recoding? line 221 refers to Fig. 3F but this is just the design of the recoding not the proof that sept-2 is expressed as suggested by the sentence.

3. About sept-1 expression pattern: Transgenesis methods for the expression reporters are not described. Are they multicopy reporter or single-copy insertions like the rescue constructs? From the strain list, they seem to be multicopy. The expression site in somatic cells differs from that reported in Zdraljevic et al. (germline) using smFISH. Maybe this is due to the technique involved as transgenes are more likely silenced in the germline, especially if multicopy. This does not seem to be a good choice and the caveat should at least be stated clearly. It is likely that the expression of single-copy transgenes is too weak to see it and that smFISH is indeed more appropriate.

4. The distribution of TAs can be summarized using a tree representation as in Figure 6. However, C. elegans populations do recombine so the tree representation may not reflect the history of the genomic region involved. The grouping in Hawaiian 1 and 2 seems artificial. It is thus not appropriate to draw evolutionary conclusions about which allele is ancestral, just based on this. In addition:

a.Strain names cannot be seen in Figure 6. Please provide a supplemental file including the detailed information about the three TA existence status for the 611 C. elegans strains. Please also include notes about whether the version likely lost function, as in sept-1(N2) and sup-35(XZ1516).

b.In Figure 6, how similar or divergent the sequence needs to be to the reference to be identified as "present" or "absent"? Maybe using gradient color? Please also include this information in the above supplemental file.

c.Revise accordingly the two last paragraphs of the Discussion. Regarding the last paragraph, please note that some co-occurring TAs have been found in C. tropicalis (Noble et al. 2021, Ben-David et al. 2021).

5. The reviewer strongly suggests completely removing the last section of the Results and Figure 7. They cannot see evidence for a selective sweep marked by a lower pi diversity of the sept-1 allele. Fig. 7 rather show an increase in pi in this region and in any case the region is too large to conclude.

6. The reviewer does not understand the Bayes Empirical Bayes analysis concluding to positive selection on the antidote gene in Fig. 2: in the context of these C. elegans polymorphisms, can you explain and provide methods? None is referenced.

Minor criticisms:

1.Provide the sequences of the constructs and CRISPR alleles.

2.Table S4 "Cloning primers" is empty.

3.In all figures or legends, please indicate the number of animals examined.

4.Figure 1H, y axis label should read "Percentage of animals" (idem in other figures)

5.Figure 1I, the y-axis is confusing (no legend explaining the solid line versus the dots).

6.Figure 2C and line 165: explain what you mean by identity (protein sequence?) and similarity (DNA level?). Typo on "similairy" on figure.

7.Figure 4, the stem-loop part is perhaps interesting but a little distracting from the main topic of the paper.

8.Line 90: remove the last sentence of the Introduction that is highly speculative.

9.line 221: rephrase. This is the rationale but you do not show that the expression is increased. (as mentioned above)

10.Line 314: remove "ecological".

11.Line 383: add the allele/strain names for SEPT-1

12.Line 388: what is the evidence for stabilization in the germline?

13.Line 496: provide the source of the genomes. Idem for RNAseq data when relevant.

14.Typos/grammar in page 14, lines 461 (past tense), 464 (remove "do");

p. 15 lines 484 (to cross them), 485 (isogenic), 486 (crossed), 496 (each other's genome)

p. 16 lines 526 (involved), 538 typo

p. 18 lines 573 "and the"? "plates", 575 (Leica), 580 (Mixed), 581 (strain), 593 (extracted).

p.19 line 608 (resolutions).

Reviewer #2:

In this manuscript, Liu et al. present the genetic mapping and characterization of a novel toxin-antidote (TA) element in C. elegans. This new maternal-effect TA, sept-1/zina-1, was identified in the highly divergent isolate XZ1516 and causes larval lethality in F2 non-carrier individuals. The authors did an excellent job mapping both the toxin- and antidote-encoding genes, and the results are very clean. Although a related element was previously described by the Kruglyak lab (Zdraljevic et al., 2025), the strain collection and overall mapping strategy were different, so the two studies are complementary and together strengthen the conclusions.

Liu et al. also make several additional observations. In particular, they show that expression of the antidote in the embryonic gut is sufficient for rescue, suggesting that the toxin acts in this tissue. They report interesting A-to-I editing of the toxin mRNA, although this does not appear to affect toxic activity. Finally, and most importantly, the authors disagree with Zdraljevic et al. regarding the toxicity and regulation of the highly divergent toxin (sept-2) present in the N2 reference strain.

Overall, the manuscript is clearly written and well organized, but I have several technical comments and some concerns regarding data presentation.

Specific points

1. Toxicity of the N2 toxin (sept-2 / B0250.8 / mll-1)

The main disagreement between this study and Zdraljevic et al. (2025) concerns the intrinsic toxicity of the N2 toxin, B0250.8 (called sept-2 here and mll-1 in Zdraljevic et al.).

Zdraljevic et al. concluded that N2 mll-1 is toxic because overexpression using a tetracycline-inducible system caused abnormal phenotypes in N2, DL238, and XZ1516 backgrounds, similar to those observed upon overexpression of the XZ1516 toxin (sept-1). To explain why toxicity is not normally manifested in the N2 background (despite the absence of a functional antidote), they proposed that N2 mll-1 is repressed by a small-RNA pathway involving secondary 22G-RNAs. Although the evidence for this model is not particularly strong, it is plausible, and it is consistent with previous reports showing that B0250.8 is among the most strongly up-regulated genes in mut-16 mutants.

In contrast, Liu et al. conclude that sept-2 is not capable of poisoning N2. They generated single-copy transgenic lines expressing either sept-1 or sept-2 from a heat-shock promoter and found that a 20-30 min heat shock killed most larvae carrying the sept-1 transgene, whereas sept-2 transgenic larvae were largely unaffected. Interestingly, prolonged heat shock (50 min) of sept-2 transgenics did cause lethality, which the authors interpret as evidence for "weaker toxicity".

These experiments, however, lack a critical control: direct measurement of SEPT-1 and SEPT-2 expression levels. Although MosTI-based integration is reliable for transgene insertion, it does not guarantee expression. The piRNA (21U-RNA) pathway can recognize transgenes and induce somatic and/or germline silencing. Moreover, given prior evidence that sept-2 is regulated by small RNAs, the endogenous pre-existing 22G-RNAs may also target the transgene, resulting in reduced expression. The apparent "weaker toxicity" could therefore simply reflect lower protein levels, and dosage effects are well known in TA systems.

If strong conclusions are to be drawn about the relative toxicity of SEPT-1 and SEPT-2, transgene expression must be directly assessed, for example by western blotting or immunofluorescence. A straightforward approach would be to introduce an N- or C-terminal FLAG (or similar) tag. Importantly, the activity of the tagged protein should first be validated by tagging SEPT-1 at its endogenous locus and confirming that toxicity is retained.

The authors also attempted to test sept-2 toxicity by replacing the sept-1 coding sequence at the endogenous XZ1516 locus with sept-2, using a recoded version to remove putative piRNA target sites. They state that "this recoding likely removed the potential piRNA binding sites in the coding sequence and allowed sept-2 to be expressed, and its toxicity be revealed." However, the manuscript does not clearly describe how this recoding was performed. Which tool was used? Were known piRNA sequences explicitly avoided, or were synonymous mutations introduced more randomly under the assumption that this would disrupt piRNA targeting? This is not clear from the Methods or figure legends.

Furthermore, even if recoding successfully avoided N2 piRNAs (no evidence is provided), piRNA repertoires differ between strains. Because piRNAs are genomically encoded and polymorphic, one cannot assume that a sept-2 sequence recoded based on N2 information will escape silencing in XZ1516. Since the authors conclude that sept-2 is not toxic, it is essential to demonstrate that the recoded gene is in fact expressed in the relevant tissues and stages.

2. Reporting of sample sizes and raw counts

Throughout the manuscript, I could not find clear information on N (number of independent experiments) and n (sample size per experiment) for most crosses and assays. Only percentages and p-values are typically reported. For reproducibility and transparency, I strongly encourage the authors to provide raw counts and full experimental details in the supplementary material and to reference sample sizes consistently in the main text and figure legends.

3. Direction of genetic crosses

Related to the previous point, the direction of many crosses is not specified. For example, in the initial result showing that ~40% of progeny from N2 × XZ1516 crosses display developmental defects, it is unclear whether XZ1516 hermaphrodites were crossed to N2 males or vice versa. Previous work (Pliota et al., 2024) has demonstrated that cross direction can critically affect TA activity in C. tropicalis; for instance, the maternal-effect slow-1/grow-1 system is only active when paternally inherited. For completeness and clarity, the direction of all crosses should be explicitly stated.

4. Identity and nomenclature of sept-1/zina-1 and mll-1/smll-1

At line 183, the authors state that the TA system described by Zdraljevic et al. is "likely the same as" sept-1/zina-1. There is, in fact, no ambiguity here: sept-1/zina-1 and mll-1/smll-1 correspond to the same locus and should be described as such.

In addition, I am not sure whether it is advisable to introduce new gene names (sept-1 and zina-1) in this context. In general, redundancy in gene nomenclature should be avoided whenever possible. Here, it is clear that the genes in question are identical to those previously described, and there seems to be no strong justification for maintaining two parallel naming schemes for the same TA system.

5. Mutual exclusivity of maternal TA systems

At line 417, the authors note that sept-1/zina-1 and sup-35/pha-1 are almost mutually exclusive, and suggest that this may reflect an energetic cost of maintaining two maternal TA systems. While the observation is interesting, previous work (Ben-David et al., Current Biology, 2021) showed that some C. tropicalis isolates (e.g., NIC203 and EG6180) carry two or even three active maternal-effect TAs, arguing against a strong general fitness cost of harboring multiple such systems. The apparent anti-correlation between sup-35/pha-1 and sept-1/zina-1 could therefore be coincidental. I would recommend either toning down this interpretation or, if the authors wish to emphasize it, directly testing the hypothesis by comparing the fitness of NILs carrying both TA systems with appropriate single-TA and double-knockout controls. Overall, claims about the relative age of the TAs should be toned down. The evidence is rather indirect.

6. SEP-domain

The authors named the toxin after its SEP-domain. I may have missed this, but there was no reference or explanation to what is known about this protein domain.

Reviewer #3:

[identify themselves as Daniel Park and Michael Ailion]

Toxin-antidotes (TAs) promote their own inheritance with little or no benefit to their host. TAs are widespread in prokaryotes, and they are now being found and characterized in eukaryotes. In this manuscript, the authors identify and characterize a new TA in a divergent Hawaiian strain of C. elegans, the third one found in this species. Strong genetic evidence is provided that the sept-1 toxin is maternally transmitted and causes larval arrest in the absence of its zygotically expressed antidote zina-1. Additional convincing experiments show that in the standard lab strain N2, the toxin gene has been retained but has lost toxicity due to sequence divergence, while the antidote has become pseudogenized. The manuscript concludes with an interesting phylogenetic analysis of the three known TAs in C. elegans, suggesting that the two known maternal effect TAs do not coexist in the same strain. Overall, the work is performed well and most of the conclusions are supported by the data. This is an interesting and solid new addition to the growing literature in the field of eukaryotic TAs.

Considerations

1. The larval arrest phenotype is described as "rod-like." This designation has previously been used to describe the larval arrest phenotype of mutants in the Ras pathway, due to malfunction of the excretory duct system and defects in osmoregulation. But it is not clear whether the larval arrest phenotype due to sept-1 toxicity is this same rod-like phenotype. First, the arrested larvae shown in this manuscript are not straight rods, but have a curved posture (e.g. Fig 5C&D and Fig S2A). Second, given that the toxins causes defects in the morphology of the intestine and the zina-1 antidote acts in the intestine, it is unclear whether this is equivalent to the previously described rod-like arrest of mutants that affect the excretory system. This issue warrants further discussion, and without further evidence, it would be prudent to use a term other than "rod-like" for the arrest phenotype.

2. It is unclear if sept-1 is only capable of toxicity at the L1 larval stage. What happens if animals carrying hs::sept-1 are heat-shocked as older animals? Is there any evidence for toxicity in tissues other than the intestine?

3. Lines 279-284 and Figure S5. Based on images of sept-1p and sept-2p transcriptional reporters, it is concluded that sept-1 is expressed at a higher level. This is not convincing and the conclusion should be toned down. First, there are only single images with no quantification. Second, these strains carry extrachromosomal arrays, which are overexpressed and may be at different copy levels. There is often a lot of variability between independent arrays of the same construct, so it is not meaningful to quantitatively compare individual arrays of two different constructs. It is unclear how many arrays of each construct were generated, but quantitative analysis of a large number of arrays of each construct would be necessary to even begin to be meaningful. Single-copy insertions at the same chromosomal locus would provide stronger evidence.

4. The conclusions in the final section of the manuscript about how TA systems influence genetic diversity are not convincing. In Fig 7, sept-1(-) has a slightly higher rate of polymorphisms at the sept-1/zina-1 locus compared to sept-1(+), yet there is an even larger increase in polymorphisms further to the right, away from the locus at Mb 20.59-20.62. But at Mb 20.50-20.53 (between sept-1/zina-1 and the peak at Mb 20.60), sept-1(+) has higher polymorphisms than sept-1(-). It is unclear whether any of these effects are statistically significant. In fact, the absence of sup-35 appears to have a greater effect on polymorphisms at the sept-1/zina-1 locus than the absence of sept-1. The analysis of Tajima's D statistic is also unconvincing. As with the analysis of polymorphism rate, there is no statistical analysis to determine whether the small effects seen are statistically significant. In fact, if anything, sup-35(-) appears to have a more negative Tajima's D than sup-35(+) near the sup-35/pha-1 locus which is the opposite of what is stated in lines 357-359, "Supporting this idea, we found more negative Tajima's D in the region linked to sept-1(+) or sup-35(+) compared to the same region in strains without the TA system." Overall, the data presented here do not support the conclusion that the presence of a TA leads to reduced diversity.

5. In general, the Methods sections are brief and not very specific. The following Methods sections should be improved:

a. Concentrations of DNAs injected to make transgenic lines should be provided. This information could be added to Table S2. Co-injection markers should be specified.

b. For CRISPR, more details should be provided, including concentrations of reagents and what co-CRISPR marker was used. Primers used to generate repair templates do not seem to be provided in Table S4. Also, it is stated that PCR genotyping was used to screen for CRISPR edits, but it is unclear what primers were used. These primers do not seem to be provided in Table S4. There are some genotyping primers shown in Table S4, but these are presumed to be just the primers used to genotype the sept-1, sept-2, and zina-1 alleles, not for screening CRISPR edits. What these are should be clarified.

c. Heat-shock temperature is not provided.

d. Methods of cloning and generating DNA constructs are not described fully. What cloning methods were used? Also, cloning primers should be listed in Table S4.

e. Antibody staining: concentrations of antibodies should be provided as well as the buffer they are diluted into.

f. smFISH: concentrations and sequences of probes should be provided.

g. RNAseq: description of how worms were grown and RNA isolated should be provided.

Minor points

1. The sept-1/zina-1 TA has also been reported by another lab (Zdraljevic et al., PMID: 39605437) who called it mll-1/smll-1. At two points, (lines 185 & 365), sept-1/zina-1 is described as "likely the same" as mll-1/smll-1. Use of the term "likely" is confusing, as the two papers identify the same genes.

2. A rather lengthy section of the results is devoted to RNA editing in the 3'UTR of the sept-1 mRNA. This section is tangential to the main storyline and ultimately concludes with a negative result: RNA editing has little effect on the sept-1 mRNA stability and is not required for toxicity. We feel that this section could be dramatically shortened, with the data in a supplementary figure where they won't distract from the main narrative.

3. Discussion, Lines 387-391. This paragraph makes unsupported claims about the promoter of sept-1 being stronger than sept-2 (see point #3 above), and the importance of the sept-1 3'UTR stem-loop and RNA-editing to stabilizing sept-1 mRNA. The data however show that RNA editing does not significantly stabilize the sept-1 mRNA and whether the stem-loop is stabilizing is not addressed. We would suggest cutting this paragraph.

4. Discussion, Lines 392-401. This is a highly speculative paragraph that makes assertions based on data not described in the Results sections. Line 393: it is asserted that ZINA-1 is not localized in the nucleus, but this has not been convincingly demonstrated. The images in Fig 5A&B are not shown at the resolution needed to make such a claim. Line 397: it is asserted that GFP::ZINA-1 forms aggregates. It is unclear what is considered an aggregate in Fig 5A&B - arrows in these figures would help. There are perhaps some punctae, but proteins can appear punctate for reasons other than aggregation (e.g. membrane association). Additionally, zinc-finger proteins are not exclusively DNA or RNA-binding proteins as implied here. They can also bind other proteins and function in a wide range of processes (see PMID 29152378 for example). Thus, ZINA-1 could have a number of potential antidote functions outside the nucleus that don't involve RNAs. Based on the data, hypothesizing that ZINA-1 is involved in liquid-liquid phase separation is highly speculative.

5. sept-1p shows somatic expression in the intestine and pharynx in both L1 larvae and adults (Fig S5). This is not discussed. Is it possible that, in addition to maternally-delivered sept-1, somatic expression also contributes to toxicity? Also, is the endogenously-tagged sept-1::tagRFP expressed in larvae and adults? If so, where does the protein localize?

6. For the crosses in Fig 1, it would be useful to show the parental generation cross used to generate the N2/XZ1516 hybrid -- i.e. which parent was male and which was hermaphrodite? Does the direction of the parental cross matter? Given that some maternally inherited incompatibility genes show a maternal cytoplasmic pattern of inheritance, that possibility should be accounted for here.

7. Line 163. What is a SEP domain and generally what do they do? Providing a domain map of SEPT-1 would be useful.

8. Outside of C. elegans, are there any homologs of sept-1 and zina-1?

9. Lines 404-406: "Hawaiian_2 strains represent a transition period when the sept-1/zina-1 was gradually lost through the accumulation of missense mutations in the sept-1 coding sequences." This implies that these strains have an intermediate number of missense variants. Is this true?

10. Lines 414-416: as an alternative to the idea that peel-1/zeel-1 emerged early in the Hawaiian lineage but didn't propagate, is it possible that peel-1/zeel-1 did not exist in the Hawaiian ancestor, but instead was acquired later in certain Hawaiian strains by mating and recombination with peel-1/zeel-1 strains (i.e. this chromosomal region was transferred while the rest of the genome remained Hawaiian?). It seems that it is tricky to make strong conclusions about the age of a chromosomal segment from the overall phylogenetic tree, because the phylogeny of specific chromosomes or genes may not match the overall tree.

11. Is sept-2 the proper nomenclature for the sept-1 ortholog in N2? They are both the same gene in the same species, only with some sequence divergence. It seems like calling this gene sept-1(N2) might be more appropriate.

12. Line 290 and Figure 5A: Though GFP::ZINA-1 co-localizes with ges-1p::RFP, it does not show "clear enrichment." This should be toned down.

13. Line 300 & Fig 5C legend: why are these worms described as "dried up"? In the classic rod-like larval arrest, worms are typically described as "fluid-filled."

14. Figures or legends should include N of worms scored.

15. All micrograph scale bars should be defined in the figure legends. For example: Fig 3D, Fig 4A, Fig 5B-E.

16. In figure panels with multiple Chi-square tests, there should be statistical correction for multiple comparisons.

17. Figure 6: there appear to be a couple instances where peel-1 is present while zeel-1 is absent. Could the authors comment on this, since peel-1(+)/zeel-1(-) animals should not be viable.

18. Figure 6: it would be useful to provide strain names in a supplementary Table for the 611 worm strains and their TA genotypes.

19. There should be a key for Table S1, explaining what each of the column headings means. Many of these are nonintuitive.

20. In Table S4, CRISPR repair templates should be briefly described, not just "recombinant DNA."

21. Table S5 should list the vector backbones of the plasmid constructs.

22. In many of the micrographs, it would help non-worm readers if the images included arrows pointing to specific features of interest. For example, pointing out the deformed pharynx in Fig 1A.

23. Figure 1A: for clarity, the figure should say "Type I" or "Type II," not "Arrested larva-I" or "Arrested larva-II"

24. Figure 1B-F: a Punnett square showing the result of the cross and how/why each case of deviance from 75% marker is informative would be useful, especially for readers from non-genetics backgrounds.

25. In Fig S6, shading of identical and similar residues would help to visualize the alignment.

26. The use of a white font on a gray background in Fig 7A is hard to read.

27. In Fig 7B (left panel), it would help to use colors that are more different than blue and green.

28. In Fig S1D & E: why are there male symbols in the final generation when worms were scored? Were only male worms scored?

29. Line 125: make it clear that 15-22 Mb refers to chromosomal position, not the size of the mapped region because this was confusing when first read.

30. Line 141: change "The rest exons…" to "The rest of the exons…"

31. Line 221: "allowed sept-2 to be expressed and its toxicity be revealed." The writing here is confusing and makes it sound like sept-2 is toxic, when in fact, the opposite is concluded. Better would be "allowed sept-2 to be expressed and its possible toxicity to be tested."

32. Line 247-248: sept-1 mRNA being deposited into oocyte is also reported by Zdraljevic et al., and this should be noted as a similarity between the studies.

33. Line 299: DIC should be spelled out.

34. Lines 354 and 359: include which panel of Figure 7.

35. Line 381: is it correct to call this the "endogenous RNAi pathway"?

36. Line 782: include reference for Bayes Empirical Bayes analysis in the figure text. This method should also be described in the Methods section.

37. There are several incorrect figure call-outs:

line 179: not Fig S2C

line 303: should include Fig 5E

lines 321 & 330: there are no Fig 6A & B, just Fig 6.

38. There are a number of typos:

line 38: should be "addiction"

line 383: should be "between SEPT-1 and SEPT-2"

line 451: should be "Center"

line 520: should be "IPTG"

line 538: should be "substitution"

line 575: should be "Leica"

line 581: should be "strain"

line 594: the period should be changed to a comma: "…wild isolates, we combined…".

Reviewed (and signed) by Daniel Park and Michael Ailion

# Author comment: Author response to Decision Letter 2

**DOI:** 10.1371/journal.pbio.3003563.r003

17 Jun 2026

Attachment Submitted filename: Point to point response.docx

# Editor report: Decision Letter 2

**DOI:** 10.1371/journal.pbio.3003563.r004

**Authors:** Roland G Roberts

2 Jul 2026

Dear Dr Zheng,

Thank you for your patience while we considered your revised manuscript "sept-1/zina-1 is an Ancient Toxin-Antidote System in Caenorhabditis elegans" for publication as a Research Article at PLOS Biology. This revised version of your manuscript has been evaluated by the PLOS Biology editors and the Academic Editor.

Based on our Academic Editor's assessment of your revision, we are likely to accept this manuscript for publication, provided you satisfactorily address the following data and other policy-related requests.

IMPORTANT - please attend to the following:

a) Please address my Data Policy requests below; specifically, we need you to supply the numerical values underlying Figs 1DEFGHI, 2DEF, 3ACDEGH, 4D, S2C, S3A, S5ACD S6AB, S7 (treefile), either as a supplementary data file or as a permanent DOI’d deposition.

b) Please cite the location of the data clearly in all relevant main and supplementary Figure legends, e.g. “The data underlying this Figure can be found in S1 Data” or “The data underlying this Figure can be found in https://zenodo.org/records/"; also update your Data Availability Statement accordingly.

c) Please make any custom code available, either as a supplementary file or as part of your data deposition.

d) Please include the URLs of your funders in the Financial Disclosure statement.

As you address these items, please take this last chance to review your reference list to ensure that it is complete and correct. If you have cited papers that have been retracted, please include the rationale for doing so in the manuscript text, or remove these references and replace them with relevant current references. Any changes to the reference list should be mentioned in the cover letter that accompanies your revised manuscript.

In addition to these revisions, you may need to complete some formatting changes, which you will receive in a follow up email. A member of our team will be in touch with a set of requests shortly. If you do not receive a separate email within a few days, please assume that checks have been completed, and no additional changes are required.

We expect to receive your revised manuscript within two weeks.

To submit your revision, please go to https://www.editorialmanager.com/pbiology/ and log in as an Author. Click the link labelled 'Submissions Needing Revision' to find your submission record. Your revised submission must include the following:

- a cover letter that should detail your responses to any editorial requests, if applicable, and whether changes have been made to the reference list

- a Response to Reviewers file that provides a detailed response to the reviewers' comments (if applicable, if not applicable please do not delete your existing 'Response to Reviewers' file.)

- a track-changes file indicating any changes that you have made to the manuscript.

NOTE: If Supporting Information files are included with your article, note that these are not copyedited and will be published as they are submitted. Please ensure that these files are legible and of high quality (at least 300 dpi) in an easily accessible file format. For this reason, please be aware that any references listed in an SI file will not be indexed. For more information, see our Supporting Information guidelines:

https://journals.plos.org/plosbiology/s/supporting-information

*Published Peer Review History*

Please note that you may have the opportunity to make the peer review history publicly available. The record will include editor decision letters (with reviews) and your responses to reviewer comments. If eligible, we will contact you to opt in or out. Please see here for more details:

https://plos.org/published-peer-review-history/

*Press*

Should you, your institution's press office or the journal office choose to press release your paper, please ensure you have opted out of Early Article Posting on the submission form. We ask that you notify us as soon as possible if you or your institution is planning to press release the article.

*Protocols deposition*

To enhance the reproducibility of your results, we recommend that if applicable you deposit your laboratory protocols in protocols.io, where a protocol can be assigned its own identifier (DOI) such that it can be cited independently in the future. Additionally, PLOS ONE offers an option for publishing peer-reviewed Lab Protocol articles, which describe protocols hosted on protocols.io. Read more information on sharing protocols at https://plos.org/protocols?utm_medium=editorial-email&utm_source=authorletters&utm_campaign=protocols

Please do not hesitate to contact me should you have any questions.

Sincerely,

Roli Roberts

Roland Roberts, PhD

Senior Editor

rroberts@plos.org

PLOS Biology

------------------------------------------------------------------------

DATA POLICY:

You may be aware of the PLOS Data Policy, which requires that all data be made available without restriction: http://journals.plos.org/plosbiology/s/data-availability. For more information, please also see this editorial: http://dx.doi.org/10.1371/journal.pbio.1001797

Note that we do not require all raw data. Rather, we ask that all individual quantitative observations that underlie the data summarized in the figures and results of your paper be made available in one of the following forms:

1) Supplementary files (e.g., excel). Please ensure that all data files are uploaded as 'Supporting Information' and are invariably referred to (in the manuscript, figure legends, and the Description field when uploading your files) using the following format verbatim: S1 Data, S2 Data, etc. Multiple panels of a single or even several figures can be included as multiple sheets in one excel file that is saved using exactly the following convention: S1_Data.xlsx (using an underscore).

2) Deposition in a publicly available repository. Please also provide the accession code or a reviewer link so that we may view your data before publication.

Regardless of the method selected, please ensure that you provide the individual numerical values that underlie the summary data displayed in the following figure panels as they are essential for readers to assess your analysis and to reproduce it: Figs 1DEFGHI, 2DEF, 3ACDEGH, 4D, S2C, S3A, S5ACD S6AB, S7 (treefile). NOTE: the numerical data provided should include all replicates AND the way in which the plotted mean and errors were derived (it should not present only the mean/average values).

IMPORTANT: Please also ensure that figure legends in your manuscript include information on where the underlying data can be found, and ensure your supplemental data file/s has a legend.

Please ensure that your Data Statement in the submission system accurately describes where your data can be found.

------------------------------------------------------------------------

CODE POLICY

Per journal policy, if you have generated any custom code during the course of this investigation, please make it available without restrictions. Please ensure that the code is sufficiently well documented and reusable, and that your Data Statement in the Editorial Manager submission system accurately describes where your code can be found. More information on our Code Policy, what and how to share can be found here: https://journals.plos.org/plosbiology/s/code-availability

Please note that we cannot accept sole deposition of code in GitHub, as this could be changed after publication. However, you can archive this version of your publicly available GitHub code to Zenodo. Once you do this, it will generate a DOI number, which you will need to provide in the Data Accessibility Statement (you are welcome to also provide the GitHub access information). See the process for doing this here: https://docs.github.com/en/repositories/archiving-a-github-repository/referencing-and-citing-content

------------------------------------------------------------------------

BLOT AND GEL REPORTING REQUIREMENTS:

We require the original, uncropped and minimally adjusted images supporting all blot and gel results reported in an article's figures or Supporting Information files. We will require these files before a manuscript can be accepted so please prepare and upload them now. Please carefully read our guidelines for how to prepare and upload this data: https://journals.plos.org/plosbiology/s/figures#loc-blot-and-gel-reporting-requirements

------------------------------------------------------------------------

DATA NOT SHOWN?

- Please note that per journal policy, we do not allow the mention of "data not shown", "personal communication", "manuscript in preparation" or other references to data that is not publicly available or contained within this manuscript. Please either remove mention of these data or provide figures presenting the results and the data underlying the figure(s).

------------------------------------------------------------------------

# Author comment: Author response to Decision Letter 3

**DOI:** 10.1371/journal.pbio.3003563.r005

9 Jul 2026

Attachment Submitted filename: Point_to_point_response_auresp_3.docx

# Editor report: Decision Letter 3

**DOI:** 10.1371/journal.pbio.3003563.r006

**Authors:** Roland G Roberts

13 Jul 2026

Dear Dr Zheng,

Thank you for the submission of your revised Research Article "sept-1/zina-1 is an Ancient Toxin-Antidote System in Caenorhabditis elegans" for publication in PLOS Biology. On behalf of my colleagues and the Academic Editor, Peter Sarkies, I'm pleased to say that we can in principle accept your manuscript for publication, provided you address any remaining formatting and reporting issues. These will be detailed in an email you should receive within 2-3 business days from our colleagues in the journal operations team; no action is required from you until then. Please note that we will not be able to formally accept your manuscript and schedule it for publication until you have completed any requested changes.

IMPORTANT: I've asked my colleagues to include the following request alongside their own: "Many thanks for suppying your code in Github. However, because Github depositions can be readily changed or deleted, please make a permanent DOI’d copy (e.g. in Zenodo) and provide this URL in your Data Availability Statement."

Please take a minute to log into Editorial Manager at http://www.editorialmanager.com/pbiology/, click the "Update My Information" link at the top of the page, and update your user information to ensure an efficient production process.

PRESS: We frequently collaborate with press offices. If your institution or institutions have a press office, please notify them about your upcoming paper at this point, to enable them to help maximise its impact. If the press office is planning to promote your findings, we would be grateful if they could coordinate with biologypress@plos.org. If you have previously opted in to the early version process, we ask that you notify us immediately of any press plans so that we may opt out on your behalf.

We also ask that you take this opportunity to read our Embargo Policy regarding the discussion, promotion and media coverage of work that is yet to be published by PLOS. As your manuscript is not yet published, it is bound by the conditions of our Embargo Policy. Please be aware that this policy is in place both to ensure that any press coverage of your article is fully substantiated and to provide a direct link between such coverage and the published work. For full details of our Embargo Policy, please visit http://www.plos.org/about/media-inquiries/embargo-policy/.

Thank you again for choosing PLOS Biology for publication and supporting Open Access publishing. We look forward to publishing your study.

Sincerely,

Roli Roberts

Roland G Roberts, PhD, PhD

Senior Editor

PLOS Biology

rroberts@plos.org
